# Generation of functional noncanonical donor splice sites by +2T variants in breast cancer susceptibility genes: impact on clinical interpretation

**DOI:** 10.1002/path.6497

**Published:** 2025-11-13

**Authors:** Inés Llinares‐Burguet, Lara Sanoguera‐Miralles, Elena Bueno‐Martínez, Alicia García‐Álvarez, Alberto Valenzuela‐Palomo, Pedro Pérez‐Segura, Miguel de la Hoya, Eladio A Velasco‐Sampedro

**Affiliations:** ^1^ Splicing and genetic susceptibility to cancer. Instituto de Biomedicina y Genética Molecular de Valladolid (IBGM). Consejo Superior de Investigaciones Científicas ‐ Universidad de Valladolid (CSIC‐UVa) Valladolid Spain; ^2^ Molecular Oncology Laboratory, Hospital Clínico San Carlos, IdISSC (Instituto de Investigación Sanitaria del Hospital Clínico San Carlos) Madrid Spain

**Keywords:** hereditary breast cancer, susceptibility genes, noncanonical splicing, +2T variants, aberrant splicing, minigenes, variant classification

## Abstract

Splicing dysregulation is a relevant mechanism of pathogenicity for variants in disease susceptibility genes. Variants affecting the critical intronic +1 and +2 GT nucleotides of the 5’ splice sites (5'ss) are generally strong indicators of pathogenicity. However, some +2 T variants create functional noncanonical 5'ss that generate wildtype transcripts, hampering accurate variant interpretation and genetic counseling. We previously showed that variants *PALB2* c.108+2T > C and *ATM* c.1898+2T > G generated significant levels of full‐length (FL) transcripts by creating functional atypical GC and GG donor sites, respectively. In this study, we aimed to investigate the splicing impact of +2T variants in the breast cancer susceptibility genes *ATM*, *BRCA1*, and *PALB2*. For this purpose, five minigenes encompassing 29 exons of *ATM*, *BRCA1*, and *PALB2* were employed. A total of 30 +2T > C/G/A variants were introduced into these constructs by site‐directed mutagenesis and analyzed in MCF‐7 cells. Four +2T > C variants (*ATM* c.6347+2T > C, *BRCA1* c.5193+2T > C and c.5277+2T > C, and *PALB2* c.2748+2T > C) and *ATM* variants c.6347+2T > A/G produced FL‐transcripts (4%–81% of the overall expression). All +2T > C leaky variants conserved a central core of 6 nucleotides (AGgcaa). Variants were assessed according to the ClinGen specifications of the American College of Medical Genetics and Genomics/Association for Molecular Pathology (ACMG/AMP) interpretation guidelines. Two variants (*ATM* c.6347+2T > C and *BRCA1* c.5193+2T > C) were classified as likely benign, consistent with predictions based on their respective ACMG/AMP‐based gene specifications. Conversely, two variants (*ATM* c.6347+2T > G and *BRCA1* c.4675+2T > C), initially predicted as likely pathogenic, were reclassified as variant of uncertain significance (VUS). In conclusion, a significant proportion of +2T variants can create functional noncanonical 5'ss, resulting in the production of FL‐transcripts that may preserve gene function. Variant‐splicing assays provide essential data for accurate clinical classification and for the development of effective clinical management strategies for patients and their families. © 2025 The Author(s). *The Journal of Pathology* published by John Wiley & Sons Ltd on behalf of The Pathological Society of Great Britain and Ireland.

## Introduction

RNA splicing is an essential and highly regulated gene expression step, orchestrated by the spliceosome that recognizes specific conserved sequence motifs, such as the 5’ and 3’ splice sites (5'ss or donor site and 3'ss or acceptor site, respectively), that define exon–intron boundaries. Accurate exon recognition often relies on the participation of supplementary *cis*‐regulatory sequences, such as splicing enhancers and silencers, which recruit *trans*‐acting positive and negative regulatory factors, respectively [1, 2]. Hence, variant‐induced splicing anomalies in human disease genes is a frequent cause of genetic disorders [3, 4, 5, 6].

The GT and AG dinucleotides at the intronic positions ± 1 and ± 2 are the most conserved nucleotides in the splice sites. However, in ~1% of human introns, deviations from the canonical GT‐AG rule occur, with other atypical combinations being recognized and processed by the spliceosome. Among these, the replacement of the dinucleotide GT at the 5'ss by GC is the most frequent, accounting for about 0.9% of human exons [7, 8].

Breast cancer (BC) risk is significantly associated with pathogenic variants in at least eight susceptibility genes: *BRCA1* (MIM#113705), *BRCA2* (MIM#600185), *PALB2* (MIM#610355), *ATM* (MIM#607585), *CHEK2* (MIM# 604373), *BARD1* (MIM#601593), *RAD51C* (MIM#602774), and *RAD51D* (MIM#602954) [9, 10]. For most of these, we have conducted comprehensive splicing studies by minigenes assays [5, 11, 12, 13, 14, 15, 16]. Interestingly, *ATM* exon 50, *PALB2* exon 12, and *BRCA2* exon 17 have natural GC‐5'ss. These motifs display a high degree of conservation beyond the core GC dinucleotide (MAGgcaagt) [11, 14, 17] that enhances the complementarity with U1 snRNA, facilitating efficient exon definition [18].

GC‐donor sites are especially common in alternatively‐spliced introns. Their recognition can be mediated by splicing regulatory elements (SRE) to compensate for their intrinsic weakness. Indeed, competent recognition of GC‐sites has been reported to be mediated by higher densities of splicing enhancers and lower densities of silencers [18, 19]. For instance, several splicing factors, including SRSF7, SRSF2, Tra2β, and SRSF1, are implicated in recognizing the natural GC‐5'ss of *BRCA2* intron 17, as well as the GC‐5'ss activated by a variant in the *BTK* gene (MIM#300300) [18, 20].

Remarkably, variants at positions ± 1 and ± 2 were initially considered a very strong signal of pathogenicity according to the American College of Medical Genetics and Genomics/Association for Molecular Pathology (ACMG/AMP) recommendations [21]. In this context, one important question to address is whether +2T > C changes are capable of generating functional noncanonical GC‐5'ss. Such genetic variants could enable successful exon recognition and potentially preserve gene function. In fact, around 15%–18% of +2T > C changes create active GC‐donors that produce variable amounts of the expected canonical transcript [22]. In keeping with that, we showed that *PALB2* variant c.108+2T > C and *ATM* c.1898+2T > G, initially classified as likely pathogenic in ClinVar, generated 86% and 13%, respectively, of their corresponding full‐length transcripts (supplementary material, Table S1) [14, 15]. These findings may imply changes in the clinical interpretation of variants and possibly a reformulation of the classification guidelines. In fact, *PALB2* c.108+2T > C was reclassified as a variant of uncertain significance (VUS), which entails substantial modifications in the clinical management of carrier patients and families. Moreover, other +2T > C variants in the BC susceptibility genes, such as *BRCA1* c.5193+2T > C, have been predicted as potential functional GC‐donors by the current criteria specifications for *BRCA1/BRCA2* [23].

In this study we aimed to investigate the capability of +2T variants of generating functional noncanonical 5'ss and their impact on clinical interpretation. For this purpose, 30 +2T >C/G/A variants in the BC susceptibility genes *ATM, BRCA1*, and *PALB2* were assayed in five different minigene constructs and classified according to current ACMG/AMP‐based specifications.

## Materials and methods

### Ethics approval

Ethical approval for this study was obtained from the Ethics Committee of the Spanish National Research Council‐CSIC (28 May 2018).

### Variant and transcript annotations

Variants and splicing events were annotated following the guidelines of the Human Genome Variation Society (HGVS: https://hgvs-nomenclature.org/stable/), using the following MANE Select transcripts: NM_000051.4 (*ATM*); NM_007294.4 (*BRCA1*); NM_024675.4 (*PALB2*). For simplicity, splicing events were designated a short descriptor, as previously described [24].

### Variant selection and bioinformatics analysis

Donor sites of BC genes were bioinformatically analyzed using MaxEntScan (MES) to assess their strength (https://github.com/Congenica/maxentscan; accessed on 12 March 2025) [25], and SpliceAI (genome version: hg38, score type: raw, max distance:10,000) (https://spliceailookup.broadinstitute.org/; accessed on 12 March 2025) [26]. A SpliceAI Donor Loss (DL) score < 0.8 was taken as an indicator of putative functional splice‐sites according to the ACMG/AMP criteria specifications for *BRCA1*/*BRCA2* [23]. Naturally occurring GC‐donors in *ATM* exon 50, *BRCA2* exon 17 and *PALB2* exon 12, together with the functional GC‐donor created by *PALB2* c.108+2T > C (exon 2), all show high similarity to the 5'ss consensus sequence ([C/A]AGgcaagt), suggesting that this may represent a relevant signature of active GC‐5'ss [17]. Therefore, variants from strong GT‐splice sites (MES ≥10.8) and SpliceAI Donor Loss <0.8 were prioritized (supplementary material, Table S2) [23]. Eight variants met both conditions: *ATM* c.1607+2T > C (exon 10), c.6347+2T > C (exon 43) and c.8786+2T > C (exon 60), *BRCA1* c.5193+2T > C (exon 18), *BRCA2* c.793+2T > C (exon 9) and c.8953+2T > C (exon 22) and *PALB2* c.108+2T > C (exon 2) and c.2748+2T > C (exon 7). We focused our analysis on variants *ATM* c.6347+2T > C, *BRCA1* c.5193+2T > C and *PALB2* c.2748+2T > C. Notably, *BRCA1* c.5193+2T > C and *ATM* c.6347+2T > C were predicted to create functional GC sites according to the *BRCA1/BRCA2* and *ATM* expert panel criteria specifications, respectively [23, 27].

Finally, binding of splicing factors was predicted with the deep‐learning approach DeepCLIP (https://deepclip-web.compbio.sdu.dk/; accessed on 12 March 2025) [28].

### Minigene construction and mutagenesis

**Figure 1:**
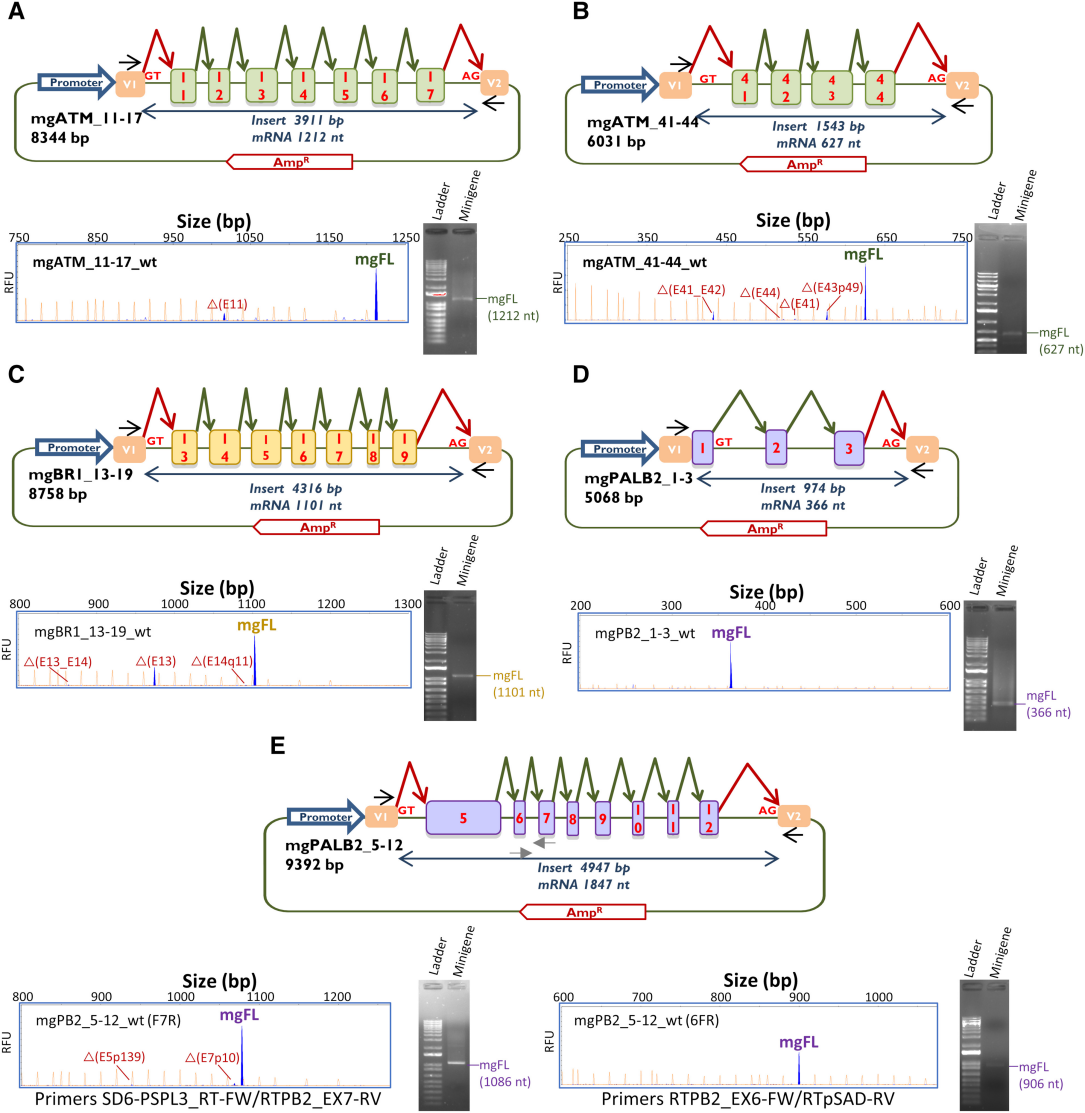
Schematic representation and splicing assay of the wildtype minigenes (A) mgATM_11–17 (B) mgATM_41–44 (C) mgBRCA1_13–19 (D) mgPALB2_1–3, and (E) mgPALB2_5–12. Exons are indicated by boxes; green elbow arrows indicate the expected splicing reactions, and black arrows denote the specific vector RT‐PCR primers. RT‐PCR products were analyzed by agarose gel electrophoresis (right) and fluorescent fragment electrophoresis (left) where transcripts are indicated by blue peaks (FAM‐labeled products) and orange peaks represent the LIZ600/LIZ1200 size standards (mgFL, expected minigene full‐length transcript). RFU (*y*‐axes), relative fluorescence units.

The inserts of minigenes mgATM_41–44 (*ATM* exons 41 to 44) and mgBRCA1_13–19 (*BRCA1* exons 13 to 19; legacy exons 14 to 20) were designed in‐house using the corresponding reference sequence and were cloned into the splicing vector pSAD v9.0 (Patent P201231427‐CSIC) [29, 30]. Constructs, insert sequences, and primers are described in Figure 1 and supplementary material, Figure S1 and Table S3. The final constructs were confirmed by sequencing (Macrogen, Madrid, Spain). In addition, minigenes mgATM_11–17, mgPALB2_1–3, and mgPALB2_5–12 were used in this study [14, 15, 31].

Nineteen +2T > C variants were introduced into the wildtype (wt) minigenes by site‐directed mutagenesis using the QuikChange Lightning kit following the manufacturer's protocol (Agilent, Santa Clara, CA, USA) and specific primers (supplementary material, Table S3). To test the functionality of atypical GA and GG donors, a further six +2T > A and five +2T > G variants were genetically engineered into the corresponding minigenes. All mutant constructs were confirmed by sequencing (Macrogen).

### Minigene splicing assays

Approximately 2 × 10^5^ cells of the human breast cancer cell lines MCF‐7 (ATCC HTB‐22, LGC Standards, Barcelona, Spain) were grown in 0.5 ml of medium (MEM, 10% fetal bovine serum, 1% nonessential amino acids, and 1% penicillin/streptomycin stock solution) to 90% confluency in four‐well plates (Nunc, Roskilde, Denmark).

Cells were transfected with 1 μg of wt/mutant minigenes using 2 μl of Lipofectamine‐LTX (Life Technologies, Carlsbad, CA, USA). At 48 h after transfection, the nonsense‐mediated decay (NMD) process was inhibited with cycloheximide 300 μg/ml (Sigma‐Aldrich, St. Louis, MO, USA) for 4 h before RNA extraction. To check reproducibility of the splicing outcomes, the *ATM* c.6347+2T > C/G and *BRCA1* c.5193+2T > C variants were assayed in MDA‐MB‐231 cells (ATCC HTB‐26, LGC Standards), grown under the same conditions as MCF‐7 cells.

RNA was purified with the Genematrix Universal RNA Purification Kit (EURx, Gdansk, Poland) following the manufacturer's protocol using on‐column DNAse I treatment. Reverse transcription was carried out using 400 ng RNA and the RevertAid First Strand cDNA Synthesis Kit (Life Technologies) following the manufacturer's protocol. Minigene RNA was specifically retrotranscribed through the use of the vector‐specific primer RTPSPL3‐RV (5’‐TGAGGAGTGAATTGGTCGAA‐3’).

The resulting cDNAs of mgATM_41–44 and mgBRCA1_13–19 were amplified using Platinum‐Taq DNA polymerase (Life Technologies) and primers SD6‐PSPL3_RT‐FW (5’‐TCACCTGGACAACCTCAAAG‐3’) and RTpSAD‐RV (patent P201231427) under the following thermocycling conditions: 94 °C, 2 min +35 cycles × [94 °C,0 seg/60 °C, 30 seg/72°C, 1 min/kb] +72 °C, 5 min. The mgPALB2_5–12 cDNA was amplified with two different primer pairs: SD6‐PSPL3_RT‐FW/RTPB2_EX7‐RV (5’‐AAACTACATCTTCGCAAGCA‐3’) and RTPB2_EX6‐FW (5’‐ GATAGCATAAACCCAGGCA‐3’/RTpSAD‐RV), using 62 °C and 61 °C as the annealing temperature, respectively. RT‐PCR products were sequenced at the Macrogen facility. The expected sizes of the minigene full‐length (mgFL) transcripts were 627 nt (mgATM_41–44), 1,101 nt (mgBRCA1_13–19), 1,086 nt and 906 nt (mgPALB2_5–12).

To relatively quantify the expression of each transcript, semiquantitative fluorescent RT‐PCRs (26 cycles) were conducted in triplicate (experiments of c.6347 +2 T > A/G were replicated six times) as indicated above. FAM‐labeled products with LIZ‐600 or LIZ‐1200 size standards were run by Macrogen and analyzed with Peak Scanner V1.0 (Life Technologies). Only peak heights ≥50 RFU (Relative Fluorescence Units) were considered, and mean peak areas of each transcript and standard deviations were calculated.

### ACMG/AMP‐based tentative classification of +2 T > C variants

We classified variants according to ACMG‐AMP‐based guidelines transformed into a Bayesian classification framework [21, 32, 33]. For each individual variant under investigation, we evaluated the ACMG/AMP evidence according to ClinGen Hereditary Breast, Ovarian and Pancreatic Cancer Expert Panel Specifications to the ACMG/AMP Variant Interpretation (*ATM* v. 1.3.0, and *PALB2* v. 1.1.0), and ClinGen ENIGMA *BRCA1* and *BRCA2* Expert Panel Specifications to the ACMG/AMP Variant Interpretation (*BRCA1* v. 1.1.0). *ATM* and *BRCA1* specifications have been recently published [23, 27]. *PALB2* specifications have not been published yet, but are freely available online at the ClinGen repository (https://cspec.genome.network/cspec/ui/svi/doc/GN077). To transform complex minigene readouts (i.e. variants producing two or more transcripts) into a PVS1_(RNA) or BP7_(RNA) evidence of appropriate strength, we followed ClinGen SVI splicing subgroup recommendations [34]. In brief, we first applied pathogenic/benign evidence strength to individual transcripts according to the gene‐specific PVS1 decision trees; we next grouped transcripts supporting the same evidence strength, and we estimated the contribution of the pathogenic and benign signals (in this particular case, reference transcripts expressed by leaky variants) to overall expression. Finally, we produced an overall call by applying the following rules:

—For *ATM*, following a model that we have previously proposed [15], we applied BP7_S(RNA) to leaky variants expressing >30% of full‐length transcripts. We applied PVS1_(RNA) evidence (of variable strength) to nonleaky variants and to leaky variants expressing <13% of reference transcripts.
—For *BRCA1*, based on ENIGMA data [35], we applied BP7_S(RNA) to leaky variants expressing >30% of reference transcripts. We applied PVS1_(RNA) evidence (of variable strength) to nonleaky variants and to leaky variants expressing <15% of reference transcripts. This is based on the fact that *BRCA1* c.5277+2T > C (a leaky variant expressing 14% of reference transcript in the present study) scores loss‐of‐function in a ClinGen Variant Curation Expert Panel (VCEP) recognized functional assay [36].
—For *PALB2*, no experimental data or models establish a relationship between leakiness levels and pathogenicity or benignity. Therefore, following the operational 10% threshold proposed by the ClinGen SVI splicing subgroup recommendations [34], we applied PVS1_(RNA) to nonleaky variants and to leaky variants expressing <10% of reference transcripts.
—For leaky variants expressing reference transcripts within the ranges of 13%–30% (*ATM*), 15%–30% (*BRCA1*), and 10%–90% (*PALB2*), minigene readouts were considered clinically undecidable. Consequently, neither PVS1_(RNA) nor BP7_S(RNA) were applied.

## Results

We initially focused on three exons with strong GT splice sites (MES > 10.8) and SpliceAI DL < 0.8 (*ATM* exon 43, *BRCA1* exon 18, *PALB2* exon 7), where the +2T > C variants might generate functional noncanonical donor sites. Three minigenes with 19 exons of *ATM, BRCA1*, and *PALB2* were employed: mgATM_41–44, mgBRCA1_13–19, and the previously used mgPALB2_5–12 (Figure 1; supplementary material, Figure S1) [14]. These minigenes produced the expected mgFL and mimicked prevalent naturally‐occurring alternative splicing events according to SpliceVault (https://kidsneuro.shinyapps.io/splicevault/, accessed on 13 January 2025): *ATM* △(E43p49); *BRCA1* △(E13), △(E13_E14), and △(E14q11) (also identified as endogenous events in MCF‐7 cells; data not shown); and *PALB2* △(E7p10) (Figure 1; supplementary material, Table S4), suggesting that they are suitable for subsequent variant splicing assays [37].

### Splicing analysis of variants

**Figure 2:**
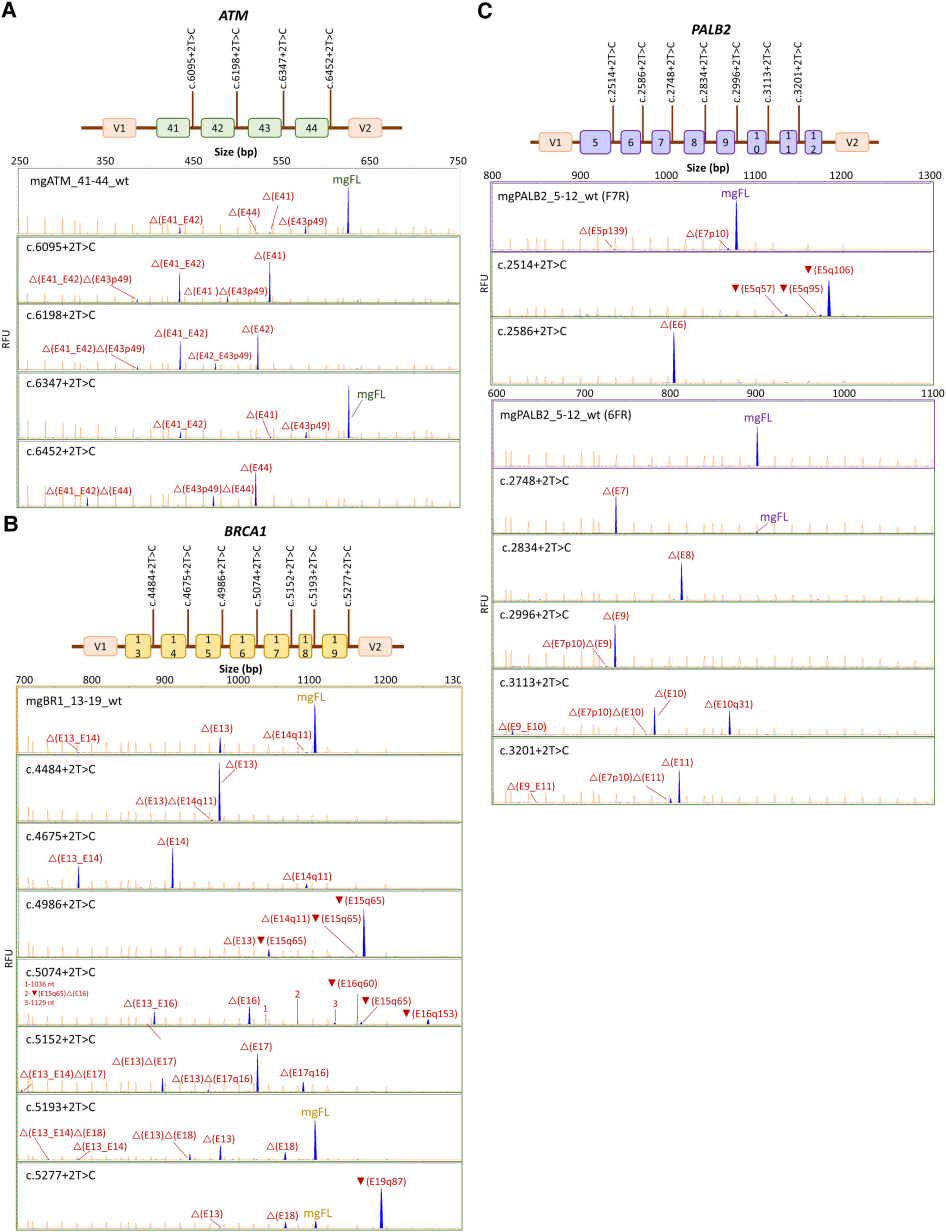
Fluorescent fragment analysis of minigene splicing assays of +2T > C variants of BC susceptibility genes. The maps of variants are shown on the top of each section. (A) mgATM_41–44. (B) mgBRCA1_13–19. (C) mgPALB2_5–12. FAM‐labeled RT‐PCR products (transcripts, blue peaks) were run with LIZ1200 (orange peaks) as size standard (mgFL, minigene full‐length transcript). RFU (y‐axis), relative fluorescence units.

**Figure 3:**
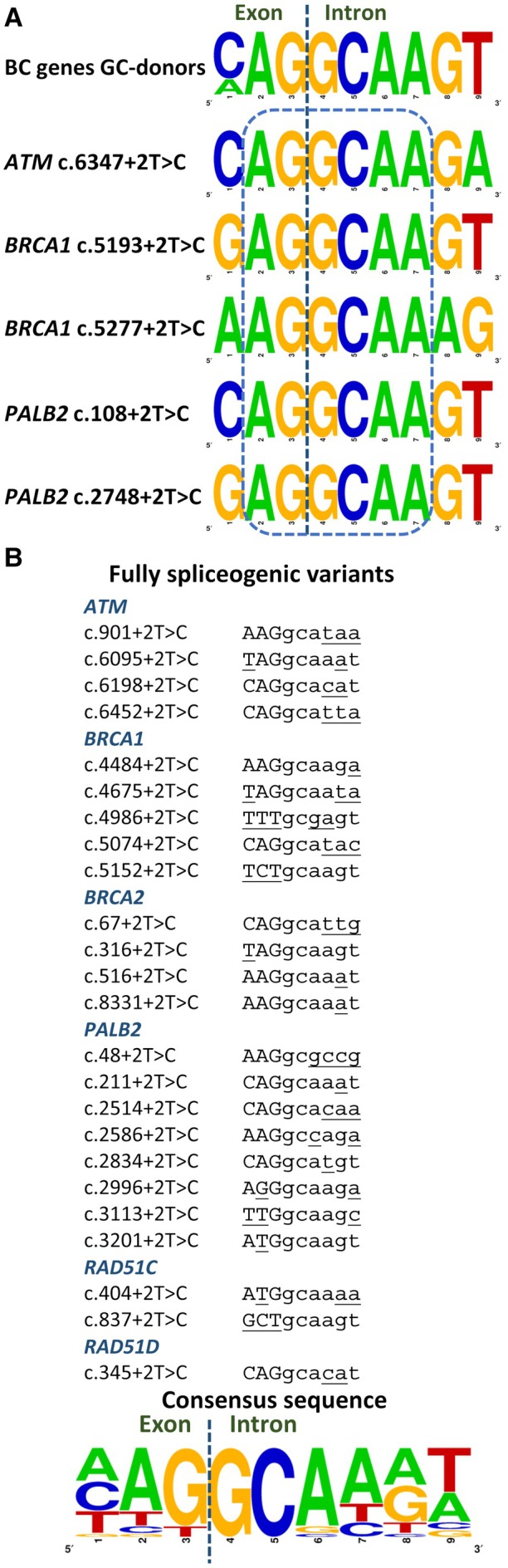
Sequence comparison of noncanonical GC donor sites. Pictograms were obtained using WebLogo (https://weblogo.berkeley.edu/logo.cgi). The size of each letter represents the nucleotide frequency at each position. (A) Sequence comparison of active GC‐donors of BC genes and active GC‐5'ss generated by +2T > C variants. (B) Sequences of fully spliceogenic +2T > C variants from this and previous studies and consensus sequence. Exonic and intronic sequences are shown in uppercase and lowercase, respectively. Nonconserved nucleotides of the 5'ss are underlined.

We proceeded to test all +2T > C variants from the three different minigenes, except for *PALB2* exon 12 with a natural GC‐donor site [14]. Hence, 18 variants were introduced into the minigenes: four in mgATM_41–44, seven in mgBRCA1_13–19, and seven in mgPALB2_5–12, which were assayed in MCF‐7 cells (Table 1, Figure 2). Interestingly, four variants generated functional GC‐donors recognized by the spliceosome, producing 3.8%–80.5% of the corresponding mgFL‐transcript: *ATM* c.6347+2T > C (80.5%); *BRCA1* c.5193+2T > C (60.7%), and c.5277+2T > C (13.8%); *PALB2* c.2748+2T > C (3.8%). Remarkably, variant *BRCA1* c.5193+2T > C was formerly classified as a functional allele by saturation genome editing that may be correlated with the expression of the wildtype transcript [36, 38]. Sequence comparison of the five +2T > C mgFL‐producing variants (including *PALB2* c.108+2T > C) revealed an invariable central core of six nucleotides (EXON|intron: AG|gcaa), whereas the 14 fully spliceogenic variants from this and previous minigene studies (Table 1; supplementary material, Table S1) showed greater variability in their 5'ss sequence (Figure 3).

**Table 1:** Splicing outcomes of +2 T variants of the breast cancer susceptibility genes.

Variant	Exon	Sequence wt^*^	Full‐length transcript	Transcripts
mgATM_11–17_wt			87.9% ± 0.2%	*In‐frame*: △(E11) (12.1% ± 0.2%)
c.1898+2T>C	12		‐	*PTC*: △(E12_E13p41) [3.6% ± 0.6%]; [△(E12)△(E15p19)] [1.5% ± 0.1%]; [△(E11)△(E13p41)△(E15p19)] [2.9% ± 0.1%] *In‐frame*: △(E12) [79% ± 0.6%]; △(E11_E12) [8.9% ± 0.6%]; [△(E12)△(E16)] [1.9% ± 0.2%] *Uncharacterized*: 975 nt [1% ± 0.2%]; 1,009 nt [1.2% ± 0.3%]
c.1898+2T>A	12		‐	*PTC*: [△(E12_E13)△(E15)] [2.3% ± 0.1%]; △(E12_E13p41) [4.5% ± 0.2%]; [△(E12)△(E15p19)] [1.9% ± 0.1%]; [△(E11)△(E13p41)△(E15p19)] [3.8%] *In‐frame*: △(E12) [66.9% ± 0.4%]; △(E11_E12) [10.5% ± 0.2%]; [△(E12)△(E16)] [2.5% ± 0.2%] *Uncharacterized*: 975 nt [1.8% ± 0.1%]; 1,001 nt [1.1% ± 0.1%]; 1,009 nt [2.4%]; 1,088 nt [1.1%]; 1,107 nt [1.2% ± 0.2%]
mgATM_41–44_wt			76.2% ± 0.1%	*PTC*: △(E43p49) [11.4%]; △(E41) [1.6% ± 0.1%] *In‐frame*: △(E41_E42) [9.8% ± 0.2%]; △(E44) [1%]
c.6095+2T>C	41	tAGGTAAaT	‐	*PTC*: △(E41) [75.9% + 1.5%]; △(E41_E43p49) [1.6% + 0.1%]; [△(E41)△(E43p49)] [2.9%] *In‐frame*: △(E41_E42) [19.6% ± 1.3%]
c.6198+2T>C	42	CAGGTAcaT	‐	*PTC*: △(E42) [62.6% ± 0.3%]; △(E42_E43p49) [8.4% ± 0.4%]; △(E41_E43p49) [2.1% ± 0.6%] *In‐frame*: △(E41_E42) [26.9% ± 0.5%]
c.6347+2T>C	43	CAGGTAAGa	80.5% ± 0.2%	*PTC*: △(E43p49) [9.1% ± 0.1%]; △(E41) [2.3%] *In‐frame*: △(E41_E42) [8.1% ± 0.3%]
c.6347+2T>A^†^	43		12.2% ± 3.4%	*PTC*: △(E43) [46.2% ± 12.3%]; △(E41_E43) [9.9% ± 1.3%]; ▼(I43^MG^) [9.2% ± 1.8%]; ▼(E43q97) [9.1% ± 1.8%]; △(E43q5) [5.4% ± 1.5%]; △(E42) [4.6% ± 1.9%]; [△(E41)△(E43)] [1.7% ± 0.2%]; *In‐frame*: △(E41_E42) [1.7% ± 0.5%]
c.6347+2T>G^†^	43		23.5% ± 6.7%	*PTC*: △(E43) [40.5% ± 13.2%]; ▼(I43^MG^) [11.2% ± 0.7%]; △(E41_E43) [10.3% ± 1.5%]; ▼(E43q97) [3%]; [△(E41)△(E43)] [1.6% ± 0.1%]; △(E41) [2% ± 0.6%]; △(E43p49) [2% ± 0.8%]; △(E42) [2% ± 0.9%] *In‐frame*: △(E41_E42) [3.9% ± 1.4%]
c.6452+2T>C	44	CAGGTAtta	‐	*PTC*: [△(E43p49)△(E44)] [19.5% ± 0.7%] *In‐frame*: △(E44) [66.3% ± 0.9%]; [△(E41_E42)△(E44)] [14.2% ± 0.2%]
mgBRCA1_13–19_wt			74.9% ± 1.2%	*PTC*: △(E13) [22.9% ± 1.2%]; △(E14q11) [1% ± 0.1%] *In‐frame*: △(E13_E14) [1.2% ± 0.1%]
c.4484+2T>C	13	AAGGTAAGa	‐	*PTC*: △(E13) [96.7% ± 1%]; [△(E13)△(E14q11)] [2% ± 0.3%] *Uncharacterized*: 620 nt [1.3% ± 1.1%]
c.4675+2T>C	14	tAGGTAAta	‐	*PTC*: △(E14) [61.4% ± 0.7%]; △(E14q11) [6%] *In‐frame*: △(E13_E14) [32.6% ± 0.7%]
c.4986+2T>C	15	tttGTGAGT	‐	*PTC*: ▼(E15q65) [81.6% ± 2.7%]; △(E13_E15) [1.9% ± 0.3%]; [△(E13)▼(E15q65)] [12.5% ± 1.9%]; [△(E14q11)▼(E15q65)] [1.2% ± 0.1%] *Uncharacterized*: 598 nt [2.8% ± 0.5%]
c.5074+2T>C	16	CAGGTAtac	‐	*PTC*: △(E16) [39.3% ± 1.1%]; [△(E13)△(E16)] [26.4% ± 1.2%]; [△(E13_E14)△(E16)] [6.1% ± 0.3%]; ▼(E15q65) [5.2% ± 0.3%];[▼(E15q65)△(E16)] [1.4% ± 0.1%];▼(E16q153) [12.5% ± 2.5%]; ▼(E16q60) [1.3% ± 0.1%] *Uncharacterized*: 504 nt [1.2% ± 0.2%]; 591 nt [2.1% ± 0.3%]; 1,036 nt [1.2% ± 0.4%]; 1,129 nt [3.3% ± 0.1%]
c.5152+2T>C	17	tctGTAAGT	‐	*PTC*: [△(E13)△(E17)] [22.9% ± 1.7%]; △(E17q16) [15.2% ± 1.4%]; [△(E13)△(E17q16)] [2.9% ± 0.4%]; [△(E13_E14)△(E17)] [3% ± 0.3%] *In‐frame*: △(E17) [56% ± 3.5%]
c.5193+2T>C	18	gAGGTAAGT	60.7% ± 3.5%	*PTC*: △(E13) [19.4% ± 1.6%]; △(E18) [9.6% ± 1.1%]; [△(E13_E14)△(E18)] [1.4% ± 0.1%]; [△(E13)△(E18)] [7.5% ± 0.6%] *In‐frame*: △(E13_E14) [1.4% ± 0.4%]
c.5193+2T>A	18		‐	*PTC*: △(E18) [79.6% ± 0.6%]; [△(E13)△(E18)] [20.4% ± 0.6%]
c.5193+2T>G	18		‐	*PTC*: △(E18) [83.5% ± 0.6%]; [△(E13)△(E18)] [13.8% ± 0.2%] *Uncharacterized*: 846 nt [1.7% ± 0.3%]; 1,051 nt [1% ± 0.5%]
c.5277+2T>C	19	AAGGTAAag	13.8% ± 0.3%	*PTC*: △(E18) [11.4% ± 0.4%]; △(E13) [1.2% ± 0.2%]; ▼(E19q87) [73.6% ± 1.2%]
c.5277+2T>A	19		‐	*PTC*: △(E18) [11.8% ± 0.3%]; △(E13) [1.7% ± 0.2%]; ▼(E19q87) [86.5% ± 0.2%]
c.5277+2T>G	19		‐	*PTC*: △(E18) [12.2% ± 0.6%]; ▼(E19q87) [87.8% ± 0.6%]
mgPALB2_1–3_wt			100%	
c.108+2T>A	2		‐	*In‐frame*: △(E2) [94.9%] *Uncharacterized*: 238 nt [1.9% ± 0.1%]; 287 nt [1.5%]; 345 nt [1.7% ± 0.2%]
c.108+2T>G	2		‐	*In‐frame*: △(E2) [95.8%] *Uncharacterized*:238 nt [1.2%]; 287 nt [1.6%]; 345 nt [1.4%]
mgPALB2_5–12_wt (F+7R)			91.6% ± 2.7%	*PTC*: △(E7p10) [3.9% ± 1.2%]; △(E5p139) [1.7% ± 0.5%] *Uncharacterized*:739 nt [2.8% ± 1%]
c.2514+2T>C	5	CAGGTAcaa	‐	*PTC*: ▼(E5q95) [2.8% ± 0.5%]; ▼(E5q106) [93.3% ± 0.9%]; ▼(E5q57) [3.9% ± 0.5%]
c.2586+2T>C	6	AAGGTcAGa	‐	*In‐frame*: △(E6) [100%]
mgPALB2_5–12_wt (6F+R)			100%	
c.2748+2T>C	7	gAGGTAAGT	3.8% ± 0.1%	*In‐frame*: △(E7) [96.2% ± 0.1%]
c.2748+2T>A	7		‐	*In‐frame*: △(E7) [97.7% ± 0.3%]; [△(E7)△(E9)] [2.3% ± 0.3%]
c.2748+2T>G	7		‐	*In‐frame*: △(E7) [97.5% ± 0.3%]; [△(E7)△(E9)] [2.5% ± 0.3%]
c.2834+2T>C	8	CAGGTAtGT	‐	*PTC*: △(E8) [100%]
c.2996+2T>C	9	AgGGTAAGa	‐	*PTC*: [△(E7p10)△(E9)] [2.7% ± 0.9%] *In‐frame*: △(E9) [97.3% ± 0.9]
c.3113+2T>C	10	ttGGTAAGc	‐	*PTC*: △(E10q31) [43.3% ± 0.2%]; [△(E7p10)△(E10)] [1.3% ± 0.1%] *In‐frame*: △(E9_E10) [6.8% ± 0.1%]; △(E10) [48.6% ± 0.3%]
c.3201+2T>C	11	AtGGTAAGT	‐	*PTC*: △(E11) [85.2% ± 0.5%]; [△(E7p10)△(E11)] [13.3% ± 0.4%]; [△(E9)△(E11)] [1.5%]

^†^ *ATM* c.6347+2T > A/G variants generated other uncharacterized transcripts (1,365 nt and 1,468 nt), that were not considered.

^*^ Changes in the consensus sequence are shown in lowercase.

The main variant‐induced splicing event was skipping of the corresponding exon (Table 1, Figure 2; supplementary material, Table S4). Notably, variant *BRCA1* c.5074+2T > C produced transcript ▼(E16q60) (1.3%) generated by an intronic cryptic weak GC‐donor (MES = 1.65).

### Analysis of other atypical donor sites

We previously showed that the *ATM* variant c.1898+2T > G created a functional GG‐donor (0.014% of human exons) [8], generating 13% of FL‐transcript in minigene assays [15]. With a view to examining the functionality of other +2T changes, we proceeded to evaluate: (1) +2T > A/G substitutions in exons whose +2T > C variants produced FL‐transcripts, and (2) T > C/A changes at *ATM* c.1898+2.

Remarkably, +2T > A/G variants of *ATM* exon 43, *BRCA1* exon 18, and *PALB2* exons 2 and 7 had MES scores around 3, similar to +2T > C variants of the same splice‐sites (supplementary material, Table S2). In this regard, two previous *ATM* and *PALB2* minigenes were incorporated into the study: mgATM_11–17 and mgPALB2_1–3 (Figure 1) [14, 15].

**Figure 4:**
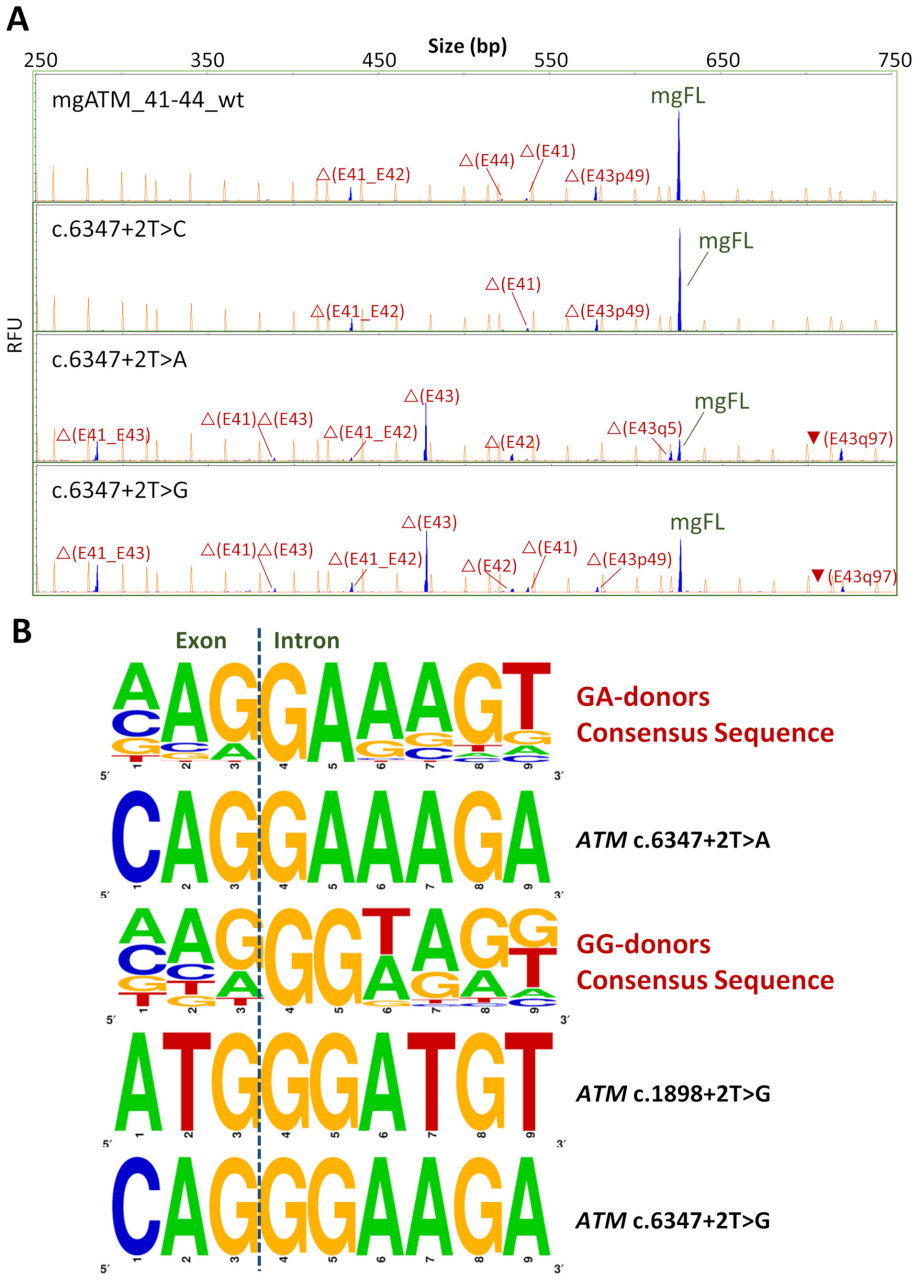
Splicing impact of +2T > A/G variants at *ATM* c.6347+2. (A) Splicing assay of variants c.6347+2T > C,A,G. FAM‐labeled RT‐PCR products (transcripts, blue peaks) were run with LIZ1200 (orange peaks) as size standard (mgFL, minigene full‐length transcript). RFU (*y*‐axis), relative fluorescence units. (B) Consensus sequence of exon–intron boundaries of 40 and 101 noncanonical human GA and GG‐splice sites [8], respectively, *versus* the sequence of the atypical functional GA/GG‐splicing donors created by *ATM* variants c.1898+2T > G and c.6347+2T > A/G.

Next, six +2T > A, five +2T > G, and one additional +2T > C variants were tested in MCF‐7 cells: *ATM* c.1898+2T > C/A, c.6347+2T > A/G, *BRCA1* c.5193+2T > A/G, c.5277+2T > A/G and *PALB2* c.108+2T > A/G, c.2748+2T > A/G. Interestingly, *ATM* variants c.6347+2T > A and +2T > G produced 12% and 24% mgFL‐transcript, respectively, indicating the creation of functional GA‐ and GG‐donors (Table 1, Figure 4; supplementary material, Figure S2). The rest of the variants induced 100% anomalous transcripts.

In summary, in this and previous studies we identified a total of eight +2T variants that expressed different levels of mgFL‐transcripts: *ATM* c.1898+2T > G (13% FL‐transcript) [15], c.6347+2T > C (81%), c.6347+2T > A (12%) and c.6347+2T > G (24%), *BRCA1* c.5193+2T > C (61%) and c.5277+2T > C (14%), and *PALB2* c.108+2T > C (86%) [14], and c.2748+2T > C (4%) (Table 1). Furthermore, four of these had not been predicted by SpliceAI (supplementary material, Table S2), assuming a Donor Loss score < 0.8 for putative functional donor sites.

### DeepCLIP analysis

To identify the putative splicing factors involved in GC‐donor recognition, we conducted a DeepCLIP analysis of the last 30 exonic nt of +2T > C leaky variants and natural GC‐exons of BC genes (supplementary material, Table S5). These predictions revealed at least six SR proteins and hnRNPs that could be involved in the recognition of these donor sites (DAZAP1, Tra2α, Tra2β, SRSF5, SRSF6, and hnRNP L). Similarly, GA and GG donor sites showed high binding capacities for the same SR factors, but additionally would also recruit SRSF1, SRSF2, SRSF7, and SRSF9 for exon recognition.

### Clinical interpretation

To evaluate the clinical impact of our study, we classified the 30 variants under investigation (Table 1) applying the pathogenic evidence strength proposed by ClinGen VCEPs in the corresponding PVS1 decision trees for *ATM* [27], *BRCA1* [23], and *PALB2* (https://cspec.genome.network/cspec/ui/svi/doc/GN077) or, alternatively, the PVS1_(RNA) /BP7_S(RNA) derived from our minigene readouts (supplementary material, Table S6). Overall, concordance was high, with identical classification for 28 of 30 variants (supplementary material, Figure S3), confirming several VCEP predictions on leakiness. For instance, minigene readouts confirmed that *ATM* c.6347+2T > C and *BRCA1* c.5193+2T > C are indeed leaky variants. Further, minigene readouts discarded leakiness for several *ATM* (*n* = 3), *BRCA1* (*n* = 5), and *PALB2* (*n* = 6) +2T > C variants, confirming VCEP predictions [23, 27].

Our study also showed that some VCEPs predictions on leakiness were inaccurate. For instance, minigene readouts demonstrated leakiness for up to four genetic variants (*ATM* c.6347+2T > A, *ATM* c.6347+2T > G, *BRCA1* c.5277+2T > C, and *PALB2* c.2748+2T > C) that were predicted as nonleaky by VCEPs. Although the level of FL‐transcripts produced by three of these leaky variants was too low (4%–14%) to influence variant classification, *ATM* c.6347+2T > G was an exception, expressing up to 24% of FL‐transcripts. This finding caused that the PVS1 code recommended by the *ATM*‐VCEP was substituted by PVS1_(RNA) ‘not applicable’ and, accordingly, the variant downgraded from LP to VUS (supplementary material, Figure S3 and Table S6).

Minigene readouts also had a major impact on *BRCA1* c.4675+2T > C classification, predicted nonleaky by the *BRCA1*‐VCEP, and confirmed by minigene assays. Yet the minigene readout revealed a significant contribution of the in‐frame transcript △(E13_E14) to the overall expression (33%). Since the functional impact of deleting 106 BRCA1 residues in the intrinsically disordered region linking the coiled‐coil and BRCT domains is far from obvious, the variant was downgraded to VUS. Interestingly, △(E13_E14) was not anticipated by the VCEP, but was rightly predicted by SpliceVault (3% of 300K‐RNA samples) [39].

## Discussion

At least 16 exceptions to the GT‐AG rule have been described [8], among which the combination GC‐AG is the most frequent (0.9%). Therefore, it is plausible that certain ±1,2 variants of the canonical splice sites can generate atypical sites that in turn could be recognized by the splicing machinery. In fact, several studies have reported +2 T > C variants that still allow recognition of their respective exons, generating different amounts of wildtype transcripts [14, 22, 38, 40].

So far, we have studied 30 +2T > C variants of the BC susceptibility genes, five of which were capable of generating functional GC donors and different levels of wildtype FL‐transcripts (4%–86% of the overall expression). In this regard, minigene assays combined with highly sensitive fluorescent fragment electrophoresis are particularly valuable for detecting minimal amounts of mgFL‐transcripts, as the isolated mutant allele is analyzed without the interference of the wildtype allele present in patient RNA samples.

The synthesis of fully functional transcripts by supposed pathogenic variants poses a challenge for genetic counseling of inherited diseases and suggests that it is necessary for a reevaluation of the established classification criteria of variants in disease susceptibility genes. Thus, according to the initially proposed ACMG/AMP classification scheme, all ±1,2 variants are considered likely pathogenic, regardless of the generation of full‐length or in‐frame transcripts, which would entail a radical change in their interpretation and, consequently, in the clinical management of patients. To underscore the magnitude of this challenge, we estimate that ~6,800 +2T > C variants, occurring within the roughly 5,000 disease‐causing genes catalogued in OMIM (https://omim.org/, accessed on 25 November 2024; averaging 8 donor sites/gene and 17% FL‐producing variants) [41], could potentially generate functional GC sites, thereby complicating molecular diagnostics.

All +2T > C leaky variants exhibit high sequence conservation (Figure 3A; 6 nt central core: AGgtaa), comparable to constitutive GC‐donors and those of the BC susceptibility genes *ATM* (exon 50), *BRCA2* (exon 17) and *PALB2* (exon 12) [17, 19, 20]. Therefore, as a general rule, recognition of the *de novo* GC‐donor sites can be associated with high sequence conservation in the other positions of the splice site consensus sequence and, consequently, with primary strong GT sites. Actually, 5'ss of +2 T > C leaky variants have an average MES score of 10.6 and an average SpliceAI Donor Loss (DL) score of 0.49 [22]. Analysis of all 163 5'ss from the eight main BC susceptibility genes revealed that, while 49 +2T > C variants conserve the central core AGgcaa, potentially creating active GC‐5'ss, applying an MES cutoff ≥10.6 would select only 19 variants (supplementary material, Table S2). Similarly, eight putative leaky +2T > C variants would be selected using a SpliceAI DL cutoff <0.49. Nevertheless, further experimental data are needed to establish the precise criteria for identifying leaky +2T variants.

Conversely, 25 +2T > C variants induced complete anomalous splicing patterns without any trace of the corresponding mgFL‐transcript (nonleaky variants; Table 1; supplementary material, Table S1). These totally spliceogenic variants typically present two or more additional nt‐changes with respect to the 5'ss consensus sequence (MAGgtragt), except for *BRCA1* c.4484+2T > C and *PALB2* c.3201+2T > C, which differ in only one nt (Table 1, Figure 3B). Therefore, fully spliceogenic +2T >C variants are associated with weaker GT‐donor sites. Indeed, the original GT‐5'ss of these fully spliceogenic +2T > C variants averaged an MES score of 8.0 [22]. *BRCA1* nonleaky variants c.4986+2T > C, c.5074+2T > C, and c.5152+2T > C were formerly identified as nonfunctional alleles [36, 38], lending further support for our results.

With regard to other atypical donor sites, *ATM* variants c.1898 +2 T > G [15], c.6347+2T > G, and c.6347+2T > A formed functional GG and GA donors, respectively. These represent very rare splice sites that have been described in only 32 (0.014%) and 14 human exons (0.006%), respectively [8]. Curiously, the GG and GA 5'ss are surprisingly much more frequent in the copepod (small crustacean) *Eurytemora affinis* that might have been recently incorporated into its genome, as other closely related species do not contain GA or GG 5'ss [42].

None of the three +2T > G/A variants reaches the mgFL expression of most +2T > C variants (up to 86% for *PALB2* c.108+2T > C) [14], suggesting that GC sites are more efficiently recognized by the U1 snRNA than GG/GA ones. Beyond the splice‐site strength of the original GT site, other elements, such as splicing factors (SR and hnRNP proteins), secondary structure, and the genomic environment, among others, may promote recognition of these GG/GA atypical sites. Actually, several SR proteins, including SRSF1, SRSF2, SRSF5, SRSF7, and Tra2β, are involved in GC recognition [11, 17, 18, 20]. DeepCLIP analysis revealed high binding scores for the splicing activators DAZAP1, Tra2α, Tra2β, SRSF5, and SRSF6, and for hnRNP L, among others (supplementary material, Table S5), suggesting a synergistic mechanism for GC‐exon recognition rather than a dedicated GC‐specific factor. Curiously, DAZAP1 and hnRNP L promote the inclusion of exons with weak 5'ss, like hnRNP H [43, 44, 45, 46].

### ACMP/AMP classification

On classifying experimentally confirmed leaky splice‐site variants, it is crucial to first establish thresholds for the minimum level of full‐length transcripts predicted to rescue activity (nonpathogenic) and the maximum level still associated with pathogenicity. Ideally, these thresholds should be supported by some kind of experimental and/or clinical data. For instance, it was previously estimated that ≥20%–30% of *BRCA1* expression is enough to confer tumor suppressor activity [35]. Here we show that *BRCA1* c.5277+2T > C, scoring nonfunctional in a clinically‐validated MAVE (multiplexed [functional] assays for variant effects) study [36], is a leaky variant expressing 13.8% mgFL‐transcript. The data indicate that ≤14% of *BRCA1* expression is likely a loss‐of‐function allele. In the absence of splicing data matched to clinical and/or functional data, the only alternative is to apply a very conservative operational threshold, such as the 10% as proposed by the ClinGen SVI, as we previously did for *PALB2* [14].

We performed two tentative classifications of 30 +2T > C/G/A variants (supplementary material, Table S6): an initial classification incorporating a predictive PVS1 evidence of variable strength, as recommended by the corresponding VCEPs in the gene‐specific PVS1 decision trees, and a final classification replacing the predictive PVS1 evidence with experimental PVS1_(RNA)/BP7_S(RNA) evidence derived from minigene readouts. Overall, the initial classification was largely confirmed by the minigene‐based approach (identical classification for 28 of the 30 variants investigated). In this regard, it is worth mentioning that gene‐specific PVS1 decision trees developed by ClinGen VCEPs rightly predicted leakiness for *ATM* c.6347+2T > C and *BRCA1* c.5193+2T > C. It is equally relevant to highlight that the minigene analysis uncovered leakiness effects and in‐frame readouts not previously anticipated by the VCEPs. Based on that, two variants (*ATM* c.6347+2T > G and *BRCA1* c.4675+2T > C) were downgraded from LP to VUS (supplementary material, Figure S3). In the case of *ATM* c.6347+2T > G, downgrading was based on leakiness (our minigene analysis revealed 24% reference transcripts). For *BRCA1* c.4675+2T > C, downgrading was based on the detection of a substantial proportion (33%) of in‐frame events △(E13_E14) in the intrinsically disordered BRCA1 region that, evaluated against the BRCA1‐specific PVS1 decision tree, equated to PVS1 with supporting strength only. The *BRCA1* PVS1 decision tree supports predictive PVS1 full‐strength evidence for this specific variant, probably based on *in silico* SpliceAI predictions (△(E14) and △(E14q11), supplementary material, Table S2), and previous data on *BRCA1* c.4675+1G > A producing △(E14) in a splicing reporter minigene [47], and △(E14)+△(E14q11) in RNA from one carrier [48]. Interestingly, another study on RNA from one c.4675+1G > A carrier detected △(E14)+△(E14q11)+△(E13_E14) [49]. SpliceVault searching of 300K‐samples for misspliced events at the *BRCA1* exon 14 donor shows that △(E14) and △(E13_E14) are Top‐1 and Top‐2 events (detected in 4.5% and 3% of samples), fully supporting in‐frame △(E13_E14) as a *bona fide BRCA1* c.4675+2T > C outcome.

It is likely that *ATM* c.6347+2T > G and *BRCA1* c.4675+2T > C complex readouts that combine transcripts supporting pathogenicity with transcripts supporting benignity are associated with some level of *ATM* and *BRCA1* reduced penetrance, albeit we are not aware of clinical and/or functional data supporting this claim.

In conclusion, a relevant proportion of +2T > C/G/A variants tested in this study (20%, 6/30) generates noncanonical GC, GG, and GA 5'ss that are recognized by the splicing machinery, expressing full‐length transcripts. Extrapolating these data to the ~181,000 annotated exons of the human genome, ~36,000 +2T > C/G/A variants would be capable of generating wildtype transcripts. Further, FL‐producing variants are derived from exons with strong GT‐splice sites, enabling their potential preselection. Altogether, these results indicate that +2 variants should not be considered strong evidence of pathogenicity according to current classification criteria, without any other supporting evidence. Further, variants may generate other functional atypical splice sites, such as a TG acceptor in the *CHEK2* gene [16], as there are many different combinations of noncanonical 3'ss and 5'ss, including those of the U11/U12 minor spliceosome [8, 50]. Finally, variant‐splicing assays provide crucial data for the characterization of these uncommon splicing events.

## Author contributions statement

EAV‐S was responsible for conceptualization of the study. Data curation was performed by IL‐B. Formal analysis was conducted by IL‐B, EB‐M, LS‐M, MdlH and EAV‐S. Funding acquisition was undertaken by EAV‐S and MdlH. The investigations were carried out by IL‐B, LS‐M, EB‐M, PP‐S, AG‐Á, AV‐P, MdlH and EAV‐S. Methodology was developed by IL‐B, LS‐M, EB‐M, AG‐Á, AV‐P, PP‐S, MdlH and EAV‐S. EAV‐S supervised the study. IL‐B and EAV‐S wrote the original draft of the article. IL‐B, MdlH and EAV‐S reviewed and edited the article. All authors read and approved the final version of the article.

## Supporting information


**Figure S1.** Insert sequences of minigenes mgATM_11–17, mgATM_41–44, mgBRCA1_13–19, mgPALB2_1–3 and mgPALB2_ex5–12 (provided as separate Word file)
**Figure S2**. Splicing assays of additional +2T > C/G/A changes
**Figure S3**. ClinGen/ACMG/AMP classification of 30 +2T > C/G/A variants
**Table S1**. Splicing outcomes of previously studied +2T > C/G variants by minigene assays
**Table S2**. Bioinformatics analysis of all +2T > C of the eight main breast cancer susceptibility genes (163 exons) and +2T > A/G variants tested in this study (provided as separate Word file)
**Table S3**. Cloning and mutagenesis primers
**Table S4**. Short descriptors and HGVS annotations of transcripts
**Table S5**. DeepCLIP analysis of GC‐ and GG‐donors (last 30 nucleotides of each exon): binding capacities of selected RNA Binding Proteins
**Table S6**. Clinical interpretation of 30 + 2 T variants (provided as separate Excel file)

## Data Availability

All sequencing and fragment analysis data are available at http://hdl.handle.net/10261/384046; http://doi.org/10.20350/DIGITALCSIC/17179.

## References

[1] Conti LD , Baralle M , Buratti E . Exon and intron definition in pre‐mRNA splicing. Wiley Interdiscip Rev RNA 2013; 4: 49–60. 23044818 10.1002/wrna.1140

[2] Busch A , Hertel KJ . Evolution of SR protein and hnRNP splicing regulatory factors. Wiley Interdiscip Rev RNA 2012; 3: 1–12. 21898828 10.1002/wrna.100 PMC3235224

[3] Rhine CL , Cygan KJ , Soemedi R , *et al*. Hereditary cancer genes are highly susceptible to splicing mutations. PLoS Genet 2018; 14: e1007231. 29505604 10.1371/journal.pgen.1007231 PMC5854443

[4] Blakes AJM , Wai HA , Davies I , *et al*. A systematic analysis of splicing variants identifies new diagnoses in the 100,000 genomes project. Genome Med 2022; 14: 79. 35883178 10.1186/s13073-022-01087-x PMC9327385

[5] Sanz DJ , Acedo A , Infante M , *et al*. A high proportion of DNA variants of BRCA1 and BRCA2 is associated with aberrant splicing in breast/ovarian cancer patients. Clin Cancer Res 2010; 16: 1957–1967. 20215541 10.1158/1078-0432.CCR-09-2564

[6] Pedrotti S , Cooper TA . In brief: (mis)splicing in disease. J Pathol 2014; 233: 1–3. 24615176 10.1002/path.4337 PMC4300095

[7] Mount SM . Genomic sequence, splicing, and gene annotation. Am J Hum Genet 2000; 67: 788–792. 10986039 10.1086/303098 PMC1287883

[8] Parada GE , Munita R , Cerda CA , *et al*. A comprehensive survey of noncanonical splice sites in the human transcriptome. Nucleic Acids Res 2014; 42: 10564–10578. 25123659 10.1093/nar/gku744 PMC4176328

[9] Dorling L , Carvalho S , Allen J , *et al*. Breast cancer risk genes — association analysis in more than 113,000 women. N Engl J Med 2021; 384: 428–439. 33471991 10.1056/NEJMoa1913948 PMC7611105

[10] Hu C , Hart SN , Gnanaolivu R , *et al*. A population‐based study of genes previously implicated in breast cancer. N Engl J Med 2021; 384: 440–451. 33471974 10.1056/NEJMoa2005936 PMC8127622

[11] Fraile‐Bethencourt E , Valenzuela‐Palomo A , Díez‐Gómez B , *et al*. Mis‐splicing in breast cancer: identification of pathogenic BRCA2 variants by systematic minigene assays. J Pathol 2019; 248: 409–420. 30883759 10.1002/path.5268

[12] Sanoguera‐Miralles L , Valenzuela‐Palomo A , Bueno‐Martínez E , *et al*. Comprehensive functional characterization and clinical interpretation of 20 splice‐site variants of the RAD51C gene. Cancers (Basel) 2020; 12: 3771. 33333735 10.3390/cancers12123771 PMC7765170

[13] Bueno‐Martínez E , Sanoguera‐Miralles L , Valenzuela‐Palomo A , *et al*. RAD51D aberrant splicing in breast cancer: identification of splicing regulatory elements and minigene‐based evaluation of 53 DNA variants. Cancers (Basel) 2021; 13: 2845. 34200360 10.3390/cancers13112845 PMC8201001

[14] Valenzuela‐Palomo A , Bueno‐Martínez E , Sanoguera‐Miralles L , *et al*. Splicing predictions, minigene analyses, and ACMG‐AMP clinical classification of 42 germline PALB2 splice‐site variants. J Pathol 2022; 256: 321–334. 34846068 10.1002/path.5839 PMC9306493

[15] Bueno‐Martínez E , Sanoguera‐Miralles L , Valenzuela‐Palomo A , *et al*. Minigene‐based splicing analysis and ACMG/AMP‐based tentative classification of 56 ATM variants. J Pathol 2022; 258: 83–101. 35716007 10.1002/path.5979 PMC9541484

[16] Sanoguera‐Miralles L , Valenzuela‐Palomo A , Bueno‐Martínez E , *et al*. Systematic minigene‐based splicing analysis and tentative clinical classification of 52 CHEK2 splice‐site variants. Clin Chem 2024; 70: 319–338. 37725924 10.1093/clinchem/hvad125

[17] Llinares‐Burguet I , Sanoguera‐Miralles L , Valenzuela‐Palomo A , *et al*. Splicing dysregulation of noncanonical GC‐5′ splice sites of breast cancer susceptibility genes ATM and PALB2. Cancers (Basel) 2024; 16: 3562. 39518003 10.3390/cancers16213562 PMC11545216

[18] Kralovicova J , Hwang G , Asplund a C , *et al*. Compensatory signals associated with the activation of human GC 5′ splice sites. Nucleic Acids Res 2011; 39: 7077–7091. 21609956 10.1093/nar/gkr306 PMC3167603

[19] Thanaraj TA , Clark F . Human GC‐AG alternative intron isoforms with weak donor sites show enhanced consensus at acceptor exon positions. Nucleic Acids Res 2001; 29: 2581–2593. 11410667 10.1093/nar/29.12.2581 PMC55748

[20] Fraile‐Bethencourt E , Díez‐Gómez B , Velásquez‐Zapata V , *et al*. Functional classification of DNA variants by hybrid minigenes: identification of 30 spliceogenic variants of BRCA2 exons 17 and 18. PLoS Genet 2017; 13: e1006691. 28339459 10.1371/journal.pgen.1006691 PMC5384790

[21] Richards S , Aziz N , Bale S , *et al*. Standards and guidelines for the interpretation of sequence variants: a joint consensus recommendation of the American College of Medical Genetics and Genomics and the Association for Molecular Pathology. Genet Med 2015; 17: 405–424. 25741868 10.1038/gim.2015.30 PMC4544753

[22] Lin JH , Tang XY , Boulling A , *et al*. First estimate of the scale of canonical 5′ splice site GT > GC variants capable of generating wild‐type transcripts. Hum Mutat 2019; 40: 1856–1873. 31131953 10.1002/humu.23821

[23] Parsons MT , de la Hoya M , Richardson ME , *et al*. Evidence‐based recommendations for gene‐specific ACMG/AMP variant classification from the ClinGen ENIGMA BRCA1 and BRCA2 variant curation expert panel. Am J Hum Genet 2024; 111: 2044–2058. 39142283 10.1016/j.ajhg.2024.07.013 PMC11393667

[24] Lopez‐Perolio I , Leman R , Behar R , *et al*. Alternative splicing and ACMG‐AMP‐2015‐based classification of PALB2 genetic variants: an ENIGMA report. J Med Genet 2019; 56: 453–460. 30890586 10.1136/jmedgenet-2018-105834 PMC6591742

[25] Yeo G , Burge CB . Maximum entropy modeling of short sequence motifs with applications to RNA splicing signals. J Comput Biol 2004; 11: 377–394. 15285897 10.1089/1066527041410418

[26] Jaganathan K , Panagiotopoulou SK , McRae JF , *et al*. Predicting splicing from primary sequence with deep learning. Cell 2019; 176: 535–548.e24. 30661751 10.1016/j.cell.2018.12.015

[27] Richardson ME , Holdren M , Brannan T , *et al*. Specifications of the ACMG/AMP variant curation guidelines for the analysis of germline ATM sequence variants. Am J Hum Genet 2024; 111: 2411–2426. 39317201 10.1016/j.ajhg.2024.08.022 PMC11568761

[28] Grønning AGB , Doktor TK , Larsen SJ , *et al*. DeepCLIP: predicting the effect of mutations on protein‐RNA binding with deep learning. Nucleic Acids Res 2020; 48: 7099–7118. 32558887 10.1093/nar/gkaa530 PMC7367176

[29] de Garibay GR , Acedo A , García‐Casado Z , *et al*. Capillary electrophoresis analysis of conventional splicing assays: IARC analytical and clinical classification of 31 BRCA2 genetic variants. Hum Mutat 2014; 35: 53–57. 24123850 10.1002/humu.22456

[30] Acedo A , Hernández‐Moro C , Curiel‐García Á , *et al*. Functional classification of BRCA2 DNA variants by splicing assays in a large minigene with 9 exons. Hum Mutat 2015; 36: 210–221. 25382762 10.1002/humu.22725 PMC4371643

[31] Valenzuela‐Palomo A , Sanoguera‐Miralles L , Bueno‐Martínez E , *et al*. Splicing analysis of 16 PALB2 ClinVar variants by minigene assays: identification of six likely pathogenic variants. Cancers (Basel) 2022; 14: 4541. 36139699 10.3390/cancers14184541 PMC9496955

[32] Tavtigian SV , Harrison SM , Boucher KM , *et al*. Fitting a naturally scaled point system to the ACMG/AMP variant classification guidelines. Hum Mutat 2020; 41: 1734–1737. 32720330 10.1002/humu.24088 PMC8011844

[33] Tavtigian SV , Greenblatt MS , Harrison SM , *et al*. Modeling the ACMG/AMP variant classification guidelines as a Bayesian classification framework. Genet Med 2018; 20: 1054–1060. 29300386 10.1038/gim.2017.210 PMC6336098

[34] Walker LC , Hoya M d l , Wiggins GAR , *et al*. Using the ACMG/AMP framework to capture evidence related to predicted and observed impact on splicing: recommendations from the ClinGen SVI splicing subgroup. Am J Hum Genet 2023; 110: 1046–1067. 37352859 10.1016/j.ajhg.2023.06.002 PMC10357475

[35] Hoya M d l , Soukarieh O , López‐Perolio I , *et al*. Combined genetic and splicing analysis of BRCA1 c.[594‐2A > C; 641A > G] highlights the relevance of naturally occurring in‐frame transcripts for developing disease gene variant classification algorithms. Hum Mol Genet 2016; 25: 2256–2268. 27008870 10.1093/hmg/ddw094 PMC5081057

[36] Findlay GM , Daza RM , Martin B , *et al*. Accurate classification of BRCA1 variants with saturation genome editing. Nature 2018; 562: 217–222. 30209399 10.1038/s41586-018-0461-z PMC6181777

[37] Romero A , García‐García F , López‐Perolio I , *et al*. BRCA1 alternative splicing landscape in breast tissue samples. BMC Cancer 2015; 15: 1–8. 25884417 10.1186/s12885-015-1145-9 PMC4393587

[38] Chen J‐M , Lin J‐H , Masson E , *et al*. The experimentally obtained functional impact assessments of 5’ splice site GT > GC variants differ markedly from those predicted. Curr Genomics 2020; 21: 56–66. 32655299 10.2174/1389202921666200210141701 PMC7324893

[39] Dawes R , Bournazos AM , Bryen SJ , *et al*. SpliceVault predicts the precise nature of variant‐associated mis‐splicing. Nat Genet 2023; 55: 324–332. 36747048 10.1038/s41588-022-01293-8 PMC9925382

[40] Lin JH , Wu H , Zou WB , *et al*. Splicing outcomes of 5′ splice site GT > GC variants that generate wild‐type transcripts differ significantly between full‐length and minigene splicing assays. Front Genet 2021; 12: 701652. 34422003 10.3389/fgene.2021.701652 PMC8375439

[41] Sakharkar MK , Chow VTK , Kangueane P . Distributions of exons and introns in the human genome. In Silico Biol 2004; 4: 387–393. 15217358

[42] Robertson HM . Noncanonical GA and GG 5’ intron donor splice sites are common in the copepod Eurytemora affinis. G3 (Bethesda) 2017; 7: 3967–3969. 29079681 10.1534/g3.117.300189 PMC5714493

[43] Choudhury R , Roy SG , Tsai YS , *et al*. The splicing activator DAZAP1 integrates splicing control into MEK/Erk‐regulated cell proliferation and migration. Nat Commun 2014; 5: 3078. 24452013 10.1038/ncomms4078 PMC4146490

[44] Motta‐Mena LB , Heyd F , Lynch KW . Context‐dependent regulatory mechanism of the splicing factor hnRNP L. Mol Cell 2010; 37: 223–234. 20122404 10.1016/j.molcel.2009.12.027 PMC2818868

[45] Xiao X , Wang Z , Jang M , *et al*. Splice site strength‐dependent activity and genetic buffering by poly‐G runs. Nat Struct Mol Biol 2009; 16: 1094–1100. 19749754 10.1038/nsmb.1661 PMC2766517

[46] Raponi M , Smith LD , Silipo M , *et al*. BRCA1 exon 11 a model of long exon splicing regulation. RNA Biol 2014; 11: 351–359. 24658338 10.4161/rna.28458 PMC4075520

[47] Steffensen AY , Dandanell M , Jønson L , *et al*. Functional characterization of BRCA1 gene variants by mini‐gene splicing assay. Eur J Hum Genet 2014; 22: 1362–1368. 24667779 10.1038/ejhg.2014.40 PMC4231409

[48] Whiley PJ , Guidugli L , Walker LC , *et al*. Splicing and multifactorial analysis of intronic BRCA1 and BRCA2 sequence variants identifies clinically significant splicing aberrations up to 12 nucleotides from the intron/exon boundary. Hum Mutat 2011; 32: 678–687. 21394826 10.1002/humu.21495 PMC4340479

[49] Machackova E , Foretova L , Lukesova M , *et al*. Spectrum and characterisation of BRCA1 and BRCA2 deleterious mutations in high‐risk Czech patients with breast and/or ovarian cancer. BMC Cancer 2008; 8: 140. 18489799 10.1186/1471-2407-8-140 PMC2413254

[50] Akinyi MV , Frilander MJ . At the intersection of major and minor spliceosomes: crosstalk mechanisms and their impact on gene expression. Front Genet 2021; 12: 700744. 34354740 10.3389/fgene.2021.700744 PMC8329584

[51] Fraile‐Bethencourt E , Valenzuela‐Palomo A , Díez‐Gómez B , *et al*. Minigene splicing assays identify 12 Spliceogenic variants of BRCA2 exons 14 and 15. Front Genet 2019; 10: 503. 31191615 10.3389/fgene.2019.00503 PMC6546720

[52] Sanoguera‐Miralles L , Bueno‐Martínez E , Valenzuela‐Palomo A , *et al*. Minigene splicing assays identify 20 Spliceogenic variants of the breast/ovarian cancer susceptibility gene RAD51C. Cancer 2022; 14: 2960. 10.3390/cancers14122960 PMC9221245 35740625

[53] Bryksin AV , Matsumura I . Overlap extension PCR cloning: a simple and reliable way to create recombinant plasmids. Biotechniques 2010; 48: 463–465. 20569222 10.2144/000113418 PMC3121328

[54] Brandão RD , Mensaert K , López‐Perolio I , *et al*. Targeted RNA‐seq successfully identifies normal and pathogenic splicing events in breast/ovarian cancer susceptibility and Lynch syndrome genes. Int J Cancer 2019; 145: 401–414. 30623411 10.1002/ijc.32114 PMC6635756

