# Supplementary material for: Generation of functional noncanonical donor splice sites by +2T variants in breast cancer susceptibility genes: impact on clinical interpretation
Source: J Pathol. 2025 Nov 13;268(2):150–63. doi: 10.1002/path.6497 (PMC12805630; doi:10.1002/path.6497)
Supplement: Supplementary file 1 — Figure S1. Insert sequences of minigenes mgATM_11–17, mgATM_41–44, mgBRCA1_13–19, mgPALB2_1–3 and mgPALB2_ex5–12 (provided as separate Word file) Figure S2. Splicing assays of additional +2T > C/G/A changes Figure S3. ClinGen/ACMG/AMP classification of 30 +2T > C/G/A variants Table S1. Splicing outcomes of previously studied +2T > C/G variants by minigene assays Table S2. Bioinformatics analysis of all +2T > C of the eight main breast cancer susceptibility genes (163 exons) and +2T > A/G variants tested in this study (provided as separate Word file) Table S3. Cloning and mutagenesis primers Table S4. Short descriptors and HGVS annotations of transcripts Table S5. DeepCLIP analysis of GC‐ and GG‐donors (last 30 nucleotides of each exon): binding capacities of selected RNA Binding Proteins Table S6. Clinical interpretation of 30 + 2 T variants (provided as separate Excel file) [file PATH-268-150-s001.zip › path6497-sup-0002-FiguresS2S3TablesS1S3-S5.docx]

**Generation of functional noncanonical donor splice sites by +2T variants in breast cancer susceptibility genes: impact on clinical interpretation**

I Llinares-Burguet *et al. J Pathol* <https://doi.org/10.1002/path.6497>

**Supplementary Figure S1 (provided as separate Word file)**

**Supplementary Figures S2–S3**

**Supplementary Tables S1, S3–S5**

**Supplementary Table S2 (provided as separate Word file)**

**Supplementary Table S6 (provided as separate Excel file)**

**Reference numbers refer to the main text list.**

**
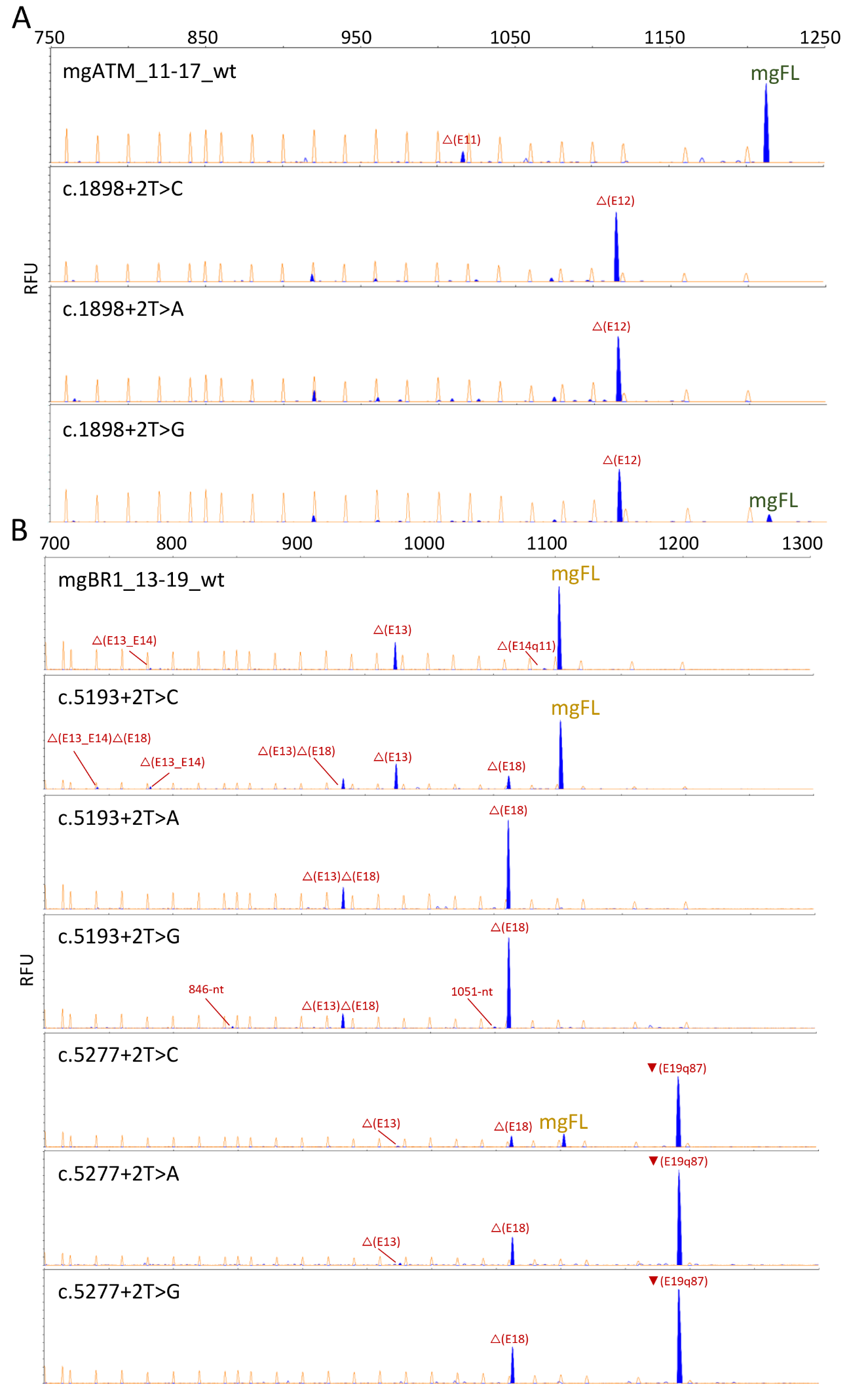
**

**
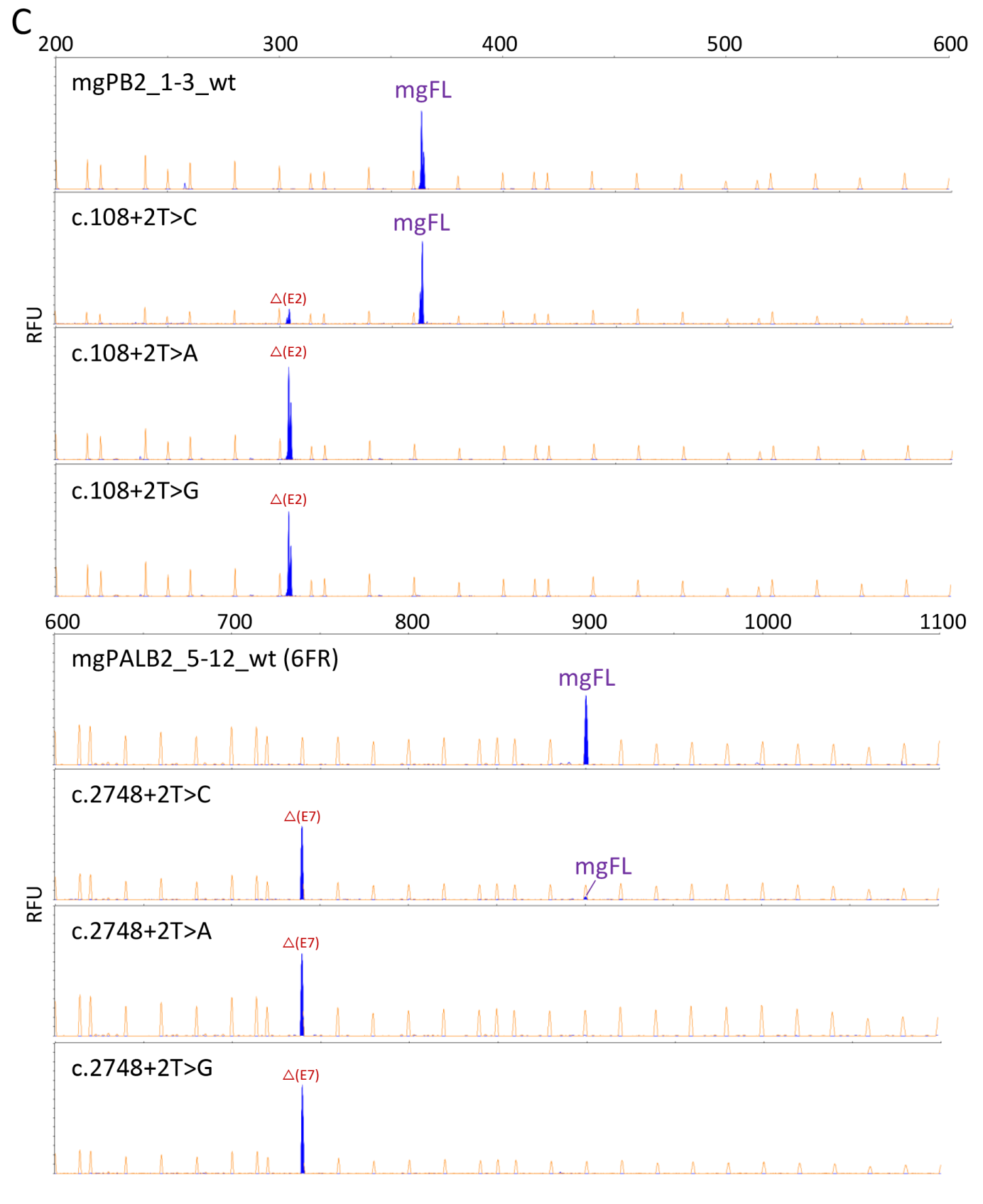
**

**
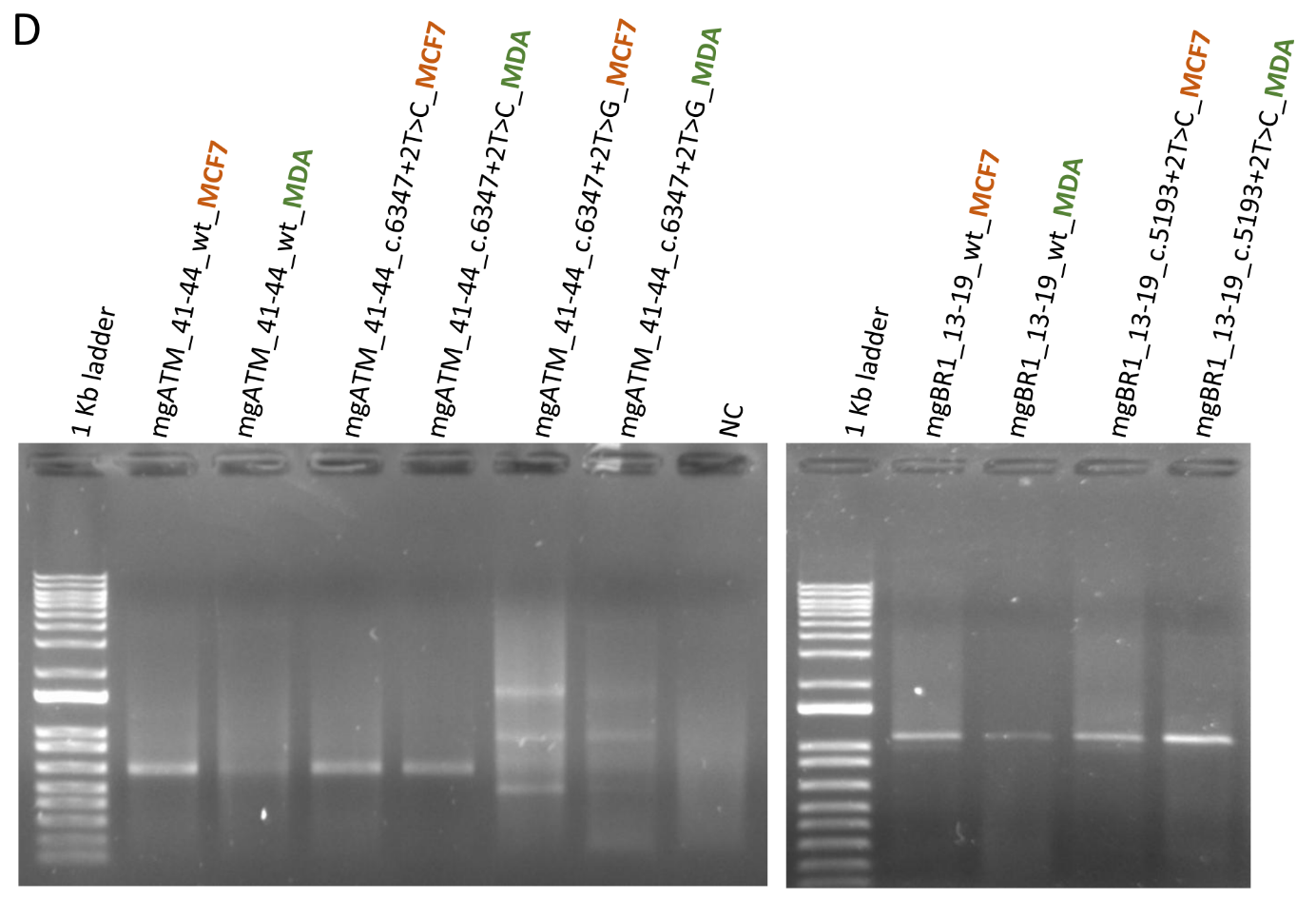
**

**Figure S2. Splicing assays of additional +2T > N changes. (**A) Analysis of +2T > C,A,G variants at ATM c.1898+2. For clarity, only the relevant peaks, △(E12) and mgFL, are shown. (B) +2T > A,C,G variants at BRCA1 c.5193+2 and c.5277+2. (C) +2T > A,C,G variants at PALB2 c.108+2 and c.2748+2. (D) Reproducibility of the minigene outcomes. Agarose gel (1%) electrophoresis of the splicing assays of ATM c.6347+2T > C,G variants, and BRCA1 c.5193+2T > C and the corresponding minigene wt in MCF-7 and MDA-MB-231. Cell growth and transfection were performed as described in the Materials and Methods section. The Gene Ruler 1 Kb Plus DNA Ladder (Thermo Scientific) was employed as DNA size standard. NC, negative control.

**
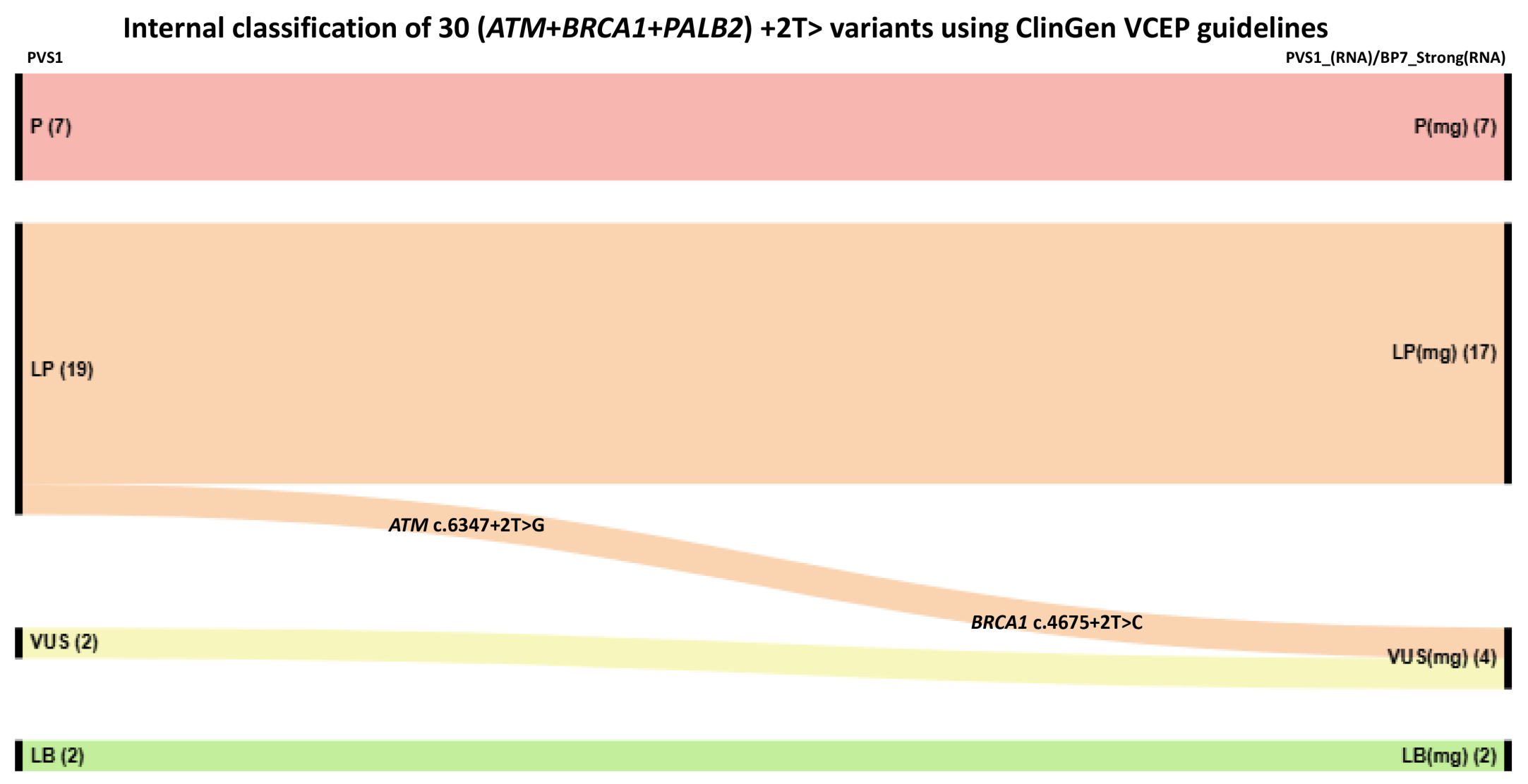
**

**Figure S3. ClinGen/ACMG/AMP classification of 30 +2T > N variants.** The Sankey plot summarizes the clinical impact of replacing PVS1 (as per ClinGen VCEP decision tree specifications) by splicing reporter minigene readout-based PVS1_(RNA)/BP7_Strong(RNA). Overall, both approaches are concordant for 28 variants. Yet, minigene readouts downgrade two variants (ATM c.6347+2T > G and BRCA1 c.4675+2T > C) from LP to VUS. P, pathogenic; LP, likely pathogenic; VUS, variant of uncertain significance; LB, likely benign; mg, splicing reporter minigene.

**Table S1.** Splicing outcomes of previously studied +2T>C/G variants by minigene assays.

| Gene^a^ | Variants | Exon | MES | WT Sequence | Full-Length Transcript | Transcripts |
| --- | --- | --- | --- | --- | --- | --- |
| *ATM*  [18] | c.901+2T>C | 7 | 5’ss (7.1→-0.7) | AAGgtataa | - | △(E7) [100%] |
|  | c.1898+2T>G | 12 | 5’ss (8.4→0.7) | ATGgtatgt | [13%] | △(E12) [87%] |
|  | c.7629+2T>G | 51 | 5'ss (8.6→1.0) | AATgtaagt | - | ∆(E51) (100%) |
| *BRCA2*  [11,14,51] | c.67+2T>C | 2 | 5’ss (8.4→0.6) | CAGgtattg | - | △(E2) [100%] |
|  | c.316+2T>C | 3 | 5’ss (9.7→1.9) | TAGgtaagt | - | △(E3) [100%] |
|  | c.516+2T>C | 6 | 5’ss (8.9→1.1) | AAGgtaaat | - | △(E6) [ ̴50%]  △(E5_E6) [ ̴50%] |
|  | c.7617+2T>G | 15 | 5’ss (9.8→2.2) | CAGgtatgt | - | Δ(E15) [100%] |
|  | c.8331+2T>C | 18 | 5’ss (8.9→1.1) | AAGgtaaat | - | △(E18) [87.1%]  △(E17q151_E18) [12.9%] |
| *CHEK2*  [19] | c.592+2T>G | 4 | 5’SS (8.5→0.7) | AAGgtaata | - | Δ(E4)▼(E4A38) [46.5%]; Δ(E4): 22.7%; Δ(E1q13)Δ(E4)▼(E4A38) [9.3%]; Δ(E4_E5) [6.7%]; Δ(E1q13)Δ(E4) [4.8%]; 952 nt: [10.0%] |
| *PALB2* [17,30] | c.48+2T>C | 1 | 5’ss (5.7→ -2) | AAGgtgccg | - | △(E1q17) [100%] |
|  | c.48+2T>G | 1 | 5’ss (5.7→ -1.9) | AAGgtgccg | - | △(E1q17) [100%] |
|  | c.108+2T>C | 2 | 5’ss (10.9→3.1) | CAGgtaagt | [85.5%] | △(E2) [14.5%] |
|  | c.211+2T>C | 3 | 5’ss (8.8→1.0) | CAGgtaaat | - | △(E3) [48.1%]  △(E3q48b) [51.9%] |
| *RAD51C* [15,52] | c.404+2T>C | 2 | 5’ss (4.8→-3) | ATGgtaaaa | - | ▼(E2q27) [77.2%]; △(E2) [16.7%]; △(E2q175) [4.7%]; 657 nt [1.4%] |
|  | c.837+2T>C | 5 | 5’ss (8.6→0.8) | GCTgtaagt | - | △(E4_E5) [2.2%]; △(E5) [89.3%];  972 nt [8.5%] |
| *RAD51D* [16] | c.345+2T>C | 4 | 5’ss (7.8→0.04) | CAGgtacat | - | △(E4) [49.6%]; △(E4_E5) [26.4%]; △(E3_E5) [24.0%] |

^a^ Reference numbers refer to the main text list.

**Table S3**. Cloning and mutagenesis primers.

| Cloning primers mgATM_41–44 and mgBRCA1_13–19 | | | | |
| --- | --- | --- | --- | --- |
| Cloning | | Primer sequences (5’ 🡪3’) | | |
| *ATM* Ex41–42 F | | CGGCCGCTCTAGAACTAGTGGATCCCCCGGTGAACTGTATTTCAGAACTGTATTTCAGAA | | |
| *ATM* Ex41–42 R | | TATCGATAAGCTTGATATCGAATTCCTGCATCTCCCTGAATTTTTTCTCTTTTTTTTGCA | | |
| *ATM* Ex43 F | | TGCAAAAAAAAGAGAAAAAATTCAGGGAGATGATATTTTGGGATTTTAAATGATATTGTG | | |
| *ATM* Ex43 R | | TATCGATAAGCTTGATATCGAATTCCTGCAAAACAACTCCTGTTATATTCATAGAAGAGA | | |
| *ATM* Ex44 F | | TCTCTTCTATGAATATAACAGGAGTTGTTTTGAGTAATTTCCTTTTTTTCTGCTTAAAGA | | |
| *ATM* Ex44 R | | GACGGTATCGATAAGCTTGATATCGAATTCGTAAAGAATGAACCTGGAATCCCAGAAACA | | |
| *BRCA1* Ex13 F | | CACACACGGCCGCACACATGATCATATTGTCTGAGGGGATTTGGG | | |
| *BRCA1* Ex13 R | | CACACAGGATCCAGAAGTCTACCATCAGTTTCC | | |
| *BRCA1* Ex14 F | | CACACAGGATCCCCCCATGTTATATGTCAACCC | | |
| *BRCA1* Ex14 R | | CACACAGAATTCCAAGACTCCCTCATCCTCAAA | | |
| *BRCA1* Ex15 F | | CACACAGAATTCATACTATTCCTATGACTAAACCT | | |
| *BRCA1* Ex15 R | | CACACACTCGAGCGAAAGTCCTATATCATACCCA | | |
| *BRCA1* Ex16 F | | CACACACTCGAGCTTCAGGTGTCTTAGAATTTTT | | |
| *BRCA1* Ex16 R | | CACACAGGTACCCACACACAAAGCTTATCCATGCTATGCTCAACAAA | | |
| *BRCA1* Ex17–18 F | | CACACAAAGCTTTAGAAGATGGGTGTTGAGAAGA | | |
| *BRCA1* Ex17–18R | | CACACAGGTACCCACACAATCGATGCCTGCATAATTCTTGATGATCC | | |
| *BRCA1* Ex19 F | | CACACAAAGCTTCTGCCTGGCAATTCTGAATGC | | |
| *BRCA1* Ex19 R | | CACACACTCGAGACCCCAGTGAAGTGAAAAGAG | | |
| *BRCA1* delivs13 F | | TAACATAAGCTACAACATGAGAAATCCCATGTTATATGTCAACCCTGACA | | |
| *BRCA1* delivs13 R | | TGTCAGGGTTGACATATAACATGGGATTTCTCATGTTGTAGCTTATGTTA | | |
| *BRCA1* delivs16 F | | AATAAAAAAATTAGCTGGGTGTGGTCCCTAGACTTCCAAATATCCATACC | | |
| *BRCA1* delivs16 R | | GGTATGGATATTTGGAAGTCTAGGGACCACACCCAGCTAATTTTTTTATT | | |
| *BRCA1* delivs18 F | | GCCTGGCAATTCTGAATGCCTTAAATGAATGCCTTAAATATGACGTGTCT | | |
| *BRCA1* delivs18 R | | AGACACGTCATATTTAAGGCATTCATTTAAGGCATTCAGAATTGCCAGGC | | |
| Mutagenesis primers for variants | | | | |
| Variant^a^ | Gene | | Exon | Primer sequences (5’ 🡪3’)^b^ |
| c.1898+2T > M | *ATM* | | E12 | TCCAAAGCGTGCCAGAATGGMATGTTATCTAATAATGCTCT |
|  |  |  |  | AGAGCATTATTAGATAACATKCCATTCTGGCACGCTTTGGA |
| c.6095+2T > C | *ATM* | | E41 | TGTTACAACCCATTACTAGGCAAATTGCATTTTTCTAAACA |
|  |  |  |  | TGTTTAGAAAAATGCAATTTGCCTAGTAATGGGTTGTAACA |
| c.6198+2T > C | *ATM* | | E42 | CCAGGCAGGAATCATTCAGGCACATTTTTTCCCAGATTTGG |
|  |  |  |  | CCAAATCTGGGAAAAAATGTGCCTGAATGATTCCTGCCTGG |
| c.6347+2T > C | *ATM* | | E43 | ACCATTGCACTTCCGTCAGGCAAGAAATTTGACTTGATTTT |
|  |  |  |  | AAAATCAAGTCAAATTTCTTGCCTGACGGAAGTGCAATGGT |
| c.6347+2T > R | *ATM* | | E43 | ACCATTGCACTTCCGTCAGGRAAGAAATTTGACTTGATTTT |
|  |  |  |  | AAAATCAAGTCAAATTTCTTYCCTGACGGAAGTGCAATGGT |
| c.6452+2T > C | *ATM* | | E44 | AAAGTCTCAAATATGCCAGGCATTATGAAAAGACAAAGTTA |
|  |  |  |  | TAACTTTGTCTTTTCATAATGCCTGGCATATTTGAGACTTT |
| c.4484+T > C | *BRCA1* | | E13 | AAGAACCAGGAGTGGAAAGGCAAGAAACATCAATGTAAAGA |
|  |  |  |  | TCTTTACATTGATGTTTCTTGCCTTTCCACTCCTGGTTCTT |
| c.4675+2T > C | *BRCA1* | | E14 | TTGCCAAGGCAAGATCTAGGCAATATTTCATCTGCTGTATT |
|  |  |  |  | AATACAGCAGATGAAATATTGCCTAGATCTTGCCTTGGCAA |
| c.4986+2T > C | *BRCA1* | | E15 | CCTGACCCCAGAAGAATTTGCGAGTGTATCCATATGTATCT |
|  |  |  |  | AGATACATATGGATACACTCGCAAATTCTTCTGGGGTCAGG |
| c.5074+2T > C | *BRCA1* | | E16 | CATGTTGTTATGAAAACAGGCATACCAAGAACCTTTACAGA |
|  |  |  |  | TCTGTAAAGGTTCTTGGTATGCCTGTTTTCATAACAACATG |
| c.5152+2T > C | *BRCA1* | | E17 | TGGGTAGTTAGCTATTTCTGCAAGTATAATACTATTTCTCC |
|  |  |  |  | GGAGAAATAGTATTATACTTGCAGAAATAGCTAACTACCCA |
| c.5193+2T > C | *BRCA1* | | E18 | AAGAAAAATGCTGAATGAGGCAAGTACTTGATGTTACAAAC |
|  |  |  |  | GTTTGTAACATCAAGTACTTGCCTCATTCAGCATTTTTCTT |
| c.5193+2T > R | *BRCA1* | | E18 | AAGAAAAATGCTGAATGAGGRAAGTACTTGATGTTACAAAC |
|  |  |  |  | GTTTGTAACATCAAGTACTTYCCTCATTCAGCATTTTTCTT |
| c.5277+2T > C | *BRCA1* | | E19 | AGAATCCCAGGACAGAAAGGCAAAGCTCCCTCCCTCAAGTT |
|  |  |  |  | AACTTGAGGGAGGGAGCTTTGCCTTTCTGTCCTGGGATTCT |
| c.5277+2T > R | *BRCA1* | | E19 | AGAATCCCAGGACAGAAAGGRAAAGCTCCCTCCCTCAAGTT |
|  |  |  |  | AACTTGAGGGAGGGAGCTTTYCCTTTCTGTCCTGGGATTCT |
| c.108+2T > R | *PALB2* | | E2 | GACACTAGCCCGCCTTCAGGRAAGTGAATCGTATTCTCAAA |
|  |  |  |  | TTTGAGAATACGATTCACTTYCCTGAAGGCGGGCTAGTGTC |
| c.2514+2T > C | *PALB2* | | E5 | TAAACATTCCGTCGAACAGGCACAATCCATTTCCTCTGTGA |
|  |  |  |  | TCACAGAGGAAATGGATTGTGCCTGTTCGACGGAATGTTTA |
| c.2586+2T > C | *PALB2* | | E6 | ATTGGTTTCAGAGTTAAAGGCCAGAAGAATATTCTCTTCCA |
|  |  |  |  | TGGAAGAGAATATTCTTCTGGCCTTTAACTCTGAAACCAAT |
| c.2748+2T > C | *PALB2* | | E7 | TACCTGGCACTTCGCAGAGGCAAGTGGGAATCTCGAGCTGA |
|  |  |  |  | TCAGCTCGAGATTCCCACTTGCCTCTGCGAAGTGCCAGGTA |
| c.2748+2T > R | *PALB2* | | E7 | TACCTGGCACTTCGCAGAGGRAAGTGGGAATCTCGAGCTGA |
|  |  |  |  | TCAGCTCGAGATTCCCACTTYCCTCTGCGAAGTGCCAGGTA |
| c.2834+2T > C | *PALB2* | | E8 | TGGAAATCAGAGAGATCAGGCATGTAATTCCCAAGGAGTGA |
|  |  |  |  | TCACTCCTTGGGAATTACATGCCTGATCTCTCTGATTTCCA |
| c.2996+2T > C | *PALB2* | | E9 | CGTTTGCAGAAGATGGAGGGCAAGAAAAGCATTGATTGATT |
|  |  |  |  | AATCAATCAATGCTTTTCTTGCCCTCCATCTTCTGCAAACG |
| c.3113+2T > C | *PALB2* | | E10 | TGAACAACATTGTTATTTGGCAAGCTTTCCCTCTAGGTCCT |
|  |  |  |  | AGGACCTAGAGGGAAAGCTTGCCAAATAACAATGTTGTTCA |
| c.3201+2T > C | *PALB2* | | E11 | CAAAGCCTATTCTGAAATGGCAAGTAATGACTGGCTGGGAC |
|  |  |  |  | GTCCCAGCCAGTCATTACTTGCCATTTCAGAATAGGCTTTG |

^a^ Ambiguous code: M = A, C and R = A, G

^b^ Variants are underlined within the primer sequence.

**Table S4.** Short descriptors and HGVS annotations of transcripts.

| Transcripts^a^ | HGVS-RNA | HGVS-protein | Diagrams of the splicing events |
| --- | --- | --- | --- |
| mgFL  mgATM_11–17 | | | 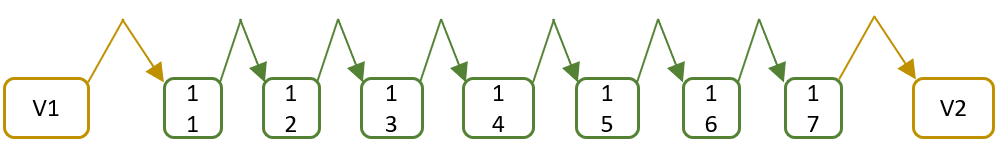 |
| △(E11) | r.1608_1802del | p.(Pro537_Ser601del) | 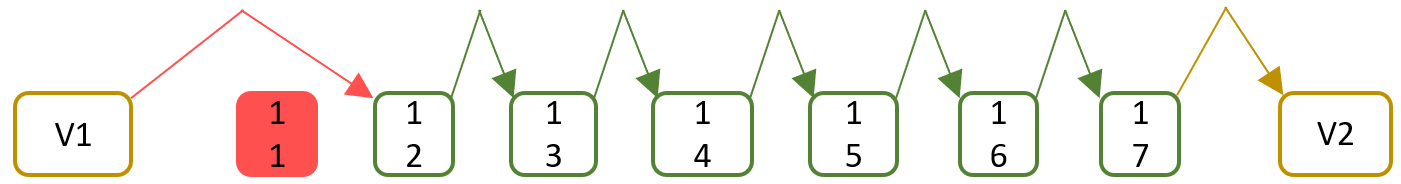 |
| △(E12) | r.1803_1898del | p.(Asn602_Cys633del) | 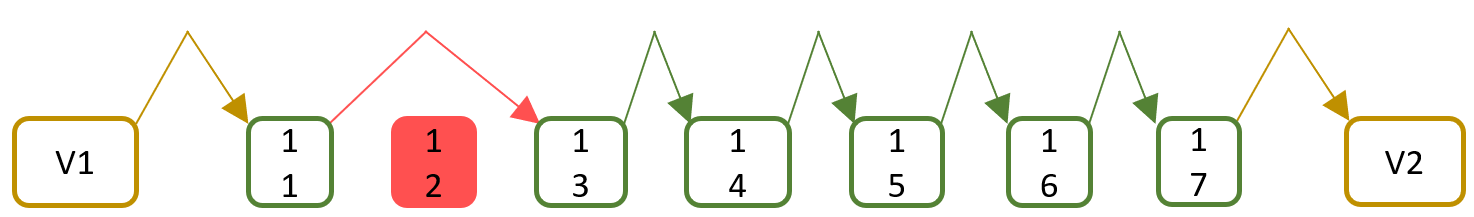 |
| △(E11_E12) | r.1608_1898del | p.(Pro537_Cys633del) | 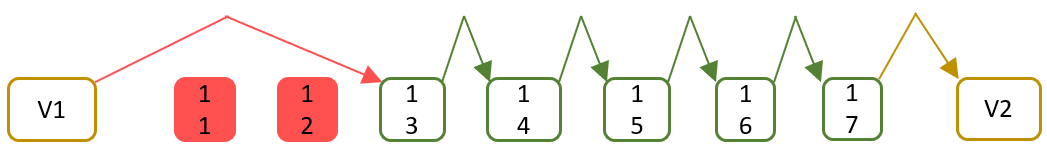 |
| △(E12_E13p41) | r.1803_1942del  PTC-NMD | p.(Asn602Argfs*10) | 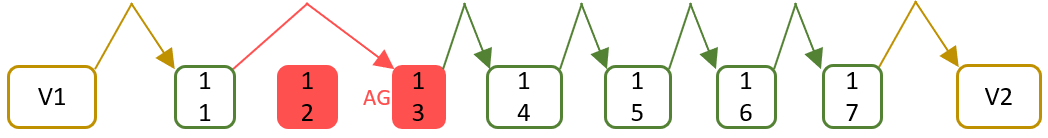 |
| △(E12)△(E15p19) | r.[1803_1898del,2251_2269del]  PTC-NMD | p.([Asn602_Cys633del, Ser751Glufs*20]) | 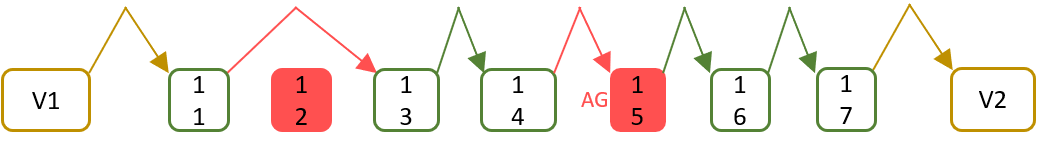 |
| △(E11)△(E13p41)△(E15p19) | r.[1608_1802del,1899_1939del,  2251_2269del] | p.([Pro537_Ser601del, p.Cys633*]) | 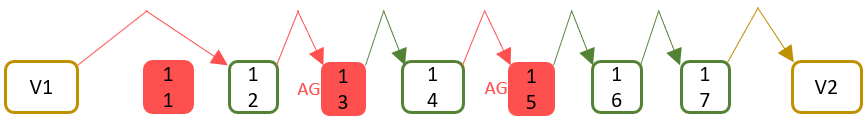 |
| △(E12_E13)△(E15) | r.[1803_2124del,2251_2376del]  PTC-NMD | p.(Ser601Argfs*27) | 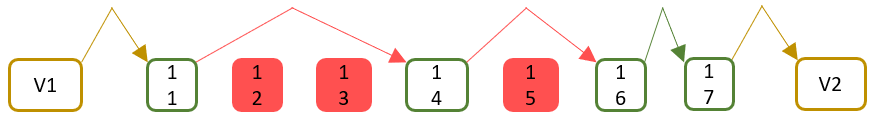 |
| △(E12)△(E16) | r.[1803_1898del,2377_2466del] | p.([Asn602_Cys633del, Lys796_Leu822del]) | 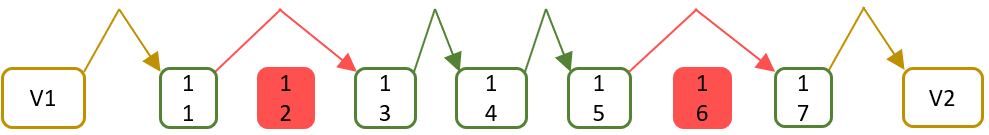 |
| mgFL  mgATM_41-44 | | | 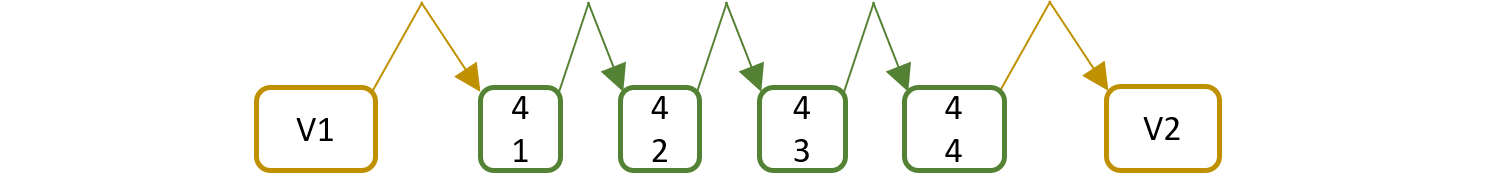 |
| △(E41) | r.6007_6095del  PTC-NMD | p.(Asp2003Thrfs*5) | 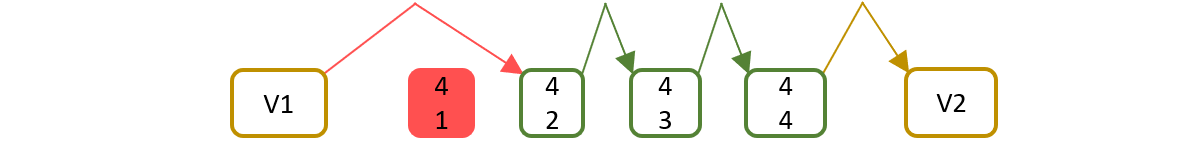 |
| △(E41)△(E43p49) | r.[6007_6095del, 6199_6247del]  PTC-NMD | p.(Asp2003Thrfs*5) | 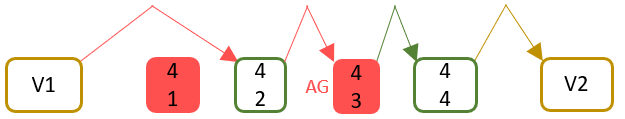 |
| △(E42) | r.6096_6198del  PTC-NMD | p.(Leu2033Profs*15) | 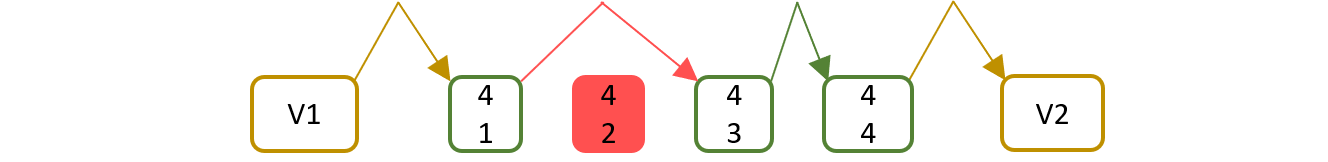 |
| △(E42_E43p49) | r.6096_6247del  PTC-NMD | p.(Leu2033Ilefs*4) | 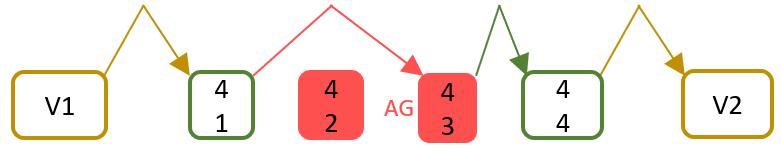 |
| △(E41_E42) | r.6007_6198del | p.(Asp2003_Gln2066del) | 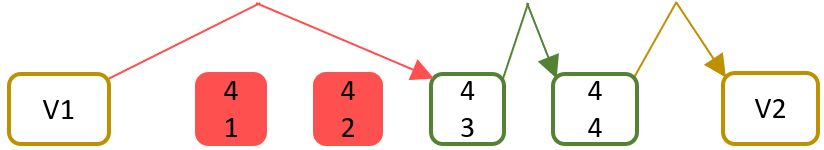 |
| △(E43p49) | r.6199_6247del  PTC-NMD | p.(Ala2067Aspfs*13) | 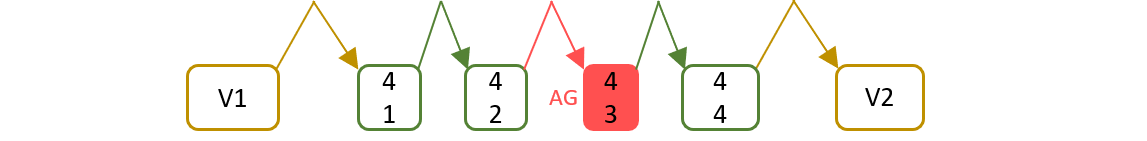 |
| △(E41_E43p49) | r.6007_6247del  PTC-NMD | p.(Leu2004Trpfs*12) | 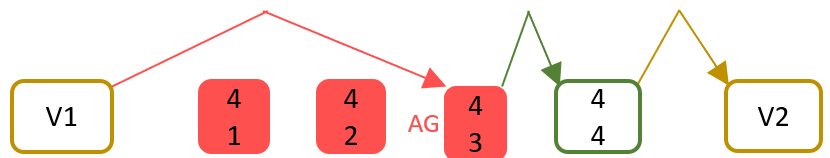 |
| △(E43) | r.6199_6347del  PTC-NMD | p.(Ala2067Glnfs*10) | 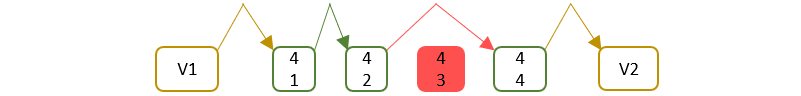 |
| △(E41)△(E43) | r.[6007_6095del, 6199_6347del]  PTC-NMD | p.(Asp2003Thrfs*5,) | 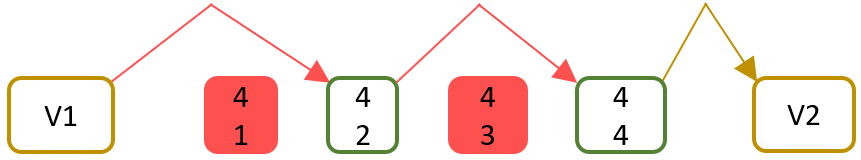 |
| △(E41_E43) | r.6007_6347del  PTC-NMD | p.(Asp2003Glnfs*10) | 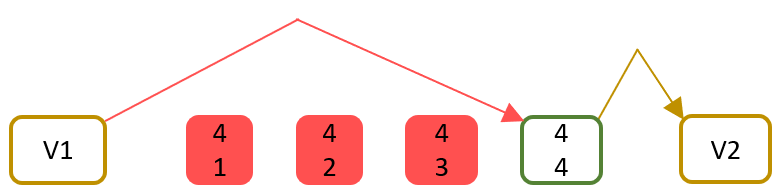 |
| △(E43q5) | r.6343_6347del  PTC-NMD | p.(Val2115Glnfs*10) | 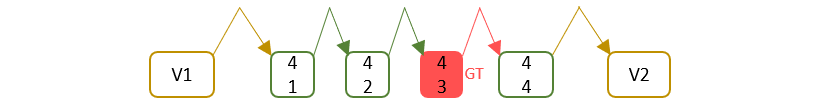 |
| ▼(E43q97 ^mg^)^b^ | r.6348_6349ins[gr;6348+3_6348+97^mg^]  PTC-NMD | p.(Ser2116delinsArgTer) | 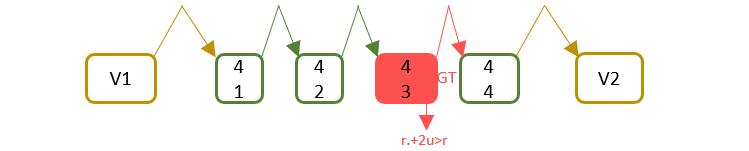 |
| ▼(I43^mg^)^c^ | r.6348_6349ins[gr;6348+3_6349-1]^mg^  PTC-NMD | p.(Ser2116delinsArgTer) | 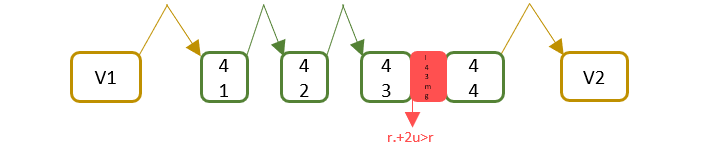 |
| △(E44) | r.6348_6452del | p.(Ser2116_Ala2150del) | 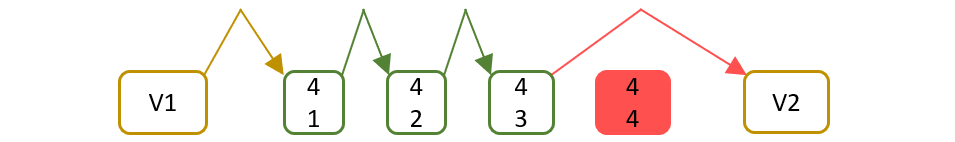 |
| △(E43p49)△(E44) | r.[ 6199_6247del, 6348_6452del]  PTC-NMD | p.(Ala2067Aspfs*13) | 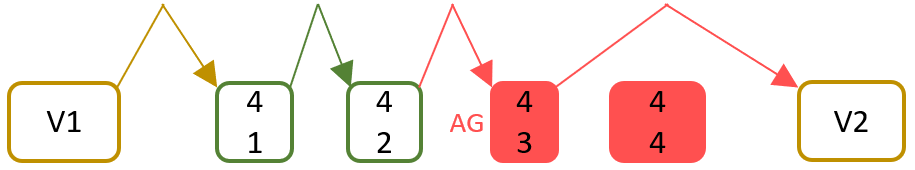 |
| △(E41_E42)△(E44) | r.[6007_6198del, 6348_6452del] | p.([Asp2003_Gln2096del, Ser2116_Ala2150del]) | 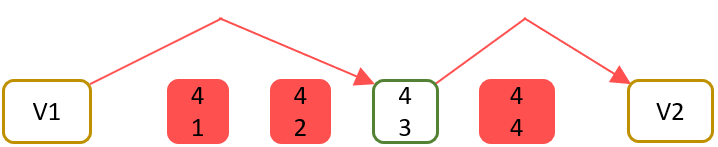 |
| mgFL  mgBRCA1_13-19 | | | 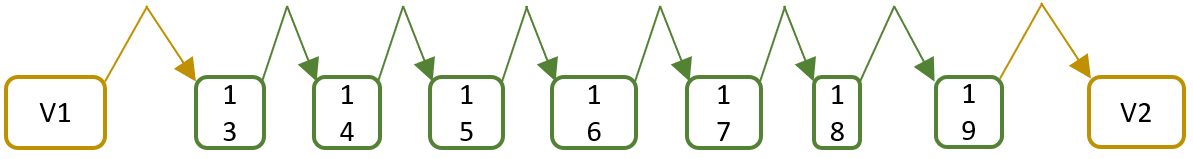 |
| △(E13) | r.4358_4484del  PTC-NMD | p.(Ala1453Glyfs*10) | 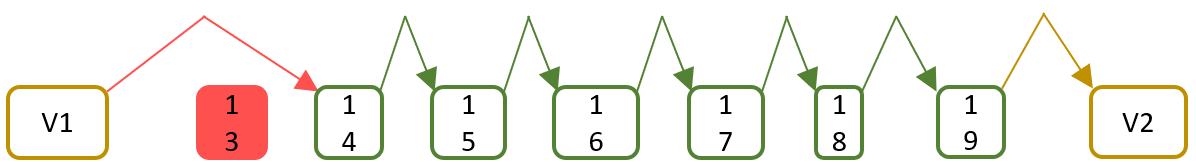 |
| △(E14) | r.4485_4675del  PTC-NMD | p.(Ser1496Glyfs*14) | 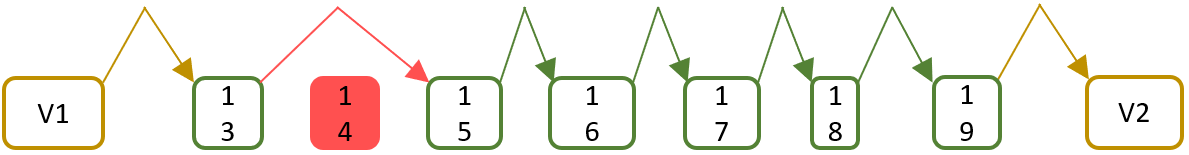 |
| △(E14q11)  [Generated by a cryptic GC-donor (MES: 2.81)] | r.4665_4675del  PTC-NMD | p.(Gln1556Glyfs*14) | 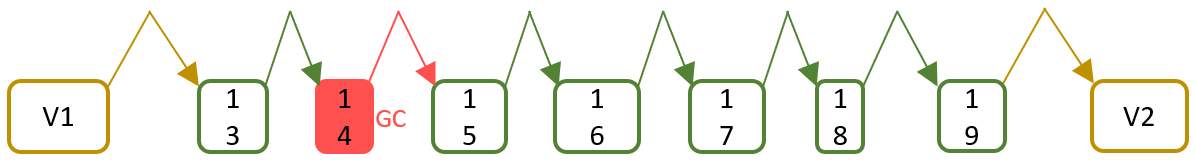 |
| △(E13_E14) | r.4358_4675del | p.(Ala1453_Leu1558del) | 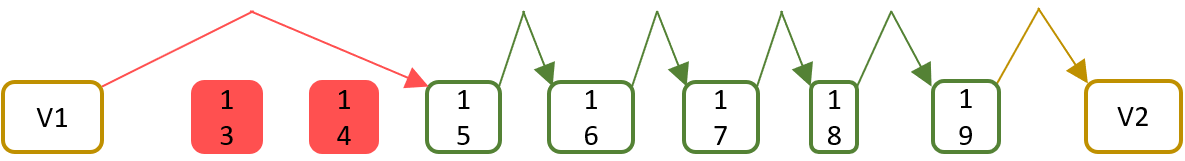 |
| △(E13)△(E14q11) | r.[4358_4484del, 4665_4675del]  PTC-NMD | p.(Ala1453Glyfs*10) | 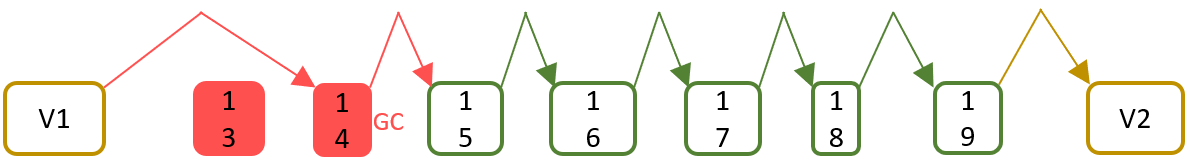 |
| ▼(E15q65) | r.4986_4987ins[gc;4986+3_4986+65]  PTC-NMD | p.(Phe1662_Met1663ins*14) | 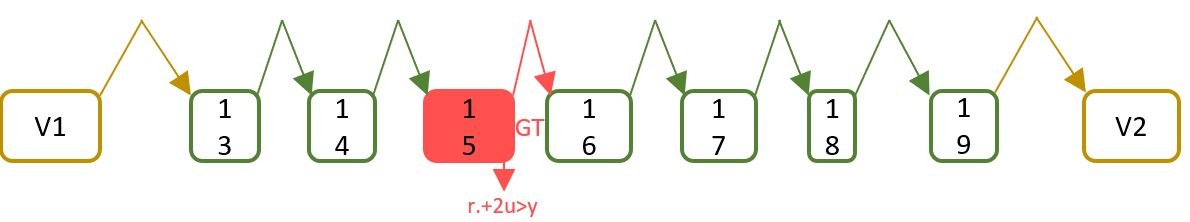 |
| △(E13)▼(E15q65) | r.[4358_4484del,  4986_4987ins(gc;4986+3_4986+65)]  PTC-NMD | p.(Ala1453Glyfs*10) | 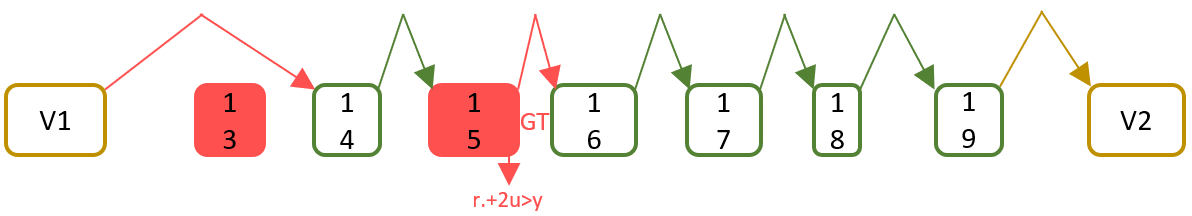 |
| △(E14q11)▼(E15q65) | r.[4665_4675del, 4986_4987ins(gc;4986+3_4986+65)]  PTC-NMD | p.(Gln1556Glyfs*14) | 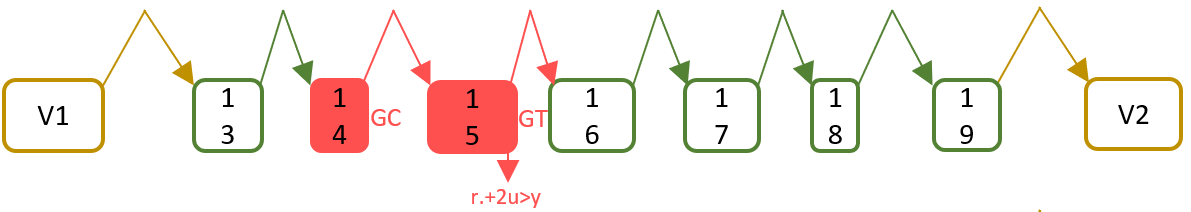 |
| △(E13_E15) | r.4358_4986del  PTC-NMD | p.(Ala1453Aspfs*16) | 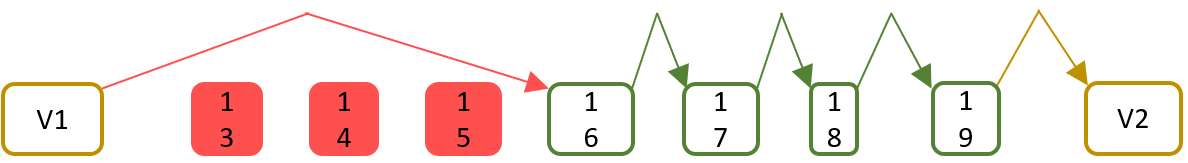 |
| △(E16) | r.4987_5074del  PTC-NMD | p.(Val1665Serfs*8) | 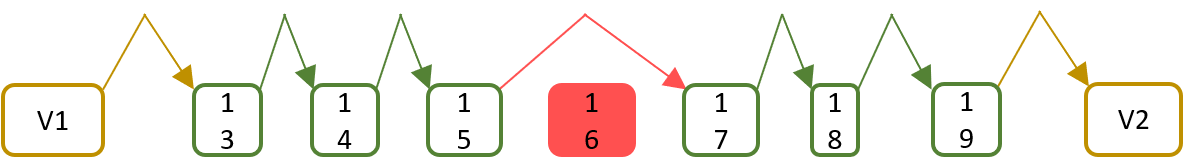 |
| ▼(E16q60)  [generated by an intronic cryptic weak GC-donor (MES = 1.65)] | r.5074_5075ins[gc;5074+3_5074+60]  PTC-NMD | p.(Asp1692Glyfs*15) | 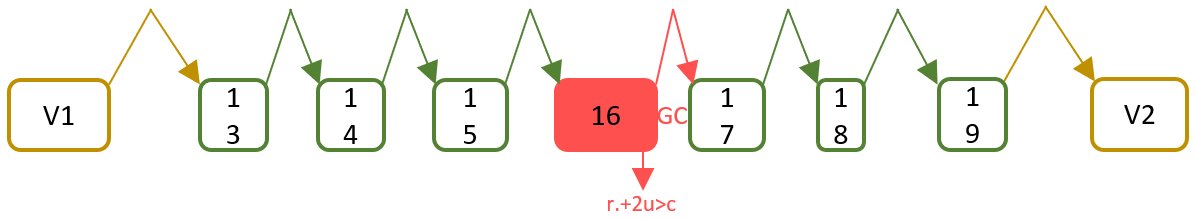 |
| ▼(E16q153) | r.5074_5075ins[gc;5074+3_5074+153]  PTC-NMD | p.(Asp1692Glyfs*15) | 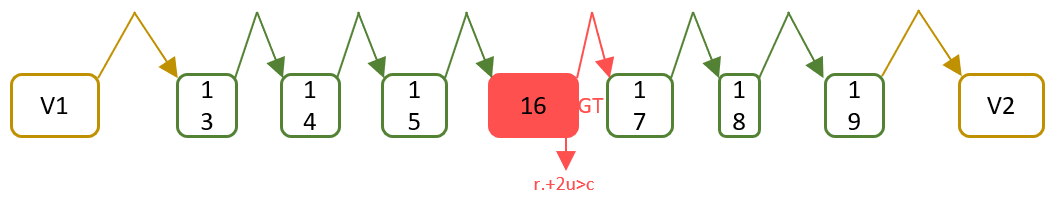 |
| △(E13)△(E16) | r.[4358_4484del, 4987_5074del]  PTC-NMD | p.(Ala1453Glyfs*10) | 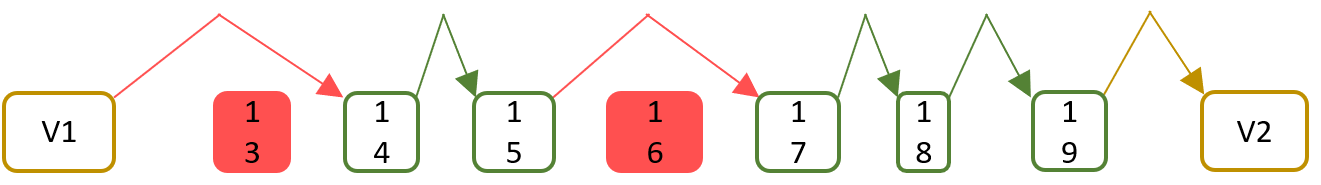 |
| △(E13_E14)△(E16) | r.[4358_4675del, 4987_5074del]  PTC-NMD | p.([Ala1453_Leu1558del, Val1665Serfs*8]) | 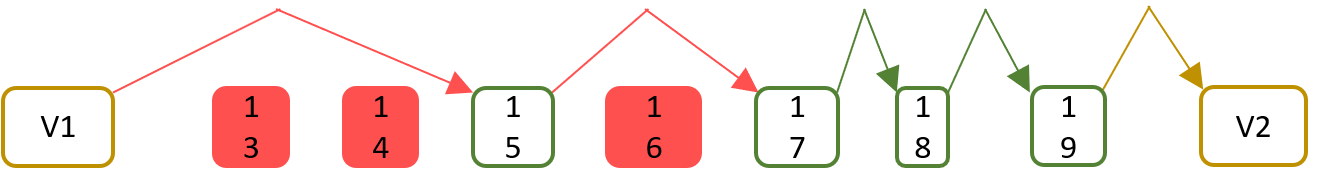 |
| ▼(E15q65)△(E16) | r.[4986_4987insgu;4986+3_4986+65, 4987_5074del]  PTC-NMD | p.(Phe1662_Met1663ins*14) | 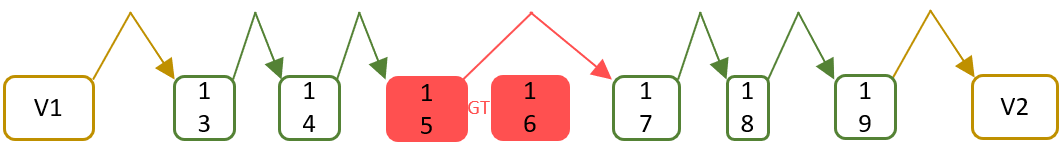 |
| △(E17) | r.5075_5152del | p.(Asp1692_Trp1718delinsGly) | 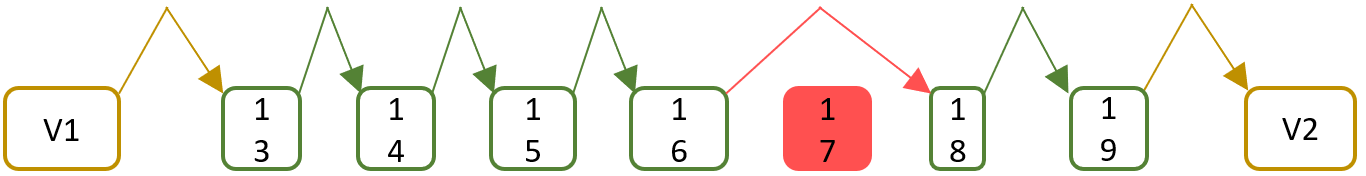 |
| △(E17q16) | r.5137_5152del  PTC-NMD | p.(Val1713Glyfs*2) | 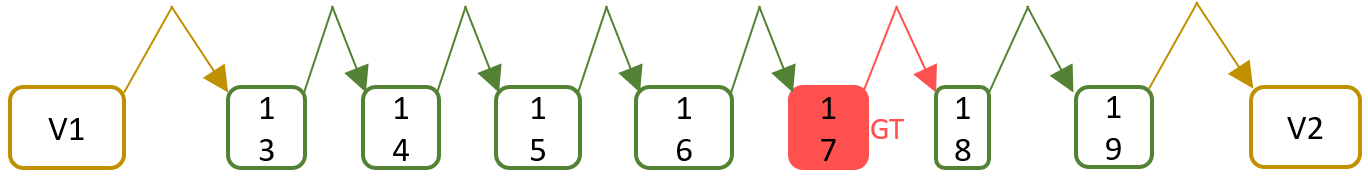 |
| △(E13)△(E17q16) | r.[4358_4484del, 5137_5152del]  PTC-NMD | p.(Ala1453Glyfs*10) | 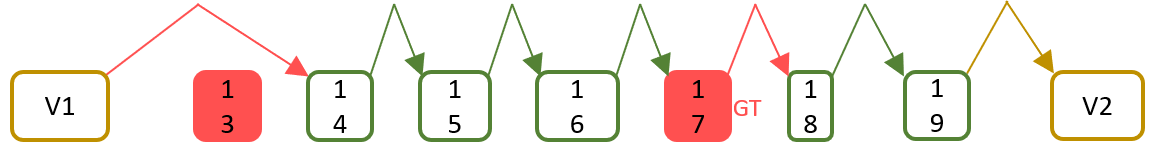 |
| △(E13)△(E17) | r.[4358_4484del, 5075_5152del]  PTC-NMD | p.(Ala1453Glyfs*10) | 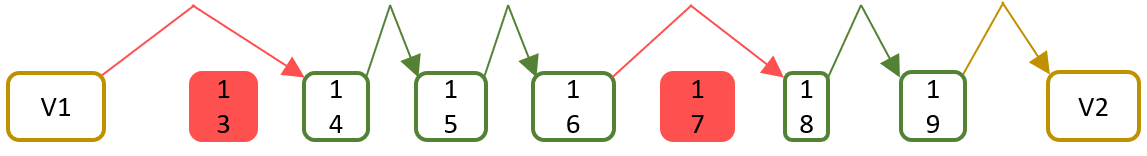 |
| △(E13_E14)△(E17) | r.[4358_4675del, 5075_5152del] | p.([Ala1453_Leu1558del, Asp1692_Trp1718delinsGly]) | 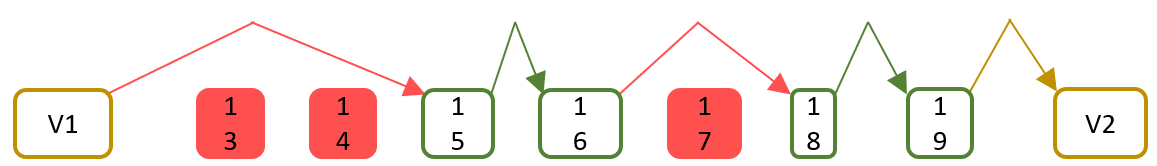 |
| △(E18) | r.5153_5193del  PTC-NMD | p.(Trp1718Serfs*2) | 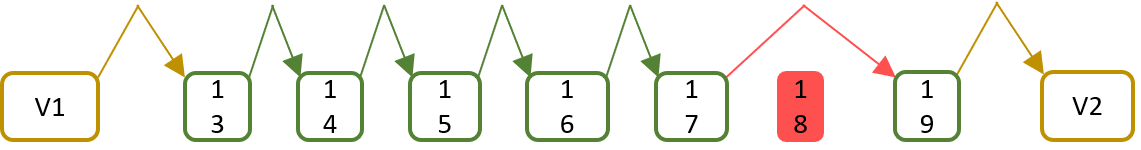 |
| △(E13_E14)△(E18) | r.[4358_4675del, 5153_5193del]  PTC-NMD | p.([Ala1453_Leu1558del, Trp1718Serfs*2]) | 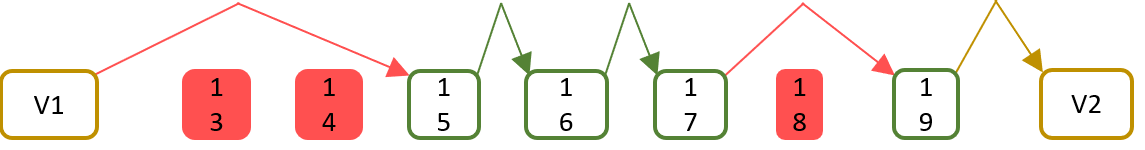 |
| △(E13)△(E18) | r.[4358_4484del, 5153_5193del]  PTC-NMD | p.(Ala1453Glyfs*10) | 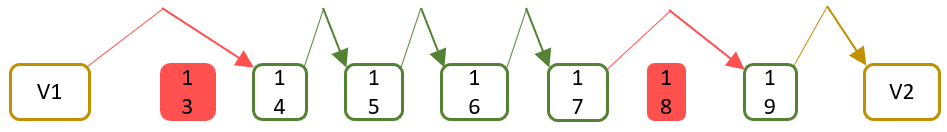 |
| ▼(E19q87) | r.5277_5278ins[gc;5277+3_5277+87]  PTC-NMD | p.(Lys1759_Ile1760ins*8) | 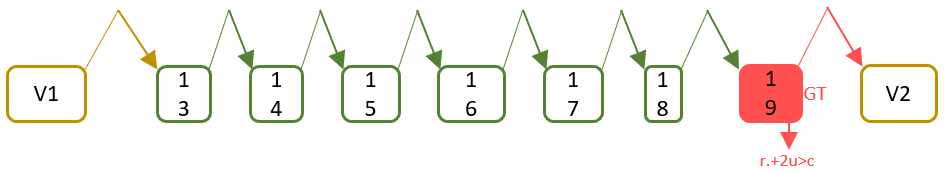 |
| mgFL  mgPALB2_1–3 | | | 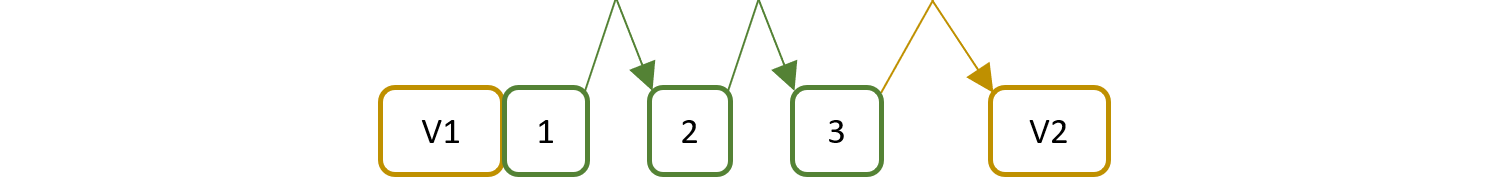 |
| △(E2) | r.49_108del | p.(Leu17_Gln36del) | 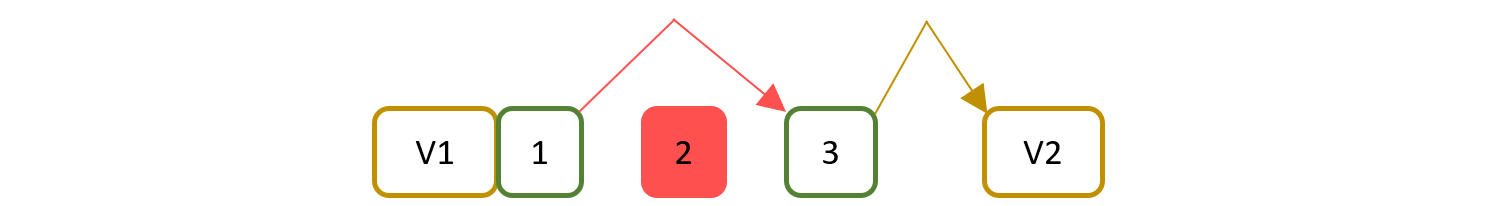 |
| mgFL  mgPALB2_5–12 | | | 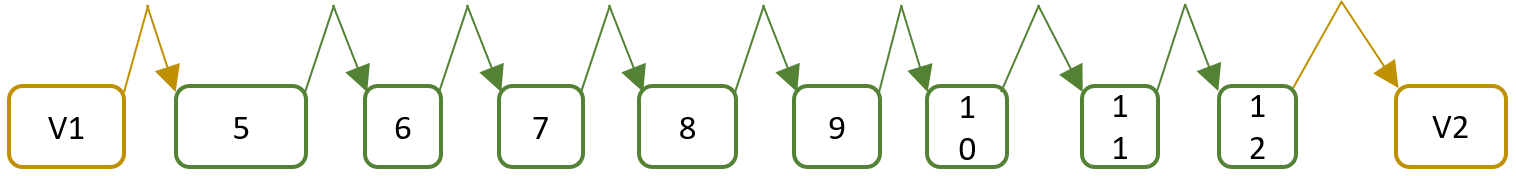 |
| △(E5p139) | r.1685_1823del  PTC-NMD | p.(Gly562Valfs*20) | 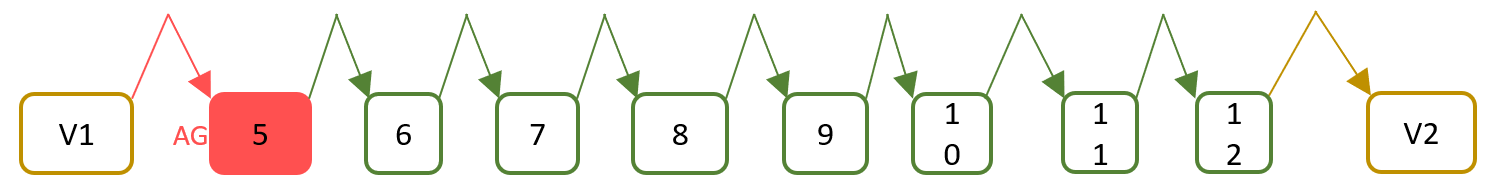 |
| ▼(E5q57) | r.2514_2515ins[gc;2514+3_2515+57]  PTC-NMD | p.(Gln838_Thr839ins*14) | 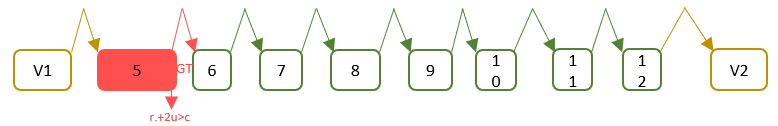 |
| ▼(E5q95) | r.2514_2515ins[gc;2514+3_2515+95]  PTC-NMD | p.(Gln838_Thr839ins*14) | 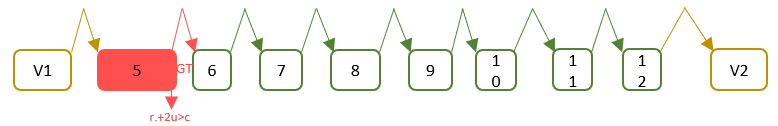 |
| ▼(E5q106) | r.2514_2515ins[gc;2514+3_2515+106]  PTC-NMD | p.(Gln838_Thr839ins*14) | 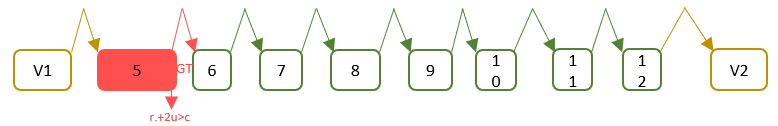 |
| △(E6) | r.2515_2586del | p.(Thr839_Lys862del) | 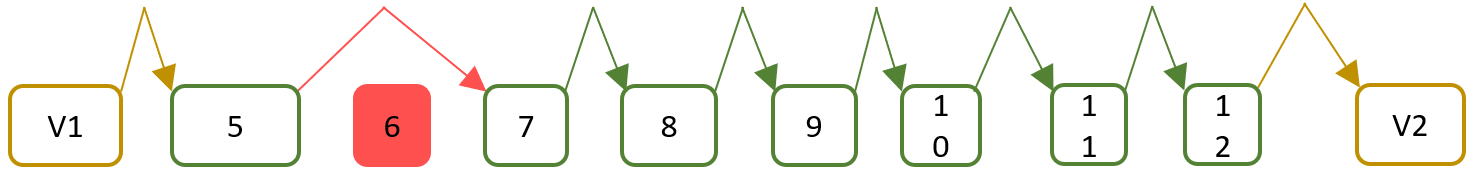 |
| △(E7) | r.2587_2748del | p.(Asn863_Glu916del) | 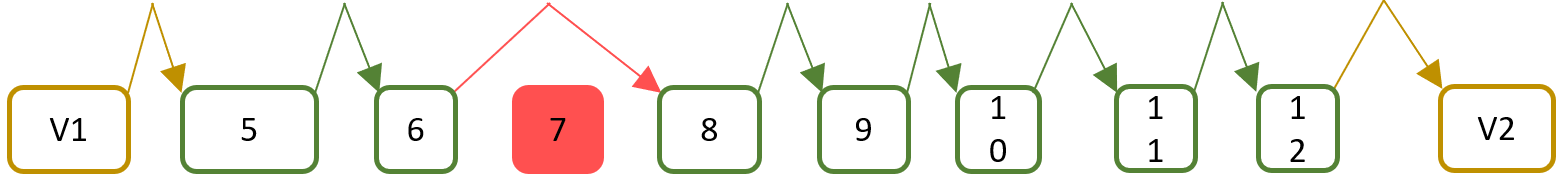 |
| △(E7p10) | r.2587_2596del  PTC-NMD | p.(Asn863Valfs*5) | 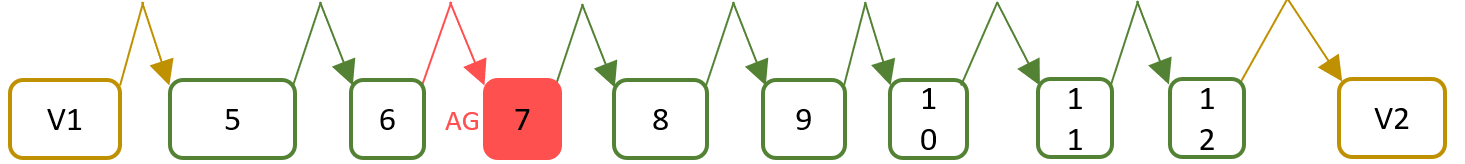 |
| △(E7)△(E9) | r.[2587_2748del, 2835_2996del] | p.([Asn863_Glu916del, Ala946_Gly999del]) | 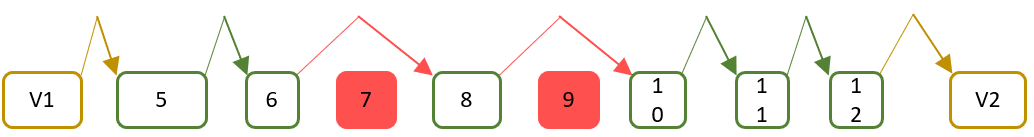 |
| △(E8) | r.2749_2834del  PTC-NMD | p.(Val917Glyfs*7) | 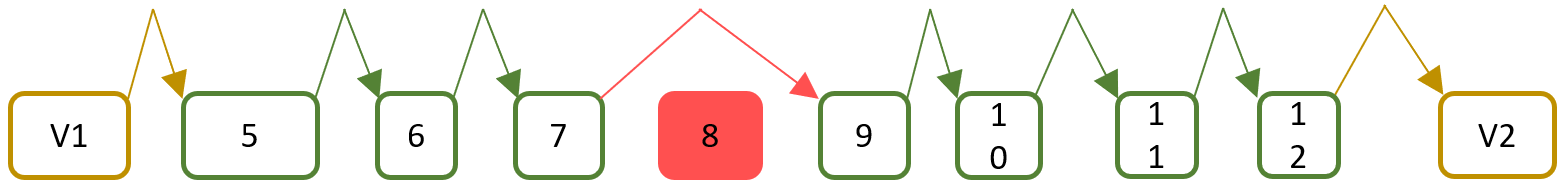 |
| △(E9) | r.2835_2996del | p.(Ala946_Gly999del) | 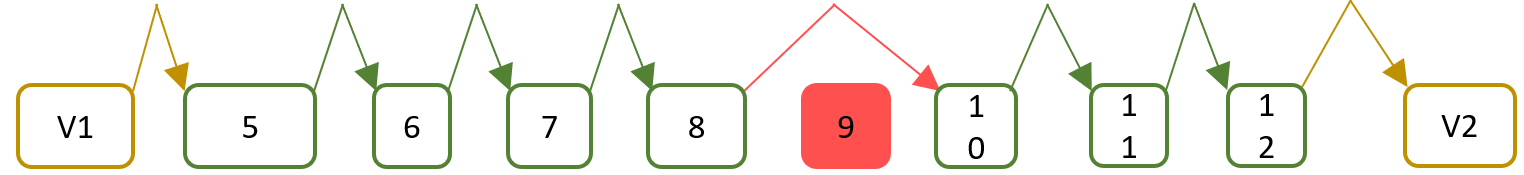 |
| △(E7p10)△(E9) | r.[2587_2596del, 2835_2996del]  PTC-NMD | p.(Asn863Valfs*5) | 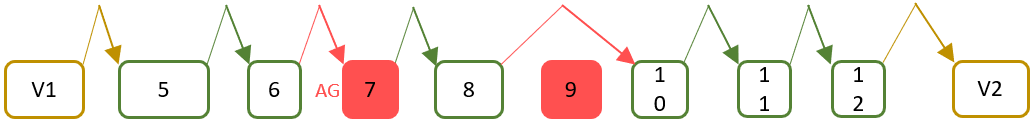 |
| △(E10) | r.2997_3113del | p.(Gly1000_Trp1038del) | 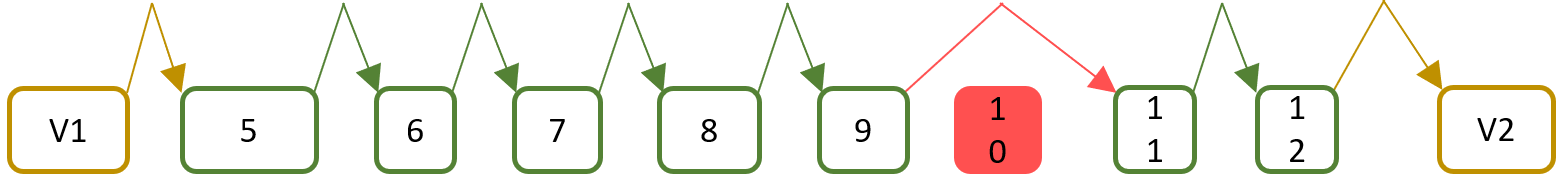 |
| △(E10q31) | r.3083_3113del  PTC-NMD | p.(Thr1029Ilefs*2) | 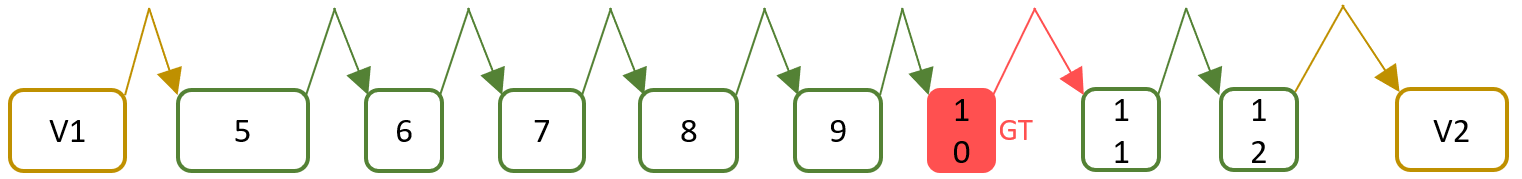 |
| △(E7p10)△(E10) | r.[2587_2596del, 2997_3113del]  PTC-NMD | p.(Asn863Valfs*5) | 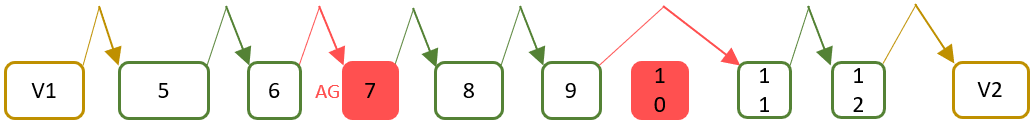 |
| △(E9_E10) | r.2835_3113del | p.(Ala946_Trp1038del) | 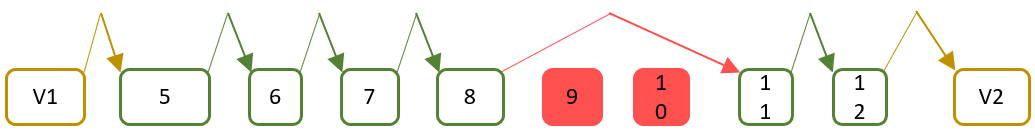 |
| △(E11) | r.3114_3201del  PTC-NMD | p.(Asn1039Glyfs*7) | 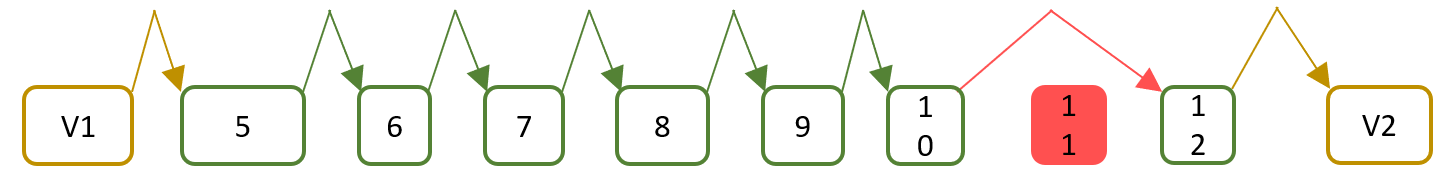 |
| △(E7p10)△(E11) | r.[2587_2596del, 3114_3201del]  PTC-NMD | p.(Asn863Valfs*5) | 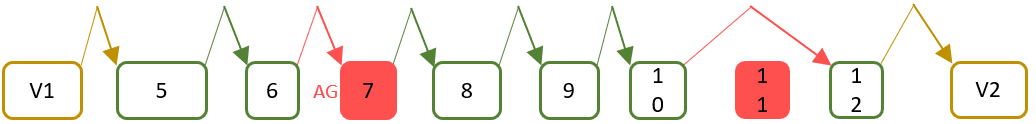 |
| △(E9)△(E11) | r.[2835_2996del, 3114_3201del]  PTC-NMD | p.([Ala946_Gly999del, Asn1039Glyfs*7]) | 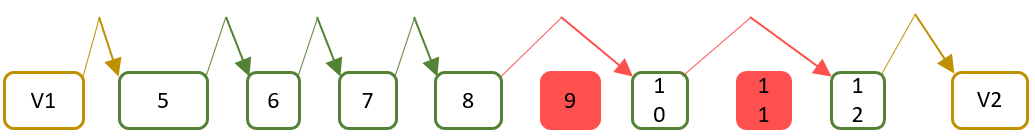 |

^a^ To simplify, splicing events were named with a short descriptor combining the following symbols: △ (skipping of exonic sequences), ▼ (inclusion of intronic sequences), E (exon), p (acceptor site shift) and q (donor site shift) [24]. Thus, △(E43p49) indicates the use of a cryptic alternative acceptor site 49-nt downstream, producing a 49-nt deletion. Seventy-two different transcripts were identified, 15 of which correspond to the minigene full-length and physiological alternative transcripts produced by the corresponding wild type minigenes. Sixteen minor transcripts could not be characterized. Up to 15 isoforms had been previously characterized as physiological alternative splicing events [23,54], including: *BRCA1* △(E13), △(E14), △(E13_E14), △(E14q11), ▼(E15q65), △(E16) and △(E17) and *PALB2* △(E2), △(E5q139), △(E7), △(E7p10), △(E9), △(E10), △(E9_10) and △(E10q31).

^b^ It is important to highlight that what is inserted is part of the fused intron of the mgATM_41–44.

^c^ The minigene intron (293 bp) is inserted, not the full *ATM* intron 43 (2,432 bp).

**Table S5.** DeepCLIP analysis of GC- and GG-donors (last 30 nucleotides of each exon): binding capacities of selected RNA binding proteins.

|  | hnRNP A1^a^ | hnRNP A1L2 | hnRNP A2B1 | hnRNP C^a^ | hnRNP CL1 | hnRNP H2 | hnRNP K^a^ | hnRNP L^a^ | hnRNP LL | hnRNP M^a^ | hnRNP U^a^ | hnRNP UL1^a^ |
| --- | --- | --- | --- | --- | --- | --- | --- | --- | --- | --- | --- | --- |
| GC-donors^b^ | 0.41 | 0.48 | 0.13 | 0.22 | 0.07 | 0.13 | 0.23 | 0.51 | 0.40 | 0.26 | 0.49 | 0.47 |
| *ATM* c.6347+2C | 0.36 | 0.05 | 0.09 | 0.28 | 0.16 | 0.13 | 0.52 | 0.55 | 0.95 | 0.66 | 0.64 | 0.60 |
| *BRCA1* c.5193+2C | 0.37 | 1.0 | 0.11 | 0.10 | 0.0 | 0.1 | 0.04 | 0.37 | 0.0 | 0.0 | 0.06 | 0.07 |
| *BRCA1* c.5277+2C | 0.73 | 1.0 | 0.22 | 0.24 | 0.0 | 0.02 | 0.33 | 0.54 | 0.74 | 0.07 | 0.66 | 0.63 |
| *PALB2* c.108+2C | 0.38 | 0.01 | 0.02 | 0.21 | 0.04 | 0.01 | 0.22 | 0.80 | 0.88 | 0.03 | 0.45 | 0.45 |
| *PALB2* c.2748+2C | 0.20 | 0.30 | 0.0 | 0.25 | 0.42 | 0.0 | 0.14 | 0.63 | 0.98 | 0.02 | 0.31 | 0.29 |
| *ATM* *ex50* | 0.52 | 1.0 | 0.02 | 0.19 | 0.0 | 0.12 | 0.04 | 0.33 | 0.0 | 0.28 | 0.59 | 0.59 |
| *BRCA2 ex17* | 0.49 | 0.99 | 0.58 | 0.12 | 0.0 | 0.15 | 0.02 | 0.51 | 0.0 | 0.04 | 0.33 | 0.35 |
| *PALB2 ex12* | 0.29 | 0.01 | 0.16 | 0.32 | 0.02 | 0.58 | 0.42 | 0.37 | 0.10 | 0.65 | 0.71 | 0.69 |
| GG-donors^c^ | 0.38 | 0.54 | 0.22 | 0.27 | 0.15 | 0.33 | 0.32 | 0.39 | 0.14 | 0.37 | 0.54 | 0.54 |
| *ATM* c.1898+2G | 0.08 | 0.98 | 0.0 | 0.58 | 1.0 | 0.01 | 0.27 | 0.35 | 0.0 | 0.02 | 0.10 | 0.14 |
| *ATM* c.6347+2G/A | 0.36 | 0.05 | 0.09 | 0.28 | 0.16 | 0.13 | 0.52 | 0.55 | 0.95 | 0.66 | 0.64 | 0.60 |
| GA-donors^c^ | 0.48 | 0.58 | 0.21 | 0.27 | 0.13 | 0.29 | 0.27 | 0.42 | 0.25 | 0.35 | 0.56 | 0.59 |
|  | **DAZAP1** | **SRSF1^a^** | **SRSF2** | **SRSF5** | **SRSF6** | **SRSF7^a^** | **SRSF9^a^** | **SRSF10^a^** | **TIA1^a^** | **Tra2α^a^** | **Tra2β** |  |
| GC-donors^b^ | 0.79 | 0.32 | 0.47 | 0.57 | 0.59 | 0.40 | 0.41 | 0.37 | 0.38 | 0.55 | 0.58 |  |
| *ATM* c.6347+2C | 0.88 | 0.40 | 0.05 | 0.68 | 0.67 | 0.37 | 0.39 | 0.02 | 0.38 | 0.70 | 0.56 |  |
| *BRCA1* c.5193+2C | 1.0 | 0.18 | 0.93 | 0.30 | 0.48 | 0.11 | 0.35 | 0.97 | 0.53 | 0.26 | 0.82 |  |
| *BRCA1* c.5277+2C | 0.41 | 0.86 | 0.99 | 0.51 | 0.78 | 0.78 | 0.85 | 0.96 | 0.17 | 0.89 | 0.89 |  |
| *PALB2* c.108+2C | 0.77 | 0.20 | 0.0 | 0.29 | 0.51 | 0.60 | 0.43 | 0.78 | 0.06 | 0.61 | 0.65 |  |
| *PALB2* c.2748+2C | 0.78 | 0.11 | 0.07 | 0.71 | 0.65 | 0.26 | 0.40 | 0.01 | 0.55 | 0.55 | 0.38 |  |
| *ATM* *ex50* | 0.87 | 0.46 | 0.99 | 0.70 | 0.76 | 0.43 | 0.55 | 0.1 | 0.44 | 0.87 | 0.91 |  |
| *BRCA2 ex17* | 0.98 | 0.11 | 0.29 | 0.48 | 0.70 | 0.26 | 0.20 | 0.21 | 0.60 | 0.12 | 0.31 |  |
| *PALB2 ex12* | 0.43 | 0.33 | 0.08 | 0.63 | 0.19 | 0.29 | 0.22 | 0.03 | 0.21 | 0.38 | 0.33 |  |
| GG-donors^c^ | 0.68 | 0.45 | 0.44 | 0.59 | 0.56 | 0.55 | 0.52 | 0.23 | 0.33 | 0.62 | 0.51 |  |
| *ATM* c.1898+2G | 0.89 | 0.12 | 0.77 | 0.70 | 0.33 | 0.08 | 0.31 | 0.06 | 0.58 | 0.11 | 0.43 |  |
| *ATM* c.6347+2G/A | 0.88 | 0.40 | 0.05 | 0.68 | 0.67 | 0.37 | 0.39 | 0.02 | 0.38 | 0.70 | 0.56 |  |
| GA-donors^c^ | 0.69 | 0.54 | 0.56 | 0.59 | 0.58 | 0.54 | 0.58 | 0.26 | 0.31 | 0.66 | 0.53 |  |

Binding scores  > 0.5 are highlighted in yellow. hnRNP, heterogeneous nuclear ribonucleoprotein. GC-, GA- and GG-donors are non-canonical 5’ splice sites

^a^ Average DeepCLIP binding scores.

^b^ Average binding scores of GC-exons of this and previous studies.

^c^ Average binding scores of all GG- and GA-annotated exons [8].
