# Supplementary material for: Generation of functional noncanonical donor splice sites by +2T variants in breast cancer susceptibility genes: impact on clinical interpretation
Source: J Pathol. 2025 Nov 13;268(2):150–63. doi: 10.1002/path.6497 (PMC12805630; doi:10.1002/path.6497)
Supplement: Supplementary file 1 — Figure S1. Insert sequences of minigenes mgATM_11–17, mgATM_41–44, mgBRCA1_13–19, mgPALB2_1–3 and mgPALB2_ex5–12 (provided as separate Word file) Figure S2. Splicing assays of additional +2T > C/G/A changes Figure S3. ClinGen/ACMG/AMP classification of 30 +2T > C/G/A variants Table S1. Splicing outcomes of previously studied +2T > C/G variants by minigene assays Table S2. Bioinformatics analysis of all +2T > C of the eight main breast cancer susceptibility genes (163 exons) and +2T > A/G variants tested in this study (provided as separate Word file) Table S3. Cloning and mutagenesis primers Table S4. Short descriptors and HGVS annotations of transcripts Table S5. DeepCLIP analysis of GC‐ and GG‐donors (last 30 nucleotides of each exon): binding capacities of selected RNA Binding Proteins Table S6. Clinical interpretation of 30 + 2 T variants (provided as separate Excel file) [file PATH-268-150-s001.zip › path6497-sup-0003-TableS2.docx]

**Generation of functional noncanonical donor splice sites by +2T variants in breast cancer susceptibility genes: impact on clinical interpretation**

I Llinares-Burguet *et al. J Pathol* <https://doi.org/10.1002/path.6497>

**Table S2.** Bioinformatics analysis of all +2T > C of the eight main breast cancer susceptibility genes (163 exons) and +2T > A/G variants tested in this study

**Table S2.** Bioinformatics analysis of all +2T > C of the eight main breast cancer susceptibility genes (163 exons) and +2T > A/G variants tested in this study.

| **Exon** | **Variants^a^** | **WT Sequence MAGgtragt^b^** | **MES wt^c^** | **MES +2^c^** | **SpliceAI analysis** | | | | | **mg-observed leakiness** |
| --- | --- | --- | --- | --- | --- | --- | --- | --- | --- | --- |
|  |  |  |  |  | **AL** | **AG** | **DL^d^** | **DG** | **Prediction** |  |
| ***ATM*** (NM_000051.4) | | | | | | | | | | |
| 1 | c.–31+2T > C | **GT**Ggta**g**g**g** | 4.2 | –3.6 | 0.02 (7665) |  | 0.72 (–2) | 0.06 (–120) | △(E1)/FL |  |
| 2 | c.72+2T > C | AAGgta**gta** | 5.7 | –2.1 | 0.84 (–103) |  | 0.86 (–2) |  | △(E2) |  |
| 3 | c.185+2T > C | **T**AGgta**ttc** | 4.6 | –3.2 | 0.13 (–110) |  | 0.97 (–2) | 0.45 (75) | ▼(E3q77) |  |
| 4 | c.331+2T > C | **G**AAgtaagt | 9.8 | 2.1 | 0.72 (–147) |  | 0.99 (–2) | 0.18 (48) | △(E4)/▼(E4q50) |  |
| 5 | c.496+2T > C | **T**AGgta**t**gt | 7.8 | 0.0 | 0.91 (–166) |  | 0.92 (–2) | 0.07 (–45) | △(E5) |  |
| 6 | c.662+2T > C | **G**AGgtaa**tc** | 7.1 | –0.7 | 0.93 (–167) |  | 0.96 (–2) |  | △(E6) |  |
| 7 | c.901+2T > C | AAGgta**taa** | 7.1 | –0.7 | 0.81 (–240) |  | 0.99 (–2) | 0.02 (–153) | △(E7) | No |
| 8 | c.1065+2T > C | CAGgta**cag** | 8.7 | 0.9 | 0.02 (1,804) |  | 0.97 (–2) | 0.26 (3) | ▼(E8q5) |  |
| 9 | c.1235+2T > C | **TT**Ggtaa**ag** | 4.1 | –3.7 | 0.44 (–171) | 0.02 (59) | 0.86 (–2) |  | △(E9) |  |
| 10 | c.1607+2T > C | A**T**Ggtaagt | 11.0 | 3.3 | 0.1 (763) |  | 0.63 (–2) | 0.08 (–179) | △(E10)/FL |  |
| 11 | c.1802+2T > C | CAGgtaa**t**t | 8.6 | 0.8 | 0.32 (–196) |  | 0.55 (–2) | 0.2 (–1,138) | △(E11)/FL |  |
| 12 | c.1898+2T > C | A**T**Ggta**t**gt | 8.4 | 0.6 | 0.57 (–97) | 0.02 (–2,213) | 0.95 (–2) | 0.51 (–64) | △(E12q62) / △(E12) | No |
|  | c.1898+2T > A |  |  | 0.2 | 0.54 (–97) | 0.02 (–2,213) | 1 (–2) | 0.49 (–64) | △(E12q62) / △(E12) | No |
|  | c.1898+2T > G |  |  | 0.7 | 0.57 (–97) | 0.02 (–2,213) | 1 (–2) | 0.49 (–64) | △(E12q62) / △(E12) | Yes (13%) |
| 13 | c.2124+2T > C | **G**AGgtgag**a** | 7.7 | –0.1 | 0.72 (–186) |  | 0.97 (–2) | 0.001 (–131) | △(E13) |  |
| 14 | c.2250+2T > C | AAGgta**g**g**a** | 9.5 | 1.7 | 0.94 (–127) |  | 0.99 (–2) | 0.01 (98) | △(E14) |  |
| 15 | c.2376+2T > C | AAGgtaag**a** | 10.6 | 2.8 | 0.86 (–137) | 0.06 (57) | 0.94 (–2) |  | △(E15) |  |
| 16 | c.2466+2T > C | **TTA**gtaagt | 7.8 | 0.0 | 0.74 (–91) | 0.02 (–1,175) | 0.95 (–2) | 0.18 (2) | △(E16) |  |
| 17 | c.2638+2T > C | **T**AGgtaa**a**t | 6.6 | –1.1 | 0.3 (–173) | 0.01 (–525) | 0.7 (–2) | 0.12 (–96) | △(E17)/FL |  |
| 18 | c.2838+2T > C | A**T**Ggtgagt | 10.1 | 2.4 | 0.31 (–201) |  | 0.98 (–2) | 0.19 (69) | △(E18) |  |
| 19 | c.2921+2T > C | A**TC**gtaag**a** | 6.3 | –1.5 | 0.87 (–84) | 0.06 (174) | 0.91 (–2) |  | △(E19) |  |
| 20 | c.3077+2T > C | **TT**Ggta**g**gt | 7.0 | –0.7 | 0.3 (1,124) |  | 0.99 (–2) | 0.74 (2) | ▼(E20q4) |  |
| 21 | c.3153+2T > C | **G**AGgtgagt | 10.0 | 2.3 | 0.86 (–77) |  | 0.93 (–2) | 0.01 (–1,463) | △(E21) |  |
| 22 | c.3284+2T > C | **T**AGgtaa**tg** | 7.6 | –0.2 | 0.48 (–132) |  | 0.99 (–2) | 0.01 (326) | △(E22) |  |
| 23 | c.3402+2T > C | A**T**Ggtaa**tt** | 6.5 | 1.3 | 0.58 (–119) |  | 0.99 (–2) | 0.12 (8) | △(E23) |  |
| 24 | c.3576+2T > C | AAGgta**ta**t | 7.8 | 0.1 | 0.74 (–83) | 0.01 (121) | 0.95 (–2) |  | △(E24) |  |
| 25 | c.3746+2T > C | **T**AGgtaagt | 9.7 | 1.9 | 0.41 (–171) | 0.01 (–894) | 0.73 (–2) | 0.01 (–72) | △(E25)/FL |  |
| 26 | c.3993+2T > C | CAGgta**t**g**g** | 10.0 | 2.2 | 0.06 (–91) | 0.02 (510) | 0.9 (–2) | 0.76 (–122) | △(E26q120) |  |
| 27 | c.4109+2T > C | A**G**Ggta**t**gt | 8.3 | 0.6 | 0.85 (–117) | 0.17 (–300) | 0.99 (–2) | 0.15 (–279) | △(E27) |  |
| 28 | c.4236+2T > C | C**CT**gtaagt | 7.5 | 0.2 | 0.53 (–128) |  | 0.99 (–2) | 0.03 (–86) | △(E28) |  |
| 29 | c.4436+2T > C | AAGgtaa**a**t | 8.9 | 1.1 | 0.35 (–201) |  | 0.96 (–2) | 0.02 (396) | △(E29) |  |
| 30 | c.4611+2T > C | CAGgtaa**t**t | 8.6 | 0.8 | 0.69 (–176) |  | 0.9 (–2) | 0.05 (25) | △(E30) |  |
| 31 | c.4776+2T > C | GAGgtaa**ta** | 8.0 | 0.2 | 0.92 (–166) |  | 0.97 (–2) |  | △(E31) |  |
| 32 | c.4909+2T > C | A**G**Ggtg**cta** | –2.3 | –10.0 | 0.04 (–134) |  | 0.64 (–2) | 0.01 (–114) | △(E32)/FL |  |
| 33 | c.5005+2T > C | **T**AGgtaa**ac** | 5.9 | –1.9 | 0.46 (–97) |  | 0.58 (–2) | 0.17 (80) | △(E33)/FL |  |
| 34 | c.5177+2T > C | **TT**Ggtgagt | 9.3 | 1.5 | 0.64 (–173) |  | 0.89 (–2) | 0.01 (2) | △(E34) |  |
| 35 | c.5319+2T > C | AAGgt**ctc**t | 0.9 | –6.9 | 0.31 (–143) |  | 0.82 (–2) | 0.04 (7) | △(E35) |  |
| 36 | c.5496+2T > C | GA**A**gtaag**a** | 6.4 | 1.3 | 0.14 (–171) |  | 0.97 (–2) | 0.17 (123) | △(E36) |  |
| 37 | c.5674+2T > C | CAGgta**ttc** | 6.5 | 1.3 | 0.55 (–179) |  | 0.83 (–2) | 0.01 (180) | △(E37) |  |
| 38 | c.5762+2T > C | **G**AGgtaa**tg** | 8.7 | 1.0 | 0.86 (–89) |  | 0.97 (–2) | 0.04 (3) | △(E38) |  |
| 39 | c.5918+2T > C | AAGgtaa**tg** | 9.0 | 1.2 | 0.8 (–157) |  | 0.99 (–2) | 0.16 (–91) | △(E39) |  |
| 40 | c.6006+2T > C | CAGgtaa**a**t | 8.8 | 1.0 | 0.68 (–89) | 0.01 (351) | 0.87 (–2) |  | △(E40) |  |
| 41 | c.6095+2T > C | **T**AGgtaa**a**t | 6.6 | –1.1 | 0.91 (–90) |  | 0.99 (–2) | 0.02 (23) | △(E41) | No |
| 42 | c.6198+2T > C | CAGgta**ca**t | 7.8 | 0.0 | 0.85 (–104) | 0.01 (–1,978) | 0.99 (–2) | 0.19 (19) | △(E42) | No |
| 43 | c.6347+2T > C | CAGgtaag**a** | 10.8 | 3.0 | 0.03 (–1,537) | 0.03 (–141) | 0.08 (–2) |  | FL | Yes (81%) |
|  | c.6347+2T > A |  |  | 2.6 | 0.54 (–101) |  | 0.98 (–2) | 0.07 (–7) | △(E43) | Yes (12%) |
|  | c.6347+2T > G |  |  | 3.1 | 0.57 (–101) |  | 0.96 (–2) | 0.03 (–7) | △(E43) | Yes (24%) |
| 44 | c.6452+2T > C | CAGgta**tta** | 7.3 | 0.5 | 0.51 (–106) |  | 0.99 (–2) | 0.17 (80) | △(E44) | No |
| 45 | c.6572+2T > C | AAGgta**t**gt | 9.8 | 2.0 | 0.54 (–146) |  | 0.71 (–2) | 0.01 (–99) | △(E45)/FL |  |
| 46 | c.6807+2T > C | CAGgtaa**a**t | 8.8 | 1.0 | 0.47 (–155) |  | 0.54 (–2) | 0.01 (–73) | △(E46)/FL |  |
| 47 | c.6975+2T > C | **GC**Ggt**tt**gt | 4.0 | –3.7 | 0.43 (–169) |  | 0.98 (–2) | 0.01 (55) | △(E47) |  |
| 48 | c.7089+2T > C | AAGgtaag**a** | 10.6 | 2.8 | 0.7 (–115) |  | 0.93 (–2) | 0.01 (91) | △(E48) |  |
| 49 | c.7307+2T > C | CAGgtaa**c**t | 8.6 | 0.9 | 0.22 (–137) |  | 0.96 (–2) | 0.2 (46) | △(E49)/▼(E49q48) |  |
| 50 | c.7515 | AAGgcaagt  **Natural GC** | 3.2 |  |  |  |  |  |  |  |
| 51 | **c.7629+2T > C** | AA**T**gtaagt | 8.6 | 0.9 | 0.17 (–115) | 0.03 (306) | 0.49 (–2) | 0.41 (2) | ▼(E51q4)/FL |  |
| 52 | c.7788+2T > C | **G**AGgta**tt**t | 7.6 | 0.1 | 0.83 (–160) | 0.02 (–2) | 0.81 (–2) |  | △(E52) |  |
| 53 | c.7927+2T > C | **G**A**A**gta**t**gt | 7.0 | –0.8 | 0.51 (–140) |  | 0.97 (–2) | 0.05 (–81) | △(E53) |  |
| 54 | c.8010+2T > C | AAGgtaa**t**t | 8.8 | 1.1 | 0.98 (–84) |  | 0.99 (–2) |  | △(E54) |  |
| 55 | c.8151+2T > C | AAGgtgag**c** | 9.6 | 1.8 | 0.47 (–142) |  | 0.76 (–2) | 0.14 (–70) | △(E55)/FL |  |
| 56 | c.8268+2T > C | AAGgtaa**c**t | 9.0 | 1.3 | 0.6 (–118) |  | 0.96 (–2) | 0.02 (–13) | △(E56) |  |
| 57 | c.8418+2T > C | A**T**Ggtgagt | 10.1 | 2.4 | 0.8 (–151) |  | 0.88 (–2) | 0.16 (21) | △(E57) |  |
| 58 | c.8584+2T > C | **TT**Ggtaat**c** | 3.4 | –4.4 | 0.27 (–167) |  | 0.98 (–2) | 0.24 (115) | △(E58)/▼(E58q117) |  |
| 59 | c.8671+2T > C | **T**AGgtaagt | 9.7 | 1.9 | 0.46 (–88) |  | 0.72 (–2) | 0.01 (2) | △(E59)/FL |  |
| 60 | c.8786+2T > C | AAGgtaagt | 11.0 | 3.2 | 0.41 (–116) |  | 0.33 (–2) | 0.05 (12) | △(E60)/FL |  |
| 61 | c.8850+2T > C | **G**AGgtaa**ag** | 8.3 | 0.5 | 0.84 (–65) | 0.15 (42) | 0.94 (–2) |  | △(E61) |  |
| 62 | c.8987+2T > C | CAGgtgag**c** | 9.6 | 1.9 | 0.08 (105) | 0.03 (496) | 0.13 (–2) | 0.84 (18) | ▼(E62q20)/FL |  |
| ***BRCA1*** (NM_007294.4) | | | | | | | | | | |
| 1 | c.–20+2T > C | AAGgta**gta** | 5.7 | –2.1 | 0.01 (–1,154) |  | 0.8 (2) | 0.2 (–87) | ▼(E1q89) |  |
| 2 | c.80+2T > C | C**T**Ggtaagt | 10.7 | 2.9 | 0.89 (100) |  | 0.93 (2) | 0.01 (1,256) | △(E2) |  |
| 3 | c.134+2T > C | CA**A**gtaagt | 10.1 | 2.3 | 0.93 (55) |  | 0.97 (2) |  | △(E3) |  |
| 4 | c.212+2T > C | A**G**Ggta**ta**t | 3.9 | –3.9 | 0.28 (79) |  | 0.65 (2) | 0.03 (–10) | △(E4)/FL |  |
| 5 | c.301+2T > C | A**GT**gtaagt | 8.5 | 0.7 | 0.37 (90) | 0.04 (20) | 0.67 (2) | 0.09 (–104) | △(E5)/FL |  |
| 6 | c.441+2T > C | **TT**Ggtaa**aa** | 3.2 | –4.5 | 0.05 (141) | 0.04 (836) | 0.96 (2) | 0.42 (64) | △(E6q62) |  |
| 7 | c.547+2T > C | **TG**Ggtaag**g** | 9.1 | 1.3 | 0.95 (107) |  | 0.99 (2) | 0.01 (–11) | △(E7) |  |
| 8 | c.593+2T > C | CAGgtgagt | 10.7 | 2.9 | 0.92 (47) |  | 0.96 (2) | 0.05 (–31) | △(E8) |  |
| 9 | c.670+2T > C | A**G**Ggtaa**tg** | 6.0 | –1.8 | 0.77 (78) |  | 0.79 (2) | 0.03 (–40) | △(E9)/FL |  |
| 10 | c.4096+2T > C | **T**AGgta**ttg** | 5.6 | –2.1 | 0.16 (200) |  | 0.69 (2) | 0.09 (–43) | △(E10)/FL |  |
| 11 | c.4185+2T > C | CAGgtaa**aa** | 8.6 | 0.8 | 0.92 (90) |  | 0.99 (2) |  | △(E11) |  |
| 12 | c.4357+2T > C | AAGgtg**t**gt | 6.6 | –1.1 | 0.39 (170) |  | 0.51 (2) | 0.05 (0) | △(E12)/FL |  |
| 13 | c.4484+2T > C | AAGgtaag**a** | 10.6 | 2.8 | 0.73 (125) |  | 0.96 (2) |  | △(E13) | No |
| 14 | c.4675+2T > C | **T**AGgtaa**ta** | 6.8 | –0.9 | 0.30 (192) | 0.01 (584) | 0.96 (2) | 0.44 (13) | △(E14)/△(E14q11) | No |
| 15 | c.4986+2T > C | **TTT**gtgagt | 5.9 | –1.9 | 0.11 (–3,231) | 0.01 (376) | 0.99 (2) | 0.57 (–63) | ▼(E15q65) | No |
| 16 | c.5074+2T > C | CAGgta**tac** | 7.5 | –0.3 | 0.26 (89) |  | 0.74 (2) | 0.05 (–151) | △(E16)/FL | No |
| 17 | c.5152+2T > C | **TCT**gtaagt | 8.0 | 0.2 | 0.82 (79) |  | 0.9 (2) |  | △(E17) | No |
| 18 | c.5193+2T > C | **G**AGgtaagt | 11.1 | 3.3 | 0.24 (42) |  | 0.53 (2) | 0.01 (76) | △(E18)/FL | Yes (61%) |
|  | c.5193+2T > A |  |  | 2.9 | 0.71 (42) |  | 0.98 (2) | 0.04 (76) | △(E19) / ▼(E19q87) | No |
|  | c.5193+2T > G |  |  | 3.4 | 0.68 (42) |  | 0.98 (2) | 0.04 (76) | △(E19) / ▼(E19q87) | No |
| 19 | c.5277+2T > C | AAGgtaa**ag** | 9.1 | 1.3 | 0.47 (85) |  | 0.82 (2) | 0.36 (–85) | △(E19)/▼(E19q87) | Yes (14%) |
|  | c.5277+2T > A |  |  | 0.9 | 0.31 (85) |  | 1 (2) | 0.64 (–85) | △(E19)/▼(E19q87) | No |
|  | c.5277+2T > G |  |  | 1.4 | 0.51 (85) |  | 1 (2) | 0.43 (–85) | △(E19)/▼(E19q87) | No |
| 20 | c.5332+2T > C | CAGgtaag**a** | 10.8 | 3.0 | 0.83 (569 |  | 0.9 (2) | 0.11 (–1,279) | △(E20) |  |
| 21 | c.5406+2T > C | A**CA**gtaagt | 9.5 | 1.7 | 0.16 (75) | 0.01 (1071) | 0.96 (2) | 0.69 (–154) | ▼(E22q156) |  |
| 22 | c.5467+2T > C | A**T**Ggtaag**g** | 9.3 | 1.6 | 0.58 (62) |  | 0.93 (2) | 0.15 (–3) | △(E22) |  |
| ***BRCA2*** (NM_000059.4) | | | | | | | | | | |
| 1 | c.–40+2T > C | CGGgttagt | 4.9 | –2.8 | 0.08 (753) | 0.01 (534) | 0.98 (–2) | 0.62 (–101) | △(E1q99) |  |
| 2 | c.67+2T > C | CAGgta**ttg** | 8.4 | 0.6 | 0.8 (–107) |  | 0.94 (–2) | 0.18 (–138) | △(E2) | No |
| 3 | c.316+2T > C | **T**AGgtaagt | 9.7 | 1.9 | 0.64 (–250) | 0.03 (–1545) | 0.95 (–2) | 0.01 (–36) | △(E3) | No |
| 4 | c.425+2T > C | AAGgta**t**g**a** | 9.1 | 1.4 | 0.96 (–110) | 0.01 (–528) | 0.99 (–2) |  | △(E4) |  |
| 5 | c.475+2T > C | CAGgta**t**g**a** | 9.5 | 1.7 | 0.94 (–51) | 0.01 (356) | 0.98 (–2) |  | △(E5) |  |
| 6 | c.516+2T > C | AAGgtaa**a**t | 8.9 | 1.1 | 0.93 (–42) | 0.02 (224) | 0.97 (–2) | 0.03 (329) | △(E6) | No |
| 7 | c.631+2T > C | **T**AGgtaa**ta** | 6.8 | –0.9 | 0.59 (–116) |  | 0.82 (–2) | 0.01 (40) | △(E7) |  |
| 8 | c.681+2T > C | **GCT**gtaagt | 8.6 | 1.8 | 0.18 (–47) | 0.01 (–1680) | 0.97 (–2) | 0.9 (2) | ▼(E8q4) |  |
| 9 | c.793+2T > C | A**T**Ggtaagt | 11.0 | 3.3 | 0.57 (–113) | 0.02 (–1585) | 0.76 (–2) | 0.02 (26) | △(E9)/FL |  |
| 10 | c.1909+2T > C | CAGgta**cc**t | 8.2 | 0.4 | 0.03 (2,514) |  | 0.98 (–2) | 0.05 (–179) | △(E10) |  |
| 11 | c.6841+2T > C | **TG**Ggtaagt | 10.2 | 2.5 | 0.04 (–190) |  | 0.72 (–2) | 0.01 (–2,189) | △(E11)/FL |  |
| 12 | c.6937+2T > C | A**T**Ggtaa**aa** | 4.8 | –3.0 | 0.84 (–97) |  | 0.95 (–2) | 0.09 (39) | △(E12) |  |
| 13 | c.7007+2T > C | **TC**Ggtaag**a** | 10.5 | 2.8 | 0.92 (–71) |  | 0.99 (–2) |  | △(E13) |  |
| 14 | c.7435+2T > C | **TA**Ggta**ttg** | 5.6 | –2.1 | 0.12 (–429) |  | 0.99 (–2) | 0.33 (3) | ▼(E14q5) |  |
| 15 | c.7617+2T > C | CAGgta**t**gt | 9.8 | 2.1 | 0.75 (–183) |  | 0.72 (–2) | 0.01 (–94) | △(E15)/FL |  |
| 16 | c.7805+2T > C | **T**AGgta**ctc** | 4.7 | –3.1 | 0.06 (–145) | 0.07 (–450) | 0.98 (–2) | 0.35 (–102) | △(E16q100) |  |
| 17 | c.7976 | CAGgcaagt **Natural GC** | 3.1 |  |  |  |  |  |  |  |
| 18 | c.8331+2T > C | AAGgtaa**a**t | 8.9 | 1.1 | 0.13 (–356) |  | 0.93 (–2) | 0.02 (–159) | △(E18) | No |
| 19 | c.8487+2T > C | CAGgta**t**g**a** | 9.5 | 1.7 | 0.85 (–157) |  | 0.96 (–2) | 0.02 (–32) | △(E19) |  |
| 20 | c.8632+2T > C | AAGgtaa**aa** | 8.4 | 0.6 | 0.39 (–146) |  | 0.98 (–2) | 0.67 (15) | △(E20)/▼(E20q17) |  |
| 21 | c.8754+2T > C | **G**AGgtgag**a** | 7.7 | –0.1 | 0.01 (3,008) |  | 0.99 (–2) | 0.76 (44) | ▼(E21q46) |  |
| 22 | c.8953+2T > C | CAGgtaagt | 10.9 | 3.1 | 0.02 (284) | 0.02 (–189) | 0.22 (–2) | 0.66 (2) | ▼(E22q4)/FL |  |
| 23 | c.9117+2T > C | CCGgtacaa | 4.3 | –3.5 | 0.8 (–165) | 0.03 (116) | 0.93 (–2) | 0.05 (14) | △(E23) |  |
| 24 | c.9256+2T > C | CAGgtaa**tg** | 9.4 | 1.7 | 0.43 (–140) |  | 0.95 (–2) | 0.1 (–45) | △(E24) |  |
| 25 | c.9501+2T > C | **G**AGgtaag**g** | 10.3 | 2.5 | 0.09 (–246) | 0.01 (–626) | 0.74 (–2) | 0.4 (32) | ▼(E25q34)/FL |  |
| 26 | c.9648+2T > C | C**T**Ggtaagt | 10.7 | 2.9 | 0.5 (–148) | 0.04 (–1,055) | 0.97 (–2) | 0.02 (81) | △(E26) |  |
| ***BARD1*** (NM_000465.4) | | | | | | | | | | |
| 1 | c.158+2T > C | **TT**Ggtaa**ag** | 4.1 | –3.7 | 0.02 (–110) | 0.07 (–726) | 0.97 (2) | 0.4 (–268) | ▼(E1q270) |  |
| 2 | c.215+2T > C | **T**AGgtaagt | 9.7 | 1.9 | 0.8 (58) |  | 0.92 (2) | 0.01 (–2) | △(E2) |  |
| 3 | c.364+2T > C | CAGgtaag**a** | 10.8 | 3.0 | 0.51 (150) |  | 0.82 (2) | 0.18 (41) | △(E3) |  |
| 4 | c.1314+2T > C | AAGgta**g**g**a** | 9.5 | 1.7 | 0.08 (951) |  | 0.9 (2) | 0.02 (139) | △(E4) |  |
| 5 | c.1395+2T > C | **TT**Ggta**gt**t | 0.6 | –7.1 | 0.26 (82) |  | 0.98 (2) | 0.47 (–9) | ▼(E5q11)/△(E5) |  |
| 6 | c.1568+2T > C | **TGT**gtaagt | 7.7 | –0.1 | 0.24 (174) | 0.01 (1832) | 0.99 (2) | 0.25 (–54) | ▼(E6q56)/△(E6) |  |
| 7 | c.1677+2T > C | **GTA**gtaagt | 8.0 | 0.3 | 0.85 (110) | 0.01 (1525) | 0.92 (2) | 0.06 (–2) | △(E7) |  |
| 8 | c.1810+2T > C | CAGgtgag**g** | 10.1 | 2.3 | 0.84 (134) |  | 0.98 (2) | 0.2 (56) | △(E8)/△(E8q54) |  |
| 9 | c.1903+2T > C | AA**T**gtaagt | 8.6 | 0.9 | 0.81 (94) |  | 0.96 (2) |  | △(E9) |  |
| 10 | c.2001+2T > C | C**T**Ggta**tt**t | 3.5 | –4.3 | 0.05 (–129) | 0.01 (–63) | 0.97 (2) | 0.46 (–92) | ▼(E10q94) |  |
| ***CHEK2*** (NM_007194.4) | | | | | | | | | | |
| 1 | c.–7+2T > C | **GC**Ggtgagt | 10.5 | 2.7 | 0.02 (–3,570) | 0.01 (–148) | 0.97 (2) | 0.61 (15) | △(E1q13) |  |
| 2 | c.319+2T > C | **TT**Ggtaag**a** | 8.9 | 1.1 | 0.76 (326) |  | 0.97 (2) | 0.01 (–1,258) | △(E2) |  |
| 3 | c.444+2T > C | A**G**Ggta**g**gt | 8.1 | 0.3 |  | 0.02 (–121) | 0.98 (2) | 0.94 (–2) | ▼(E3q4) |  |
| 4 | c.592+2T > C | AAGgtaa**ta** | 8.5 | 0.7 | 0.91 (149) | 0.12 (–52) | 0.99 (2) | 0.05 (–89) | △(E4) |  |
| 5 | c.683+2T > C | AAGgtaa**at** | 8.9 | 1.1 | 0.94 (92) | 0.07 (2,238) | 0.96 (2) |  | △(E5) |  |
| 6 | c.792+2T > C | **GCA**gtaagt | 9.1 | 1.3 | 0.27 (110) |  | 0.95 (2) | 0.84 (–117) | ▼(E6q119) |  |
| 7 | c.846+2T > C | CA**T**gtaagt | 8.3 | 0.6 | 0.94 (55) |  | 0.96 (2) |  | △(E7) |  |
| 8 | c.908+2T > C | A**TT**gtaagt | 8.5 | 0.8 | 0.87 (63) |  | 0.88 (2) |  | △(E8) |  |
| 9 | c.1008+2T > C | CAGgtaa**aa** | 8.6 | 0.8 | 0.7 (101) |  | 0.97 (2) | 0.03 (–106) | △(E9) |  |
| 10 | c.1095+2T > C | AAGgtaag**a** | 10.6 | 2.8 | 0.63 (88) |  | 0.72 (2) | 0.01 (–16) | △(E10)/FL |  |
| 11 | c.1259+2T > C | C**T**Ggtaag**a** | 9.5 | 1.7 | 0.83 (165) |  | 0.95 (2) | 0.11 (–29) | △(E11) |  |
| 12 | c.1375+2T > C | AAGgta**t**g**a** | 9.1 | 1.4 | 0.75 (117) |  | 0.82 (2) | 0.06 (–28) | △(E12) |  |
| 13 | c.1461+2T > C | CAGgtg**g**gt | 8.6 | 0.8 | 0.77 (87) |  | 0.99 (2) | 0.04 (–664) | △(E13) |  |
| 14 | c.1542+2T > C | CAGgta**ttc** | 6.5 | –1.3 | 0.16 (82) |  | 0.97 (2) | 0.49 (14) | △(E14q12) |  |
| ***PALB2*** (NM_024675.4) | | | | | | | | | | |
| 1 | c.48+2T > C | AAGgtg**ccg** | 5.7 | –2.0 |  | 0.03 (–612) | 0.94 (2) | 0.33 (–7) | ▼(E1q9) | No |
| 2 | c.108+2T > C | CAGgtaagt | 10.9 | 3.1 | 0.22 (–80) | 0.01 (524) | 0.31 (2) | 0.02 (405) | △(E2)/FL | Yes (86%) |
|  | c.108+2T > A |  |  | 2.7 | 0.85 (61) |  | 0.99 (2) | 0.16 (–25) | △(E2) | No |
|  | c.108+2T > G |  |  | 3.2 | 0.81 (61) |  | 0.99 (2) | 0.12 (–25) | △(E2) | No |
| 3 | c.211+2T > C | CAGgtaa**a**t | 8.8 | 1.0 | 0.32 (104) |  | 0.99 (2) | 0.59 (–46) | △(E3)/▼(E3q48) | No |
| 4 | c.1684+2T > C | AAGgtaa**a**t | 8.9 | 1.1 | 0.05 (63) |  | 0.94 (2) | 0.01 (–559) | △(E4) |  |
| 5 | c.2514+2T > C | CAGgta**caa** | 7.1 | –0.7 | 0.19 (–363) |  | 0.98 (2) | 0.38 (–104) | ▼(E5q106) | No |
| 6 | c.2586+2T > C | AAGgt**c**ag**a** | 6.8 | –1.0 | 0.36 (73) |  | 0.97 (2) | 0.03 (–110) | △(E6) | No |
| 7 | c.2748+2T > C | **G**AGgtaagt | 11.1 | 3.3 | 0.66 (163) |  | 0.72 (2) |  | △(E7)/FL | Yes (4%) |
|  | c.2748+2T > A |  |  | 2.9 | 0.67 (163) |  | 0.73 (2) |  | △(E7)/FL | No |
|  | c.2748+2T > G |  |  | 3.4 | 0.67 (163) |  | 0.73 (2) |  | △(E7)/FL | No |
| 8 | c.2834+2T > C | CAGgta**t**gt | 9.8 | 2.1 | 0.92 (87) |  | 0.98 (2) |  | △(E8) | No |
| 9 | c.2996+2T > C | A**G**Ggtaag**a** | 9.2 | 1.5 | 0.95 (163) |  | 0.97 (2) |  | △(E9) | No |
| 10 | c.3113+2T > C | **TT**Ggtaag**c** | 8.7 | 1.0 | 0.7 (118) |  | 0.99 (2) | 0.16 (33) | △(E10) | No |
| 11 | c.3201+2T > C | A**T**Ggtaagt | 11.0 | 3.3 | 0.88 (89) |  | 0.92 (2) | 0.01 (–59) | △(E11) | No |
| 12 | c.3350 | CAGgcaagt  **Natural GC** | 3.1 |  |  |  |  |  |  |  |
| ***RAD51C*** (NM_058216.3) | | | | | | | | | | |
| 1 | c.145+2T > C | AAGgtaa**cg** | 10.2 | 2.4 |  | 0.01 (2,144) | 0.86 (–2) | 0.21 (169) | ▼(Eq171) |  |
| 2 | c.404+2T > C | A**T**Ggtaa**aa** | 7.8 | 0.0 | 0.29 (–257) |  | 0.97 (–2) | 0.52 (25) | ▼(E2q27) | No |
| 3 | c.571+2T > C | A**G**Ggtaagt | 10.5 | 2.7 | 0.95 (–168) |  | 0.99 (–2) | 0.03 (2) | △(E3) |  |
| 4 | c.705+2T > C | AAGgta**t**g**a** | 9.1 | 1.4 | 0.69 (–135) |  | 0.98 (–2) | 0.07 (20) | △(E4) |  |
| 5 | c.837+2T > C | G**CT**gtaagt | 4.8 | –3.0 | 0.67 (–133) |  | 0.99 (–2) | 0.01 (2) | △(E5) | No |
| 6 | c.904+2T > C | **T**AGgtg**g**gt | 5.6 | –2.2 | 0.38 (–68) |  | 0.95 (–2) | 0.42 (2) | ▼(E6q4)/△(E6) |  |
| 7 | c.965+2T > C | AAGgt**c**agt | 8.7 | 0.9 | 0.82 (–62) | 0.02 (–929) | 0.94 (–2) |  | △(E7) |  |
| 8 | c.1026+2T > C | AA**A**gt**c**agt | 2.0 | –5.8 | 0.74 (–62) |  | 0.88 (–2) | 0.07 (42) | △(E8) |  |
| ***RAD51D*** (NM_002878.4) | | | | | | | | | | |
| 1 | c.82+2T > C | CAGgtga**cc** | 6.5 | –1.2 | 0.04 (–358) |  | 0.96 (2) | 0.31 (–6) | ▼(E1q8) |  |
| 2 | c.144+2T > C | AAGgtgag**c** | 9.6 | 1.8 | 0.65 (63) |  | 0.95 (2) | 0.14 (32) | △(E2) |  |
| 3 | c.263+2T > C | CAGgt**tt**gt | 7.4 | –0.3 | 0.67(1,209 |  | 0.96 (2) | 0.1 (–2) | △(E3) |  |
| 4 | c.345+2T > C | CAGgta**ca**t | 8.6 | 0.8 | 0.71 (83) |  | 0.92 (2) | 0.01 (19) | △(E4) | No |
| 5 | c.480+2T > C | CAGgtaag**g** | 11.1 | 3.3 | 0.91 (136) |  | 0.97 (2) | 0.01 (137) | △(E5) |  |
| 6 | c.576+2T > C | CAGgtgag**c** | 9.6 | 1.9 | 0.17 (–135) | 0.02 (–63) | 0.49 (2) | 0.49 (–256) | ▼(E6q258)/FL |  |
| 7 | c.667+2T > C | AAGgtgagt | 10.5 | 2.7 | 0.47 (92) |  | 0.92 (2) | 0.29 (17) | △(E7) / △(E7q15) |  |
| 8 | c.738+2T > C | **GT**Ggtgag**g** | 6.1 | –1.6 | 0.29 (72) |  | 0.95 (2) | 0.06 (–35) | △(E8) |  |
| 9 | c.903+2T > C | CAGgtgag**c** | 9.6 | 1.9 | 0.47 (–163) | 0.02 (14,229 | 0.95 (2) | 0.02 (–947) | △(E9) |  |

^a^ Initially pre-selected variants (MES GT-5’SS ≥ 10.8; SpliceAI-DL score < 0.8) are yellow-shadowed. Other 11 potentially candidate variants with MES score ≥ 10.6 (MES average of leaky variants of this study) are highlighted in blue: *ATM* c.2376+2T > C and c.7089+2T > C; *BRCA1* c.80+2T > C, c.593+2T > C, c.4484+2T > C and c.5332+2T > C; *BRCA2* c.9648+2T > C; *BARD1* c.364+2T > C; *CHEK2* c.1095+2T > C; *PALB2* c.3201+2T > C; *RAD51D* c.480+2T > C.

^b^ Positions that do not match the consensus sequence are indicated in red. The conserved central core of six nucleotides (AGgtaa) in the +2T > C full-length producing variants is underlined

^c^ MES wt scores ≥ 10.8 and mutant scores ≥ 3.0 are highlighted in yellow.

^d^ DL scores ≤ 0.8 are highlighted in yellow.

WT Sequence, Wild-type sequence; MES wt, MaxEntScan score of the wild-type sequence; AL, acceptor loss; AG, acceptor gain; DL, donor loss; DG, donor gain.
