# Supplementary material for: Generation of functional noncanonical donor splice sites by +2T variants in breast cancer susceptibility genes: impact on clinical interpretation
Source: J Pathol. 2025 Nov 13;268(2):150–63. doi: 10.1002/path.6497 (PMC12805630; doi:10.1002/path.6497)
Supplement: Supplementary file 1 — Figure S1. Insert sequences of minigenes mgATM_11–17, mgATM_41–44, mgBRCA1_13–19, mgPALB2_1–3 and mgPALB2_ex5–12 (provided as separate Word file) Figure S2. Splicing assays of additional +2T > C/G/A changes Figure S3. ClinGen/ACMG/AMP classification of 30 +2T > C/G/A variants Table S1. Splicing outcomes of previously studied +2T > C/G variants by minigene assays Table S2. Bioinformatics analysis of all +2T > C of the eight main breast cancer susceptibility genes (163 exons) and +2T > A/G variants tested in this study (provided as separate Word file) Table S3. Cloning and mutagenesis primers Table S4. Short descriptors and HGVS annotations of transcripts Table S5. DeepCLIP analysis of GC‐ and GG‐donors (last 30 nucleotides of each exon): binding capacities of selected RNA Binding Proteins Table S6. Clinical interpretation of 30 + 2 T variants (provided as separate Excel file) [file PATH-268-150-s001.zip › path6497-sup-0001-FigureS1.docx]

**Generation of functional noncanonical donor splice sites by +2T variants in breast cancer susceptibility genes: impact on clinical interpretation**

I Llinares-Burguet *et al. J Pathol* <https://doi.org/10.1002/path.6497>

**Figure S1.** Insert sequences of minigenes mgATM_11–17, mgATM_41–44, mgBRCA1_13–19, mgPALB2_1–3 and mgPALB2_ex5–12

**S1A.** Insert sequence of minigene mgATM_11–17

**S1B.** Insert sequence of minigene mgATM_41–44

**S1C.** Insert sequence of minigene mgBRCA1_13–19

**S1D.** Insert sequence of minigene mgPALB2_1–3

**S1E.** Insert sequence of minigene mgPALB2_5–12

**Reference numbers refer to the main text list.**

**
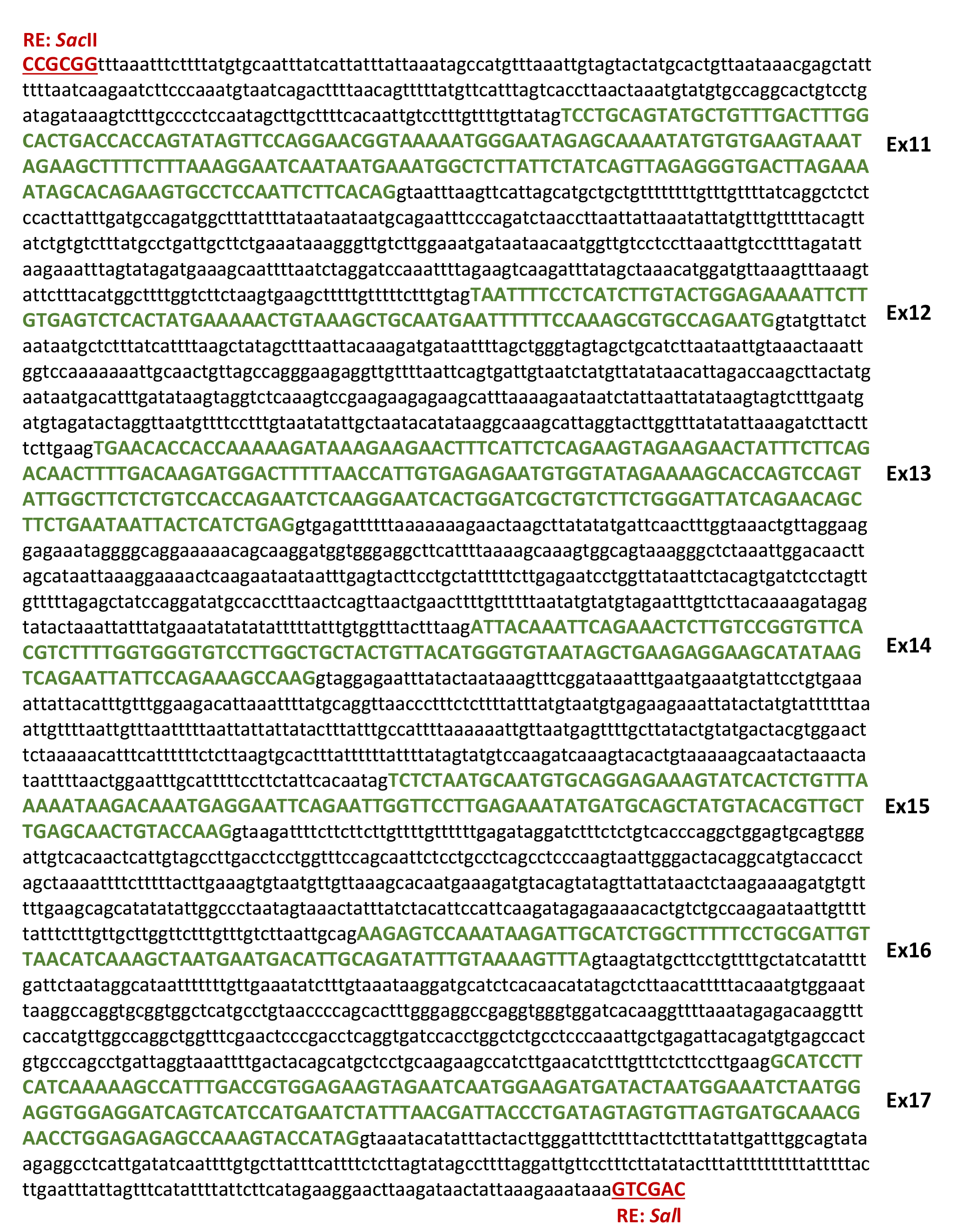
**

**Figure S1A. Insert sequence of minigene mgATM_11–17. Size:** 3,911 bp. **Structure:** ivs10 (250 bp) – ex11 (195 bp) – ivs11-1 (200 bp) // ivs11-2 (200 bp) – ex12 (96 bp) – ivs12-1 (200 bp) // ivs12-2 (200 bp) – ex13 (226 bp) – ivs13-1 (200 bp) // ivs13-2 (200 bp) – ex14 (126 bp) – ivs14-1 (200 bp) // ivs14-2 (200 bp) – ex15 (126 bp) – ivs15-1 (200 bp) // ivs15-2 (200 bp) – ex16 (90 bp) – ivs16-1 (200 bp) // ivs16-2 (200 bp) – ex17 (172 bp) – ivs17 (230 bp)**.**

**
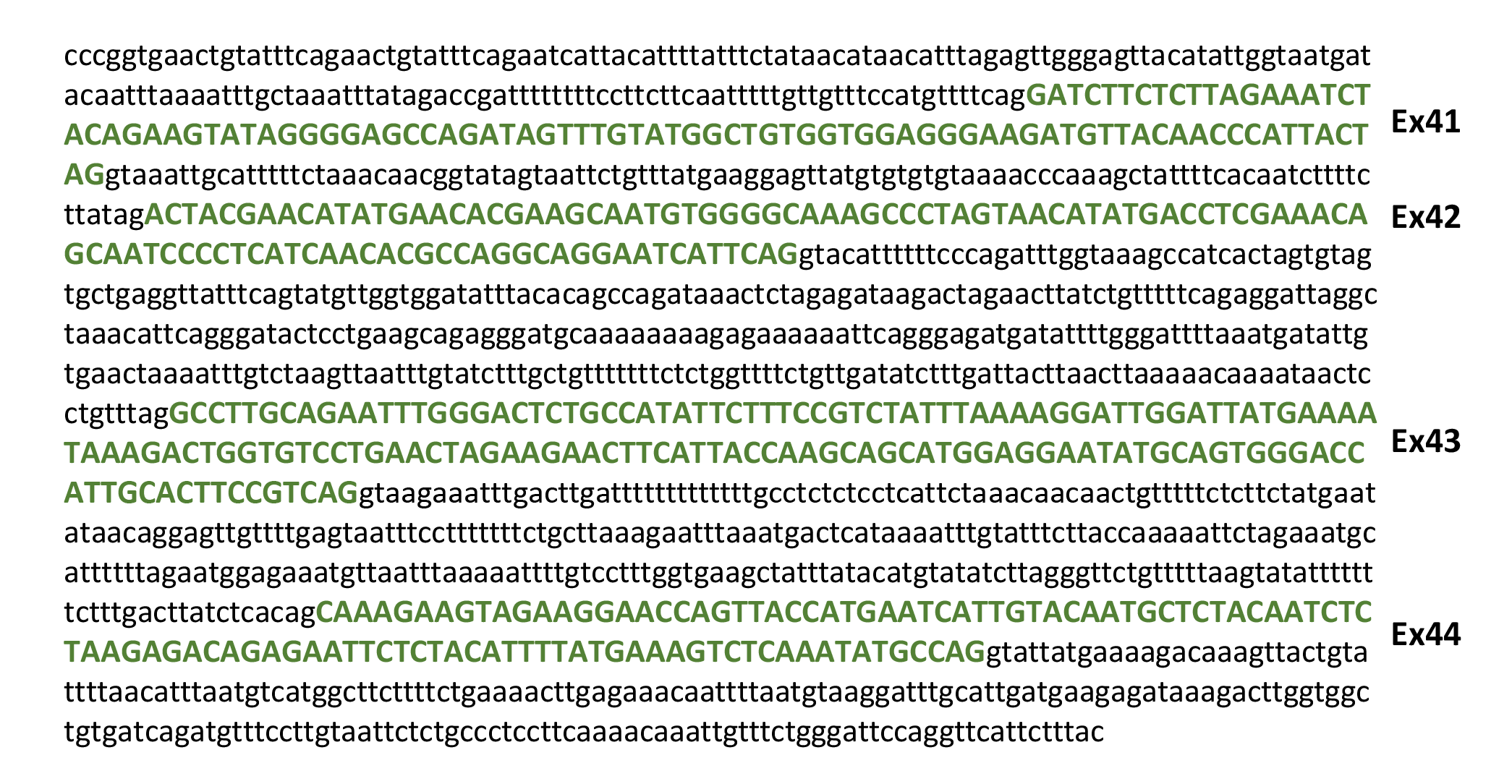
**

**Figure S1B. Insert sequence of minigene mgATM_41–44.** The minigene mgATM_41–44 was assembled in three steps of Overlap Extension PCR cloning [53]. The three consecutive inserts were amplified with Phusion high fidelity polymerase (Fisher Scientific, Waltham, MA, USA) and the primers indicated in supplementary material, Table S2. Firstly, exons 41 and 42 and the corresponding flanking intronic sequences (691 bp) were amplified and cloned into pSAD v9.0. Then, exon 43 (383 bp) and exon 44 (504 bp) were amplified and consecutively cloned to obtain the final minigene mgATM_41–44 (6,031 bp). The final construct was confirmed by sequencing (Macrogen). **Size:** 1,543 bp. **Structure:** ivs40 (170 bp) – ex41 (89 bp) – ivs41 (99 bp) – ex42 (103 bp) – ivs42-1 (200 bp) // ivs42-2 (135 bp) – ex43 (149 bp) – ivs43-1 (94 bp) // ivs43-2 (199 bp) – ex44 (105 bp) – ivs44 (200 bp).

**
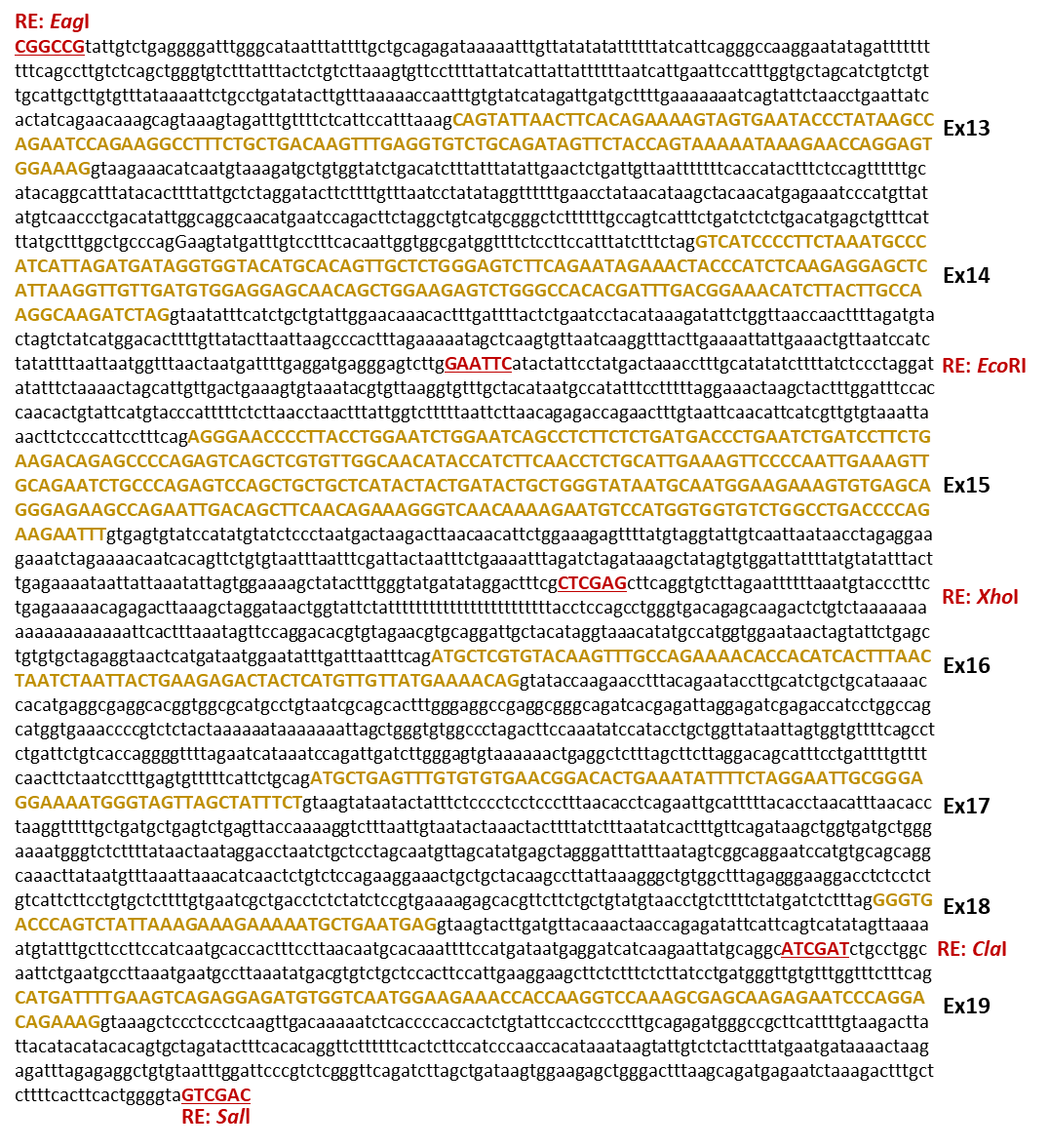
**

**Figure S1C. Insert sequence of minigene mgBRCA1_13–19.** First, BRCA1 exon 15 and the flanking intronic sequences (870 bp) were amplified and cloned into the splicing vector between the restriction sites EcoRI and XhoI. The exon 16-insert (777 bp) was introduced into the previous clone by XhoI/HindIII digestion/ligation to generate the second intermediate minigene. Then, exon 14 (642 bp) was introduced using restriction enzymes BamHI and EcoRI and, subsequently, exons 17 and 18 (1,033 bp- insert) were cloned between the HindIII and ClaI sites. Finally, exon 13 (794 bp) and exon 19-inserts (1,352 bp) were cloned at the EagI/BamHI and ClaI/SalI sites, respectively. To improve the minigene handling, 3 intronic deletions were performed over the original construct, generating the final minigene mgBRCA1_13–19 (8,758 bp). Additional cloning information is available upon request. Cloning sites (EagI, EcoR1, Xhol, ClaI, Sall) are shown in red and underlined. The final construct was confirmed by sequencing (Macrogen). **Size:** 4,316 bp. **Structure:** ivs12 (374 bp) – ex13 (127 bp) – ivs13-1 (200 bp) // ivs13-2 (200 bp) – ex14 (191 bp) – ivs14-1 (250 bp) // EcoRI // ivs14-2 (291 bp) – ex15 (311 bp) – ivs15-1 (268 bp) // XhoI // ivs15-2 (298 bp) – ex16 (88 bp) – ivs16-1 (200 bp) // ivs16-2 (200 bp) – ex17 (78 bp) – ivs17 (500 bp) – ex18 (41 bp) – ivs18-1 (147 bp) // ClaI // ivs18-2 (27 bp) // ivs18-3 (92 bp) – ex19 (84 bp) – ivs19 (331 bp).

**
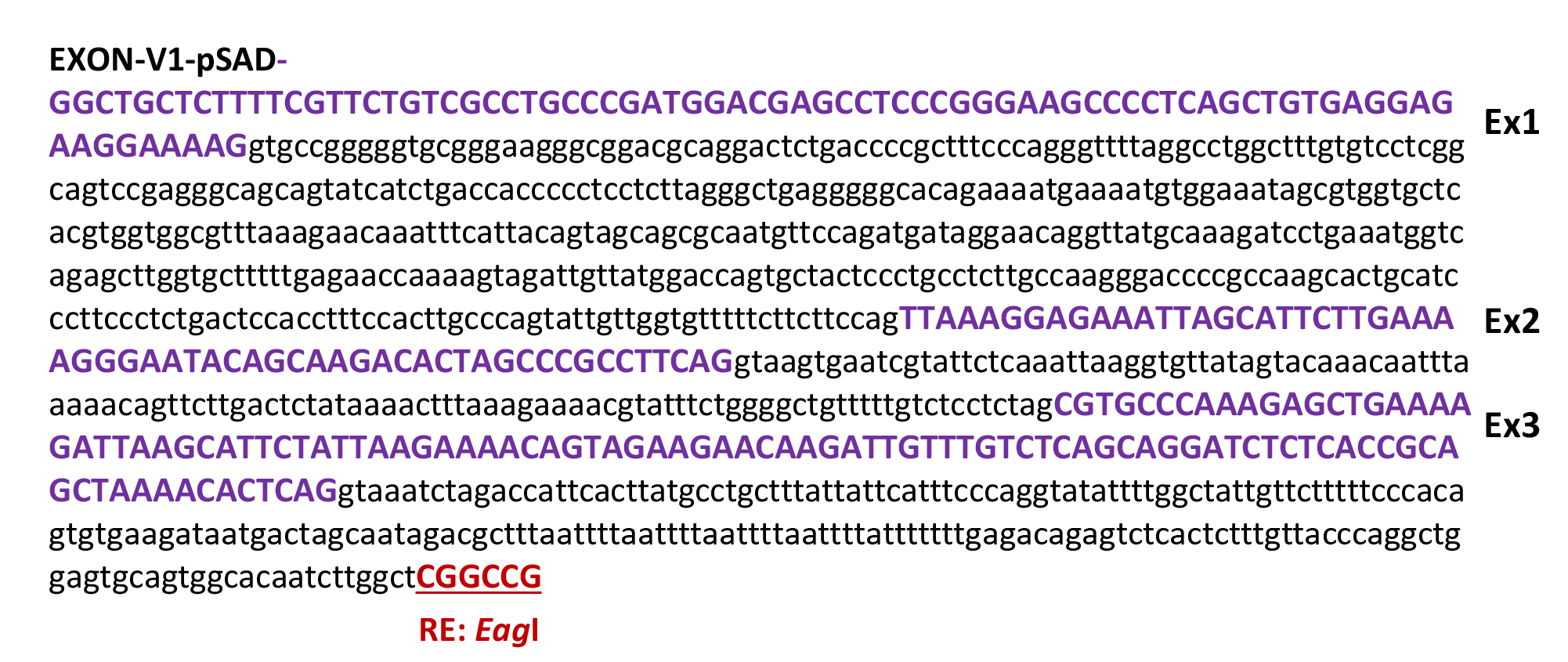
**

**Figure S1D. Insert sequence of minigene mgPALB2_1–3. Size:** 974 bp. **Structure:** ex1 (78 bp) – ivs1-1 (216 bp) // ivs1-2 (200 bp)- ex2 (60 bp) – ivs2 (117 bp) – ex3 (103 bp) – ivs3 (200 bp).

**
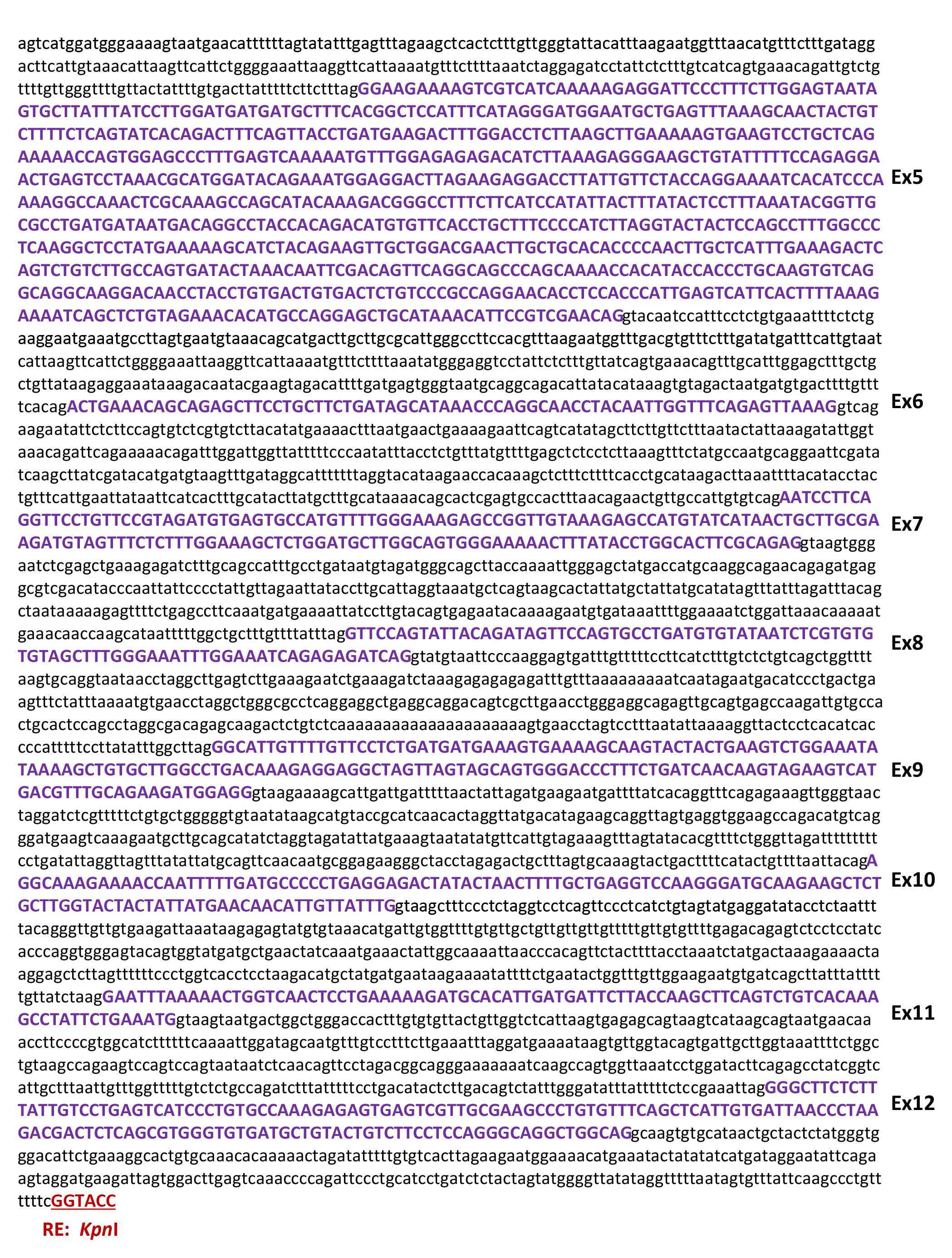
**

**Figure S1E. Insert sequence of minigene mgPALB2_5–12. Size:** 4,947 bp. **Structure:** ivs4 (266 bp) – ex5 (830 bp) – ivs5 (364 bp) – ex6 (72 bp) – ivs6-1 (209 bp) // ivs6-2 (191 bp) – ex7 (162 bp) – ivs7-1 (114 bp) // ivs7-2 (250 bp) – ex8 (86 bp) – ivs8-1 (200 bp) // ivs8-2 (200 bp) – ex9 (162 bp) – ivs9-1 (200 bp) // ivs9-2 (200 bp) – ex10 (117 bp) – ivs10-1 (200 bp) // ivs10-2 (200 bp) – ex11 (88 bp) – ivs11-1 (200 bp) // ivs11-2 (200 bp) – ex12 (149 bp) – ivs12 (250 bp).

**Figure S1. Insert sequences of minigenes mgATM_11–17, mgATM_41–44, mgBRCA1_13–19, mgPALB2_1–3 and mgPALB2_ex5–12.** Exons are indicated in upper case and cloning sites are underlined. The structure of each insert is indicated below each sequence, where a double slash indicates a shortened intron.
